# Seven‐Membered Cyclic Potassium Diamidoalumanyls

**DOI:** 10.1002/chem.202102682

**Published:** 2021-09-21

**Authors:** Ryan J. Schwamm, Michael S. Hill, Han‐Ying Liu, Mary F. Mahon, Claire L. McMullin, Nasir A. Rajabi

**Affiliations:** ^1^ Department of Chemistry University of Bath Claverton Down Bath BA2 7AY UK

**Keywords:** aluminium, coordination modes, main group chemistry, metal-metal interactions

## Abstract

The seven‐membered cyclic potassium alumanyl species, [{SiN^Mes^}AlK]_2_ [{SiN^Mes^}={CH_2_SiMe_2_N(Mes)}_2_; Mes=2,4,6‐Me_3_C_6_H_2_], which adopts a dimeric structure supported by flanking K‐aryl interactions, has been isolated either by direct reduction of the iodide precursor, [{SiN^Mes^}AlI], or in a stepwise manner via the intermediate dialumane, [{SiN^Mes^}Al]_2_. Although the intermediate dialumane has not been observed by reduction of a Dipp‐substituted analogue (Dipp=2,6‐*i*‐Pr_2_C_6_H_3_), partial oxidation of the potassium alumanyl species, [{SiN^Dipp^}AlK]_2_, where {SiN^Dipp^}={CH_2_SiMe_2_N(Dipp)}_2_, provided the extremely encumbered dialumane [{SiN^Dipp^}Al]_2_. [{SiN^Dipp^}AlK]_2_ reacts with toluene by reductive activation of a methyl C(*sp* ^3^)‐H bond to provide the benzyl hydridoaluminate, [{SiN^Dipp^}AlH(CH_2_Ph)]K, and as a nucleophile with BPh_3_ and RN=C=NR (R=*i*‐Pr, Cy) to yield the respective Al‐*B*‐ and Al‐*C*‐bonded potassium aluminaborate and alumina‐amidinate products. The dimeric structure of [{SiN^Dipp^}AlK]_2_ can be disrupted by partial or complete sequestration of potassium. Equimolar reactions with 18‐crown‐6 result in the corresponding monomeric potassium alumanyl, [{SiN^Dipp^}Al−K(18‐cr‐6)], which provides a rare example of a direct Al−K contact. In contrast, complete encapsulation of the potassium cation of [{SiN^Dipp^}AlK]_2_, either by an excess of 18‐cr‐6 or 2,2,2‐cryptand, allows the respective isolation of bright orange charge‐separated species comprising the ‘free’ [{SiN^Dipp^}Al]^−^ alumanyl anion. Density functional theory (DFT) calculations performed on this moiety indicate HOMO‐LUMO energy gaps in the of order 200–250 kJ mol^−1^.

## Graphical abstract

**Seven‐membered** cyclic potassium diamidoalumanyls react with toluene by reductive activation of a methyl C(*sp* ^3^)−H bond and as a nucleophile with BPh_3_ and RN=C=NR (R=*i*‐Pr, Cy) to yield the respective Al‐*B*‐ and Al‐*C*‐bonded potassium aluminaborate and alumina‐amidinate products. The dimeric structure of [{SiN^Dipp^}AlK]_2_ can be disrupted by partial or complete sequestration of potassium with 18‐cr‐6 or 2,2,2‐cryptand to provide either Al−K bonded or charge‐separated species comprising the ‘free’ alumanyl anion.

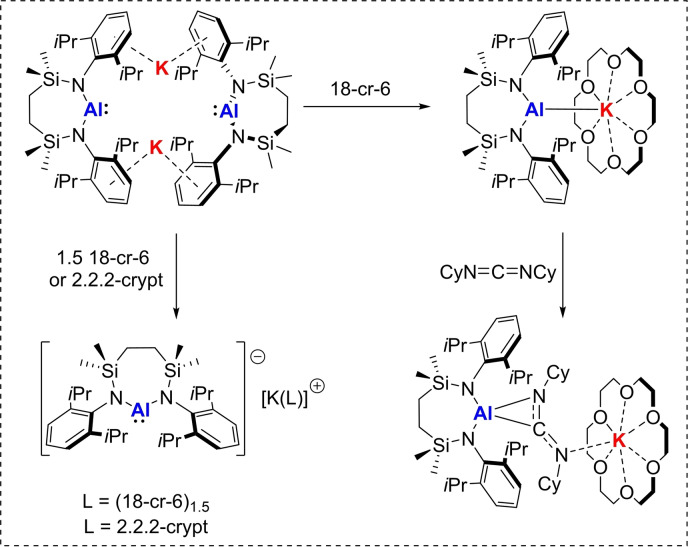

## Introduction

**Figure 1:**
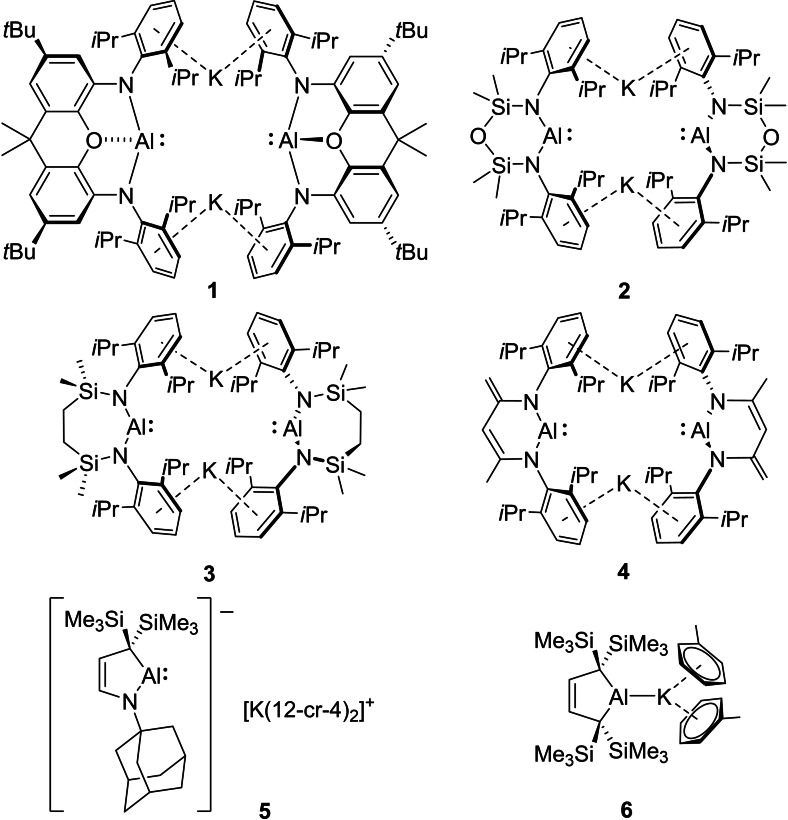
Reported examples of potassium alumanyl species, compounds **1**–**6**.

Although the electrophilic and Lewis acidic compounds arising from its common trivalent state will continue to define its chemistry, the past decade has witnessed significant developments in low oxidation state aluminium chemistry.^[1]^ A majority of these studies have focused on the coordinative and reducing capabilities of a variety of charge neutral cyclopentadienyl and β‐diketiminato Al(I) derivatives.^[2]^ A sequence of anionic species, however, in which the group 13 centre maintains an analogous Al(I) valence state have emerged since 2018. Although its synthesis had been presaged by several broadly analogous boron‐ and gallium‐centered species,[^3^, ^4^] Aldridge, Goicoechea and co‐workers’ 2018 report of the xanthene‐derived potassium alumanyl, [(NON)AlK]_2_ (**1**, where NON=4,5‐bis(2,6‐diisopropyl‐anilido)‐2,7‐di‐tert‐butyl‐9,9‐dimethylxanthene, Figure 1),[^5^, ^6^] realized a long held objective in group 13 element chemistry.[^4^, ^7^] Compound **1** has already displayed startling reactivity toward both organic and metal‐based reagents,[^8^, ^9^, ^10^, ^11^] and its synthesis was rapidly followed by the development of several further dinuclear diamido derivatives (**2**),[^12^, ^13^] (**3**),^[14]^ and (**4**),^[15]^ as well as the charge‐separated alkylamido^[16]^ and Al−K bonded dialkyl[^17^, ^18^] alumanyl variants, compounds **5** and **6**, respectively (Figure 1). While the emergent behaviour of all six species is predicated on their Al‐centered nucleophilicity and their aptitude as potent two electron reductants, this diversity of structure also dictates the relevant frontier orbital energies and provides a potential means to modulate reactivity.[^11^, ^19^] For example, whereas the diamide derivatives **2** and **3** appear stable in benzene, compounds **1**,[^5^, ^10^] **4** ^[15]^ and **6** ^[17]^ can effect the one‐ and even two‐fold (**4**) oxidative addition of arene C−H bonds to provide the corresponding aluminium(III) aryl hydrides. Introduction of 2.2.2‐crypt (2.2.2‐crypt=4,7,13,16,21,24‐hexaoxa‐1,10‐diazabicyclo[8.8.8]hexacosane) to compound **1**, meanwhile, affords the charge separated species [(NON)Al]^−^[K(2.2.2‐crypt)]^+^ (**7**), containing a now 2‐coordinate Al(I) centre which is sufficiently reactive to induce the reversible activation of a benzene C−C bond.[^9^, ^20^]

Our initial interest in this chemistry was prompted by recent observations of the spectacularly reactive systems that arise through the combination of β‐diketiminato calcium cations or hydrides with Roesky's similarly ligated Al(I) species, [(BDI)Al] (BDI=HC{(Me)CN‐Dipp}_2_, where Dipp=2,6‐*i*‐Pr_2_C_6_H_3_).^[21]^ While neither of these earlier reports gave rise to isolable Ca−Al bonded species, combination of compound **3**, [{SiN^Dipp^}AlK]_2_, with [(BDI)AeBPh_4_] (Ae=Mg and Ca) allowed access to the alkaline earth derivatives, [(BDI)AeAl{SiN^Dipp^}], both of which comprised direct Ae−Al bonds.^[14]^ Similarly, we have very recently reported that attachment of the alumanyl anion of compound **3** to carbene‐supported Cu(I) centres confers apparent ambiphilic character to the resultant Cu−Al linkage.^[22]^

Although compound **3** was initially envisaged as a synthetic reagent, its availability also allows us to address more general questions regarding the synthesis, structure and reactivity of such alumanyl species. Aside from compound **4**, which was accessed by addition of [KCH(SiMe_3_)_2_] to [(BDI)Al],^[15]^ the synthesis of the alumanyl derivatives illustrated in Figure 1 has been dependent on potassium reduction of a higher valent aluminium synthon containing the pre‐installed spectator ligand. Access to both compounds **5** and **6** may be achieved by reduction of isolable aluminium(II) intermediates, whereas attempts to synthesize analogous Al−Al bonded species en route to compounds **2** and **3** have been, thus far, unsuccessful. In these cases, preferential two electron reduction of the corresponding aluminium(III) iodide precursors apparently results even for reactions carried out with a substoichiometric quantity of potassium. Similarly, although careful control of reaction stoichiometry facilitates the reduction of [(NON)AlI] to [(NON)Al]_2_, this latter compound is stable to further reduction implying that formation of compound **1** does not require the intermediacy of the corresponding dialumane.^[5]^ Although these observations may reflect an intrinsic feature of the diamide architecture, it is also plausible that the steric demands of the *N‐*Dipp substituent common to all three systems either hinders sufficiently rapid coupling of putative {*N*_2_Al^.^} radical intermediates (i. e. **2** and **3**) or provides such significant kinetic protection of the as formed Al−Al bond that further reduction cannot take place. Similarly, the dinuclear structures of compounds **1**–**3** are an apparent consequence of the high affinity of the potassium cations for the Dipp π‐systems, such that the K−Ar interactions predominate in solution and the solid state. While abstraction of the potassium cations of compound **1** and the formation of **7** required their complete encapsulation by 2.2.2‐crypt, compound **5**, in which kinetic protection is provided by *N*‐adamantyl and *C‐*trimethylsilyl units, employed two equivalents of 12‐crown‐4 to achieve charge separation. Similarly, the protection provided to the Al centre by the *C‐*SiMe_3_ substituents of **6** and its crystallization from toluene dictate its structure as a unique contact ion pair comprising a terminal Al−K interaction [3.4549(5) Å].^[17]^

In this study we address some of these considerations with regard to variations in the structures and reactivity of 7‐membered cyclic diamido alumanyl species derived from, and related to, compound **3**.

## Results and Discussion

### N‐Ar steric influence on diamido‐alumanyl systems

**Scheme 1:**
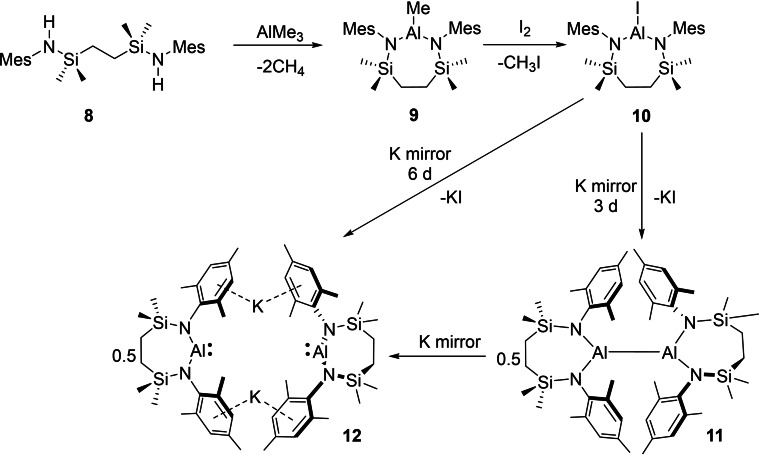
Synthesis of compounds **9**–**12**.

**Figure 2:**
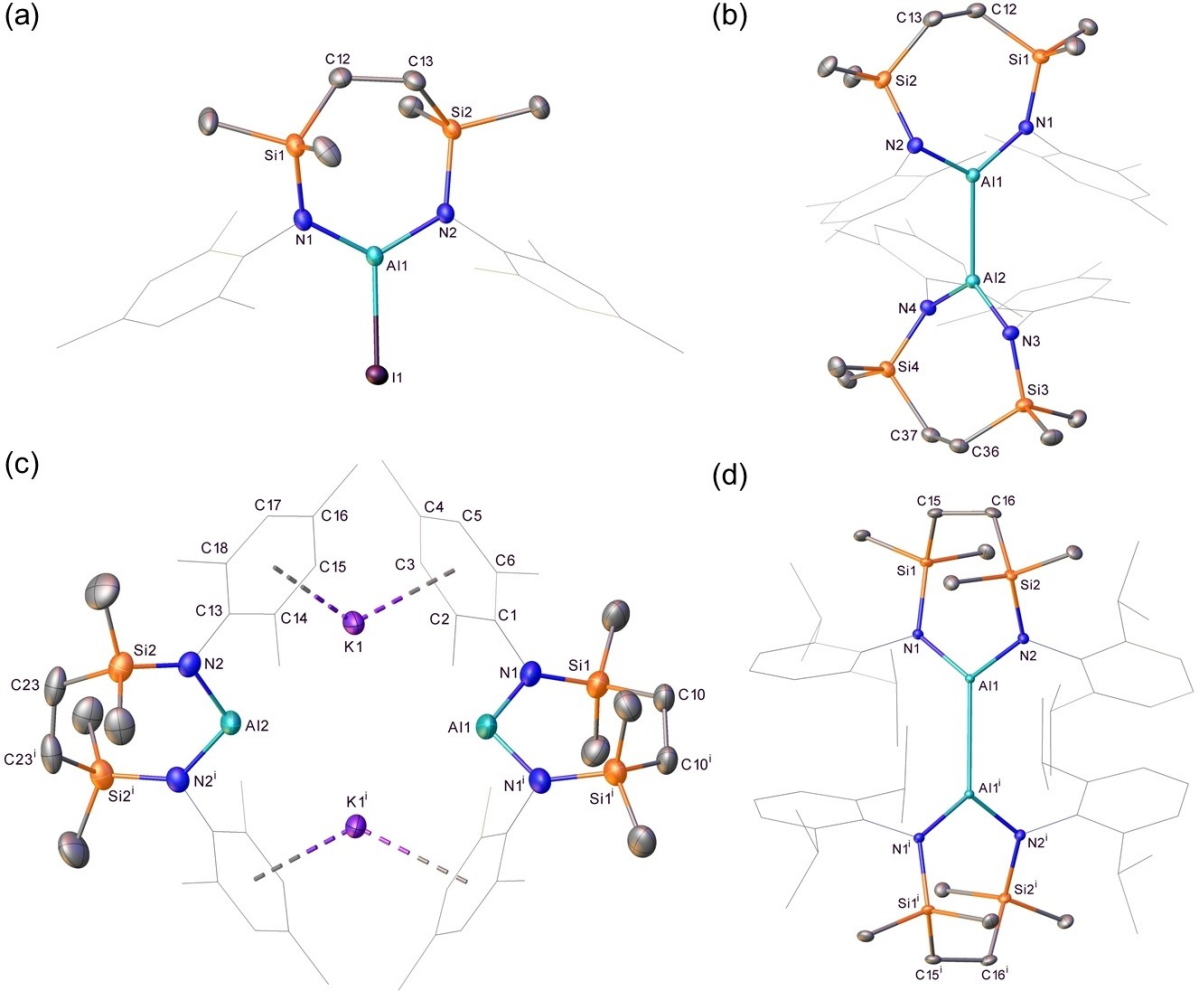
Displacement ellipsoid plots (30 % probability) of (a) compound **10**, (b) compound **11** (c) compound **12** (symmetry operations to generate equivalent atoms, ^i^ 2/3−*x*, 1/3−*x*+*y*, 5/6−*z*), (d) compound **13** (symmetry operations to generate equivalent atoms, ^i^ 3/2−*x*,+*y*, 1−*z*). Solvent molecules, minor components of disordered atoms and hydrogen atoms have been removed for clarity. Wireframe view has been employed for some groups, also for visual simplicity.

As outlined above, all previously reported potassium diamidoalumanyl species, compounds **1**–**4**, feature *N‐*Dipp substituents. While this common feature provides not only a significant level of kinetic stability and impacts profoundly on the dinuclear structures adopted, it also exerts an apparently significant influence over the sequential nature of the Al(III)→Al(II)→Al(I) reductive process during the synthesis of compounds **1**–**3**. In order to examine the consequences of a relaxation of the steric encumbrance about aluminium, the *N‐*mesityl (Mes=2,4,6‐Me_3_C_6_H_2_) variant of the Dipp‐substituted diamide ligand of compound **3** was synthesized by an analogous procedure.^[14]^ Addition of the pro‐ligand [{SiN^Mes^}H_2_] (**8**, {SiN^Mes^}={CH_2_SiMe_2_N(Mes)}_2_) to trimethylaluminium provided [{SiN^Mes^}AlMe] (**9**) in high (97 %) yield (Scheme 1) and the slow conversion of **9** to [{SiN^Mes^}AlI] (**10**, 61 %) was realized by reaction with I_2_ under reflux in toluene over the course of four days. The solid‐state structure of **10** was investigated by single crystal X‐ray diffraction (Figure 2a, Table 1), confirming the formation of a 7‐membered metallacycle that was closely comparable to the previously reported *N*‐Dipp analogue, [{SiN^Dipp^}AlI], albeit with a marginal shortening of the bonds about the planar three‐coordinate aluminium centre [Al−N, 1.782(1) and 1.790(1) vs. 1.773(4), 1.775(4) (**10**); Al−I, 2.4690(5) vs. 2.4596(15) (**10**) Å].

**Table 1:** Selected bond lengths (Å) and angles (°) of compounds **10**–**13**.

	**10**	**11**	**12** ^[f]^	**13** ^[l]^
Al1−N1	1.773(4)	1.8137(12)	1.873(2)	1.8426(10)
	–	1.8107(11)^[b]^	1.872(2)^[g]^	–
Al1−N2	1.775(4)	1.8179(12)	1.8712(16)^[h]^	1.8458(10)
	–	1.8150(11)^[c]^	1.8710(16)^[i]^	–
Al1−X	2.4596(15)^[a]^	2.5850(5)^[d]^	3.703(4)^[j]^	2.7330(6)^m^
Si1−N1	1.757(5)	1.7558(12)	1.7338(19)	1.7796(10)
Si2−N2	1.763(5)	1.7523(12)	1.7274(16)	1.7690(10)
N1−Al1−N2	125.1(2)	118.10(6)	109.73(11)^[k]^	114.28(5)
N1−Al1−X	117.89(16)^[a]^	121.00(4)^[e]^	–	124.21(3)^[m]^
N2−Al1−X	117.03(15)^[a]^	120.53(4)^[e]^	–	121.50(3)^[m]^

[a] X=I1; [b] Al2‐N3; [c] Al2‐N4; [d] X=Al2; [e] X=Al2; [f] symmetry operations to generate equivalent atoms ^l^ 2/3−x, 1/3−x+y, 5/6−z; [g] Al1−N1^1^; [h] Al2−N2; [i] Al2−N2^1^; [j] X=K1^1^; [k] N1−Al1−N1^1^; [l] symmetry operations to generate equivalent atoms ^1^ 3/2−*x*, *y*, 1−*z*; [m] X=Al1^1^.

In contrast to the reduction of its *N‐*Dipp substituted analogue, which provided sole access to the formal Al(I) oxidation level, the outcome of reactions between compound **10** and potassium was found to be significantly influenced by the reaction time (Scheme 1). Stirring of a colourless hexane solution of **10** over a potassium mirror resulted in a gradual colour change to pale yellow and the formation of a grey precipitate over the course of three days. Cessation of the reaction at this point provided a predominant new compound (**11**) in the form of colourless single crystals (71 %), which were suitable for X‐ray diffraction analysis. The results of this analysis (Figure 2b, Table 1) revealed compound **11** to be the dialumane, [{SiN^Mes^}Al]_2_. Although the Al−Al bond of **11** [2.5850(5) Å] is shorter than that observed in Aldridge, Goicoechea and co‐workers’ [(NON)Al]_2_ [2.646(1) Å], in a similar manner to the alumanyl derivative **1**, the Al coordination sphere of this earlier species is augmented through coordination by the oxygen atom of the xanthene ligand.^[5]^ A more appropriate comparison to **10** is, thus, provided by the Al−Al separation in [(dpp‐bian)Al]_2_ (2.522(1) Å, where dpp‐bian=1,2‐bis[(2,6‐diisopropylphenyl)imino]acenaphthene), which comprises identical *N‐*Dipp substitution as **1** but is the only previous diamido dialumane to feature pseudo‐trigonal aluminium coordination.^[23]^ Based on this comparison alone it would appear that the reduction in steric demands associated with use of the {SiN^Mes^} ligand exerts minimal impact on the observed Al−Al bond. Any such appraisal, however, must also take into account the greater constraints enforced on the local Al environment of **11** by its wider N−Al−N angle (118.10(6)° vs. *ca*. 93° in [(dpp‐bian)Al]_2_), which is imposed as a natural consequence of its seven‐membered cyclic structure.

Stirring the iodide precursor [{SiN^Mes^}AlI] (**10**) over a potassium mirror for a more extended period of time (6 days) than that employed in the production of compound **11** provided a bright yellow solution (Scheme 1). Although crystallization of this reaction product from a concentrated Et_2_O solution at −30 °C also provided bright yellow X‐ray quality single crystals of a new species, compound **12**, bulk samples were invariably contaminated with quantities of the colourless Al(II) derivative (**11**). The results of the X‐ray diffraction analysis of compound **12** (Figure 2c, Table 1), however, revealed a structure reminiscent of the previously reported dinuclear alumanyl derivatives, **1**–**4**, in comprising two ‘Al{SiN^Mes^}K’ units linked by potassium⋅⋅⋅π‐aryl interactions [K⋅⋅⋅C range 3.205(5)–3.482(5) Å]. Although the closest Al⋅⋅⋅K distance in **12** [3.529(5) Å] is significantly shorter than the analogous measurements within compounds **2** [3.5916(8) Å]^[12]^ and **3** [3.584(1) Å],^[14]^ it is longer than the more contracted Al−K contacts in the similarly dinuclear **4** [3.499(1) Å]^[15]^ and the terminally bound Al−K(toluene)_2_ unit of **6** [3.4549(5) Å].^[17]^ Consistent with the assignment of a formal Al(I) oxidation state to this species, the Al−N bond distances in **12** [1.8710(16) ‐ 1.873(2) Å] are only marginally shorter than those of the analogous *N‐*Dipp alumanyl (**3**) [1.887(2) ‐ 1.892(2) Å] but are significantly longer than those observed in either its respective Al(III) or Al(II) precursors, compounds **10** [1.773(4), 1.775(4) Å] and **11** [range 1.8107(11)–1.8179(12) Å]. Despite the nominal relaxation in the *N‐*aryl substituent steric demands, the bite angles imposed by chelation of the {SiN^Mes^} ligand [**12**: N1’−Al1−N1 109.73(11), N2−Al2−N2’ 112.33(10)°] are even wider than those subtended by the *N‐*Dipp diamide environment of **3** [108.84(9), 108.77(9)°], albeit these angles within both sets of 7‐membered aluminacycles are significantly more obtuse than those within the 6‐membered alumanyl derivative, compound **2** [103.89(8), 105.05(8)°].^[12]^

**Scheme 2:**
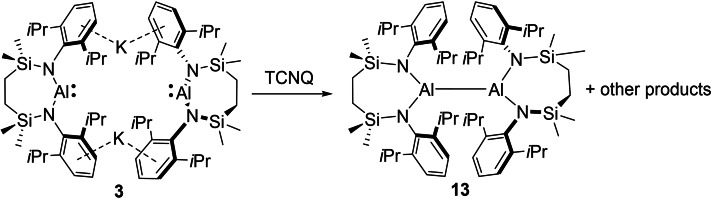
Synthesis of compound **13** by oxidation of **3**.

Although the Al(II) derivative, compound **11**, could also be reacted with a potassium mirror to provide quantities of **12**, we have not yet succeeded in the synthesis of the *N*‐Dipp substituted analogue of compound **11**, [{SiN^Dipp^}Al]_2_ (**13**) by reduction of the corresponding Al(III) iodide precursor. Single crystals of compound **13**, however, were found to be accessible by treatment of the alumanyl derivative **3** with the organic electron acceptor 7,7,8,8‐tetracyanoquinodimethane (TCNQ, *E* ^0^=+0.2 V vs. SCE or Ag/AgCl)^[24]^ (Scheme 2), presumably with concurrent formation of the TCNQ radical anion. Although pure bulk quantities of compound **13** could not be obtained, and Jones and co‐workers have previously demonstrated that treatment of analogous N‐heterocyclic gallyl anions with ferrocenium ion or Tl_2_SO_4_ provides synthetic access to Ga−Ga bonded digallanes,^[25]^ the isolation of **13** provides, to the best of our knowledge, a rare example of an oxidative approach to Al−Al bond formation.^[2i]^ Although the centrosymmetric structure of compound **13** (Figure 2d, Table 1) displays a pronounced narrowing of the N−Al−N bond angle [114.28(5)°] in comparison to that of **11**, we suggest that this structural adjustment is a likely consequence of the much greater crowding imposed about the Al−Al bond, which is also reflected in the pronounced elongation of this distance to 2.7330(6) Å.

### Reactivity of compound 3

**Scheme 3:**
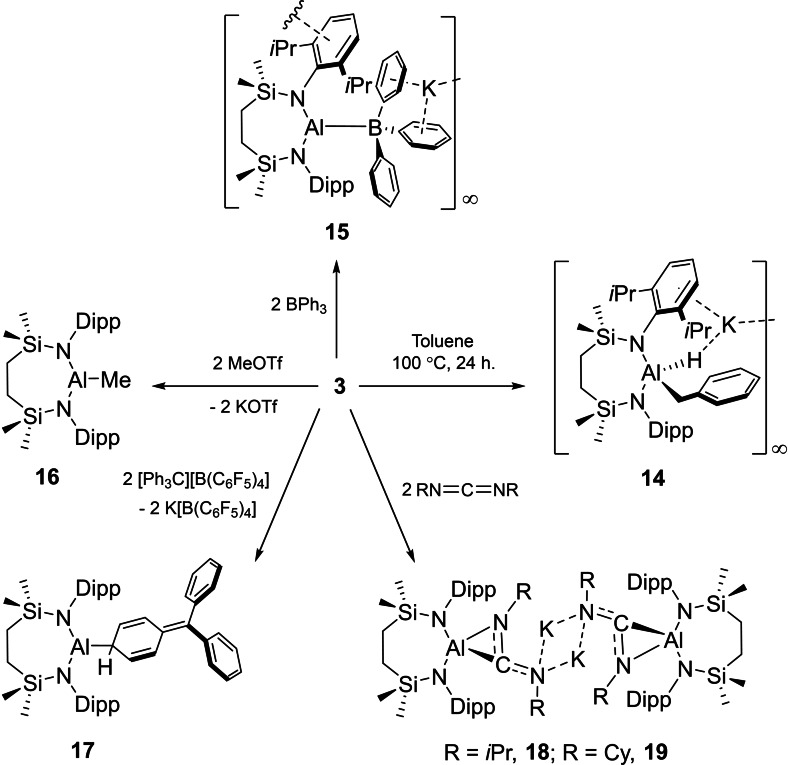
Synthesis of compounds **14**–**19** from **3**.

**Figure 3:**
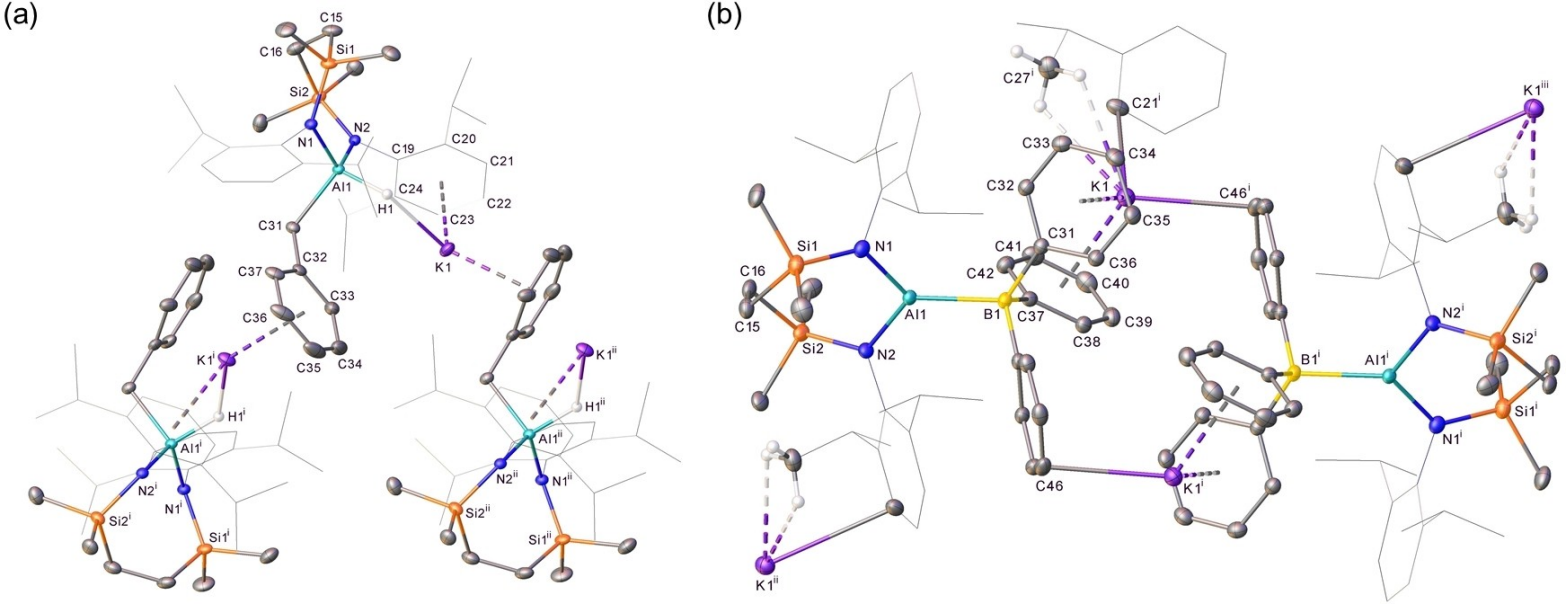
Displacement ellipsoid plots (30 % probability) of the polymeric structures of (a) compound **14** (symmetry operations to generate equivalent atoms, ^i^ 1/2−*x*, −1/2+*y*, 3/2−*z*; ^ii^ 1/2−*x*, 1/2+*y*, 3/2−*z*) (b) compound **15** (symmetry operations to generate equivalent atoms, ^i^ −1/2+*x*, 3/2−*y*, −1/2+*z*; ^ii^ 1−*x*,1−*y*,1−*z*; ^iii^ 1/2+*x*, 3/2−*y*, 1/2+*z*). Minor components of disordered atoms and hydrogen atoms have been removed for clarity. Wireframe view has been employed for some groups, also for visual simplicity.

As well as the aforementioned C−C and C−H activation of benzene, compounds **1** and **6** have recently been reported to effect the C−H activation of mono‐ and di‐substituted arenes.[^10^, ^20^, ^26^] Both compounds provided the aryl aluminium hydride products of *meta*‐C−H activation when reacted with *n*‐butylbenzene in processes that were attributed to the lower barrier heights associated with the nucleophilic formation of the relevant Meisenheimer‐type intermediates. While compound **6** also provided perfectly *meta‐*selective activation of toluene, heating of a toluene solution of **1** at 80 °C resulted in the generation of a co‐crystallized mixture of the *meta‐*aryl and benzylic aluminium hydride products in a respective 3 : 1 ratio. In contrast to this preference for C(sp^2^) bond activation, heating of a d_8_‐toluene solution of compound **3** to 110 °C for 24 h gave selective activation of a methyl C−H bond to provide stoichiometric conversion to the benzylaluminium hydride product, [{SiN^Dipp^}AlH(CH_2_Ph)]K (**14**). The formation of compound **14** was clearly apparent in the resultant ^1^H NMR spectrum through a desymmetrization of the ligand backbone and a splitting of the Si‐*Me* environments into two (6H) singlet resonances at δ −0.04 and −0.05 ppm resulting from the loss of a plane of symmetry through the chelated {SiN^Dipp^} ligand (Scheme 3). These inferences were confirmed by a subsequent single crystal X‐ray analysis of **14**, which revealed a polymeric array of 4‐coordinate diamido aluminate anions propagated via a series of Al−H−K, K‐η^6^‐Dipp and K‐η^6^‐benzyl interactions (Figure 3a, Table 2).

**Table 2:** Selected bond lengths (Å) and angles (°) of compounds **14**, **15** and **17**–**19**.

	**14**	**15**	**17**	**18**	**19**
Al1−N1	1.8694(16)	1.863(2)	1.7964(11)	1.8482(11)	1.8586(13)
Al1−N2	1.8790(16)	1.874(2)	1.7990(12)	1.8502(11)	1.8499(13)
Al1−X	2.036(2)^[a]^	2.190(3)^[b]^	2.0074(14)^[a]^	1.9790(13)^[a]^	1.9786(16)^[a]^
	–	–	–	1.8568(11)^[c]^	1.8519(13)^[c]^
Si1−N1	1.7389(17)	1.747(2)	1.7535(12)	1.7432(11)	1.7485(13)
Si2−N2	1.7286(17)	1.747(2)	1.7520(12)	1.7409(11)	1.7385(13)
N1−Al1−N2	111.78(8)	111.34(10)	121.14(5)	114.04(5)	112.78(6)
N1−Al1−X	110.83(8)^[a]^	127.75(11)^[b]^	124.67(6)^[a]^	118.14(5)^[c]^	120.90(6)^[c]^
N2−Al1−X	110.64(8)^[a]^	120.84(11)^[b]^	114.12(6)^[a]^	120.04(5)^[c]^	117.59(6)^[c]^

[a] X=C31; [b] X=B1; [c] X=N3.

We have previously reported that the potassium alumanyl, compound **3**, may be utilized in reactions with electrophilic metal reagents to provide terminal Mg−Al, Ca−Al and Cu−Al bonds.[^14^, ^22^] The generality of this nucleophilic character was further assessed by reaction of **3** with a variety of carbon and boron electrophiles. Treatment of **3** with triphenylborane gave clean access to a single new compound (**15**, 67 %) (Scheme 3). While sparingly soluble in arene solvents, solutions of compound **15** in CDCl_3_ provided ^1^H and ^13^C{^1^H} NMR spectra comprising a single set of *i*‐Pr signals, indicative of significant levels of free rotation within the molecule. Consistent with a now non‐spherically symmetric environment if bonded to a similarly quadrupolar ^27^Al atom (*I*=5/2; 100 %), no resonance indicative of the formation of 4‐coordinate boron could be observed in the corresponding ^11^B NMR spectrum. The assignment of **15** as a potassium aluminoborate species, however, was confirmed by X‐ray diffraction analysis of a single crystal grown from a saturated toluene solution (Figure 3b, Table 2). Concordant with its low solubility in non‐polar solvents, the structure of **15** comprises a network of borate anions propagated by a variety of polyhapto B−Ph⋅⋅⋅K and N‐Dipp⋅⋅⋅K bridging interactions. Despite the formal anionic charge borne by the borate component of **15**, the Al1−B1 distance [2.190(3) Å] lies only marginally outside the range of Al−B bond lengths [2.1147(15)–2.183(3) Å] established for the handful of charge neutral cyclopentadienyl and β‐diketiminato aluminium(I) adducts of B(C_6_F_5_)_3_ and 9‐borafluorenes that have been described.^[27]^

**Figure 4:**
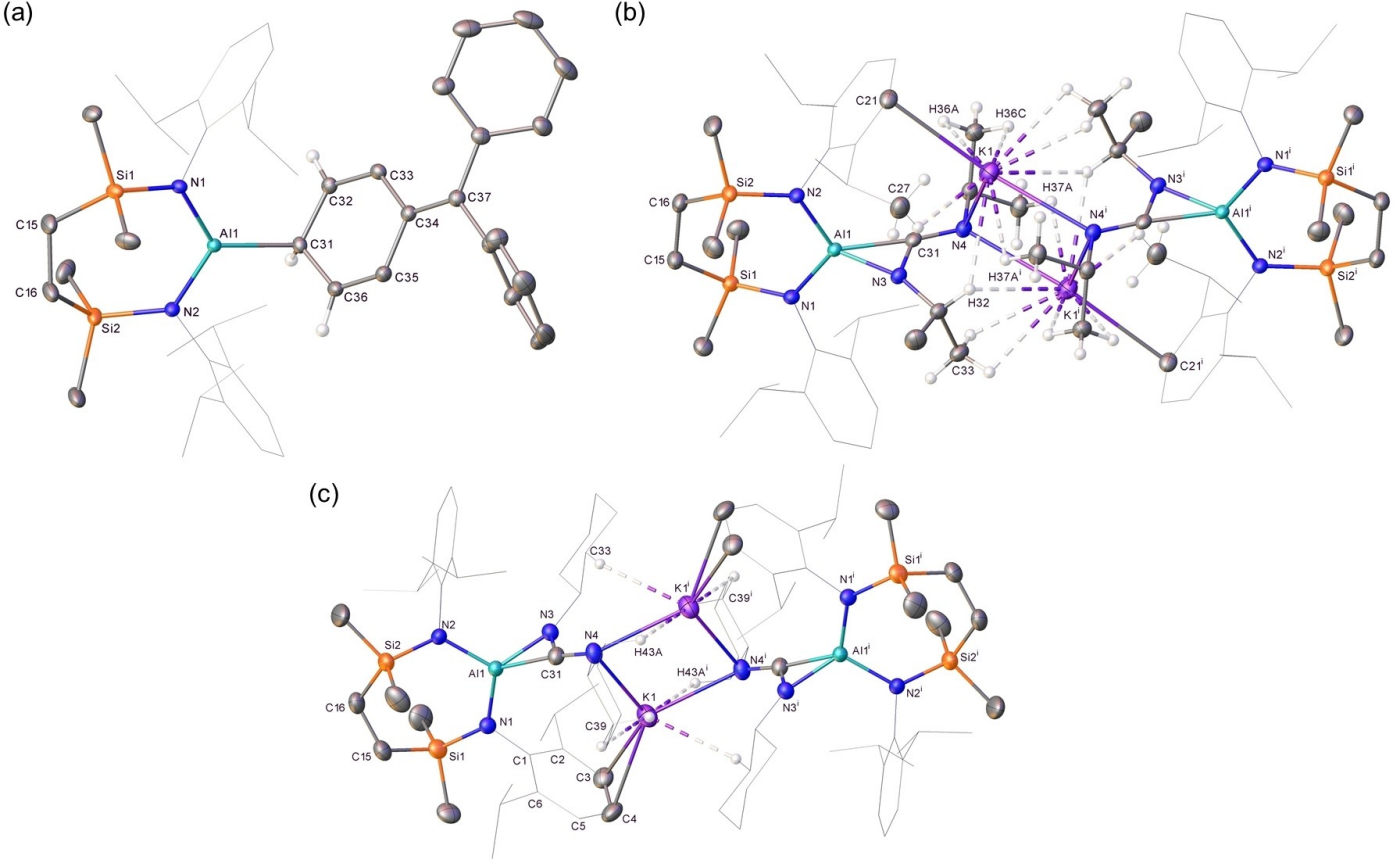
Displacement ellipsoid plots (30 % probability) of (a) compound **17**, (b) compound **18** (symmetry operations to generate primed atoms, ^i^ *x*, −*y*, −*z*) (c) compound **19** (symmetry operations to generate primed atoms, ^i^ 2−*x*, 1−*y*, 1−*z*). Minor components of disordered atoms, solvate and hydrogen atoms (except those of significance) have been removed for clarity. Wireframe view also has been employed for some groups to afford visual simplicity.

In a similar manner to the previously reported reactivity of compound **1**,^[5]^ reaction of **3** with MeOTf provided the diamidomethylalumane, [{SiN^Dipp^}AlMe] (**16**), which was readily identified by ^1^H and ^13^C NMR spectroscopy.^[14]^ In contrast to this straightforward behaviour, reaction of **3** with the BPh_3_‐isoelectronic trityl cation of [Ph_3_C][B(C_6_F_5_)_4_] demonstrated a series of rapid colour changes to green to bright red to light yellow within 10 minutes and the deposition of a crystalline material that was identified as K[B(C_6_F_5_)_4_] by a unit cell check (Scheme 3).^[28]^ Assessment of the remaining solution by ^1^H NMR spectroscopy indicated the formation of a predominant new compound (**17**, 53 %) characterized by the appearance of two mutually coupled doublet of doublets resonances and a triplet signal at δ 5.99, 4.39 and 2.09 ppm, respectively. The origin of these observations was resolved by X‐ray diffraction analysis (Figure 4a) that confirmed **17** to be a neutral diamidoalumane bearing a cyclohexadienyl substituent with an Al1−C31 bond length of 2.0074(14) Å. The cyclohexadienyl‐moiety of **17** is evidenced by the short C32−C33 and C35−C36 bond distances of 1.340(2) Å and 1.340(2) Å, the C34−C37 bond length of 1.369(2) Å and the sum of angles at C37 of 359.6°. Although the formation of this moiety is, thus, strongly reminiscent of the methylenecyclohexadiene structure resulting from the unsymmetrical coupling of the trityl (Gomberg) radical, the observation of the potassium borate by‐product leads us to suggest that this reactivity is a result of alumanyl anion metathesis in a similar manner to that observed with MeOTf.

Compound **3** reacted cleanly with *N*,*N*’‐di‐*iso*propyl carbodiimide and *N*,*N*’‐di‐cyclohexyl carbodiimide through Al−C bond formation. (Scheme 3). Although the resultant compounds, **18** (74 %) and **19** (70 %), are, to the best of our knowledge, unique examples of alumina‐amidinate anions, their formation may be considered analogous to the bora‐amidinate anions that have resulted from the addition of carbodiimides to a variety of magnesium derivatives of similarly nucleophilic boryl units.^[29]^ The single crystal X‐ray structures of compounds **18** and **19** are illustrated in Figures 4b and c, while selected metric data are provided in Table 2. Both compounds crystallize as centrosymmetric dimers propagated via two‐fold N−K−N bridging interactions through the 4‐coordinate N4 atoms of the alumina‐amidinate anions, augmented by a variety of close contacts with the amidinato *N‐i*‐Pr and *N*‐Cy residues. Although the delocalised nature of the N3−C31−N4 units is indicated by the C31−N3 [**18**, 1.3782(17); **19**, 1.370(2) Å] and C31−N4 [**18**, 1.3145(17); **19**, 1.320(2) Å] bond lengths, the diamidoalumane components of both molecules interact in a η^2^‐fashion with both C31 [C31−Al1: **18**, 1.9790(13); **19** 1.9786(16) Å] and N3 [N3−Al1: **18**, 1.8568(11); **19**, 1.8519(13) Å] such that the aluminium centres display distorted tetrahedral {*N*_3_ *C*Al} coordination geometries.

### Alumanyl dimer cleavage

**Figure 5:**
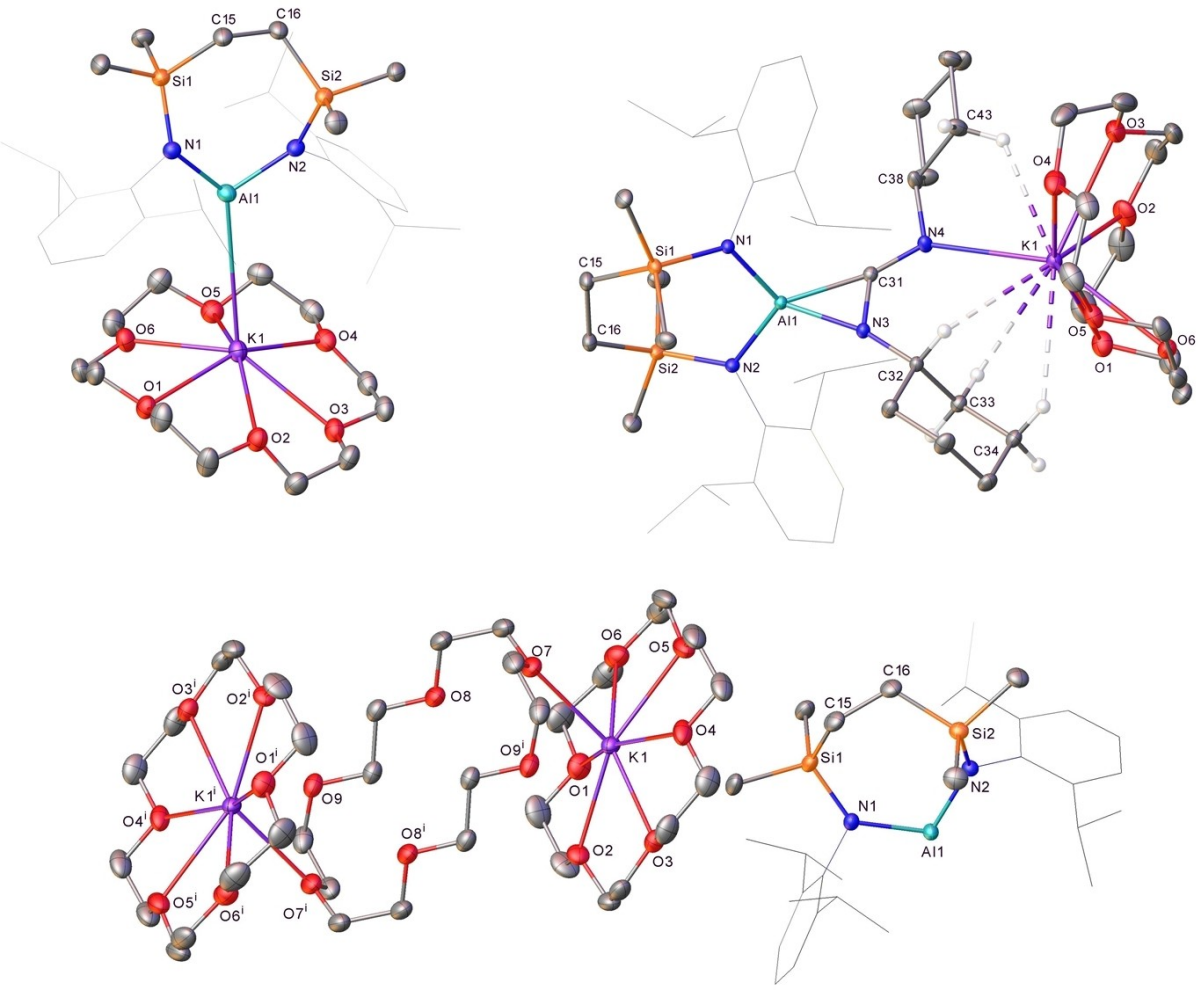
Displacement ellipsoid plots (30 % probability) of (a) compound **20**, (b) compound **21** and (c) compound **22** (symmetry operations to generate primed atoms, ^i^ −*x*, 1−*y*, −*z*). Minor components, disordered atoms, solvate and hydrogen atoms (except those of significance) have been removed for clarity. Wireframe view has also been employed for some groups in the interests of visual simplicity.

Compound **3** was also treated with a stoichiometric quantity of 18‐crown‐6 (18‐cr‐6) in toluene (Scheme 3). Removal of solvent and crystallization of the resultant orange oil from methylcyclohexane provided orange crystals of the mononuclear crown ether complex, [{SiN^Dipp^}Al−K(18‐cr‐6)], compound **20** (74 %). The ^1^H NMR spectrum of **20** in C_6_D_6_ revealed a significant shift in the Si*Me_2_* resonance (δ 0.49 ppm) compared to the respective data provided by **3**, consistent with a change in the solution‐state structure. Single crystal X‐ray diffraction analysis revealed that **20** comprises a distorted trigonal planar aluminium centre (Figure 5a, Table 3), in which the aluminium coordination environment is provided by the bidentate diamide ligand and, in a similar manner to compound **6**, a terminal Al−K contact. The absence of the K⋅⋅⋅π‐aryl interactions observed in compounds **3** and **12** induces an elongation of the Al−N bonds in **20** [1.9213, 1.9217(5) Å] and a pronounced narrowing of the N−Al−N bond angle [105.93(6)°]. The Al−K(18‐cr‐6) interaction [3.9133(6) Å] is significantly longer than that observed in compound **6** [3.4549(5) Å], the only previously described species to feature a similar terminal aluminium‐potassium bond.^[17]^

**Table 3:** Selected bond lengths (Å) and angles (°) of compounds **20**–**23**.

	**20**	**21**	**22**	**23**
Al1−N1	1.9210(14)	1.8561(9)	1.9311(13)	1.921(2)
Al1−N2	1.9217(15)	1.8595(9)	1.9163(13)	1.928(2)
Al1−X	3.9134(6)^[a]^	1.8406(9)^[b]^ 1.9471(11)^[c]^	–	–
Si1−N1	1.7288(14)	1.7285(9)	1.7253(13)	1.726(2)
Si2−N2	1.7315(14)	1.7323(9)	1.7220(13)	1.715(2)
N1−Al1−N2 N1−Al1−X N2−Al1−X	105.93(6) 135.46(5)^[a]^ 117.22(4)^[b]^	111.61(4) 126.92(4)^[b]^ 113.39(4)^[b]^	105.69(6) ‐ ‐	105.92(10) – –

[a] X=K1; [b] X=N3; [c] X=C31.

**Scheme 4:**
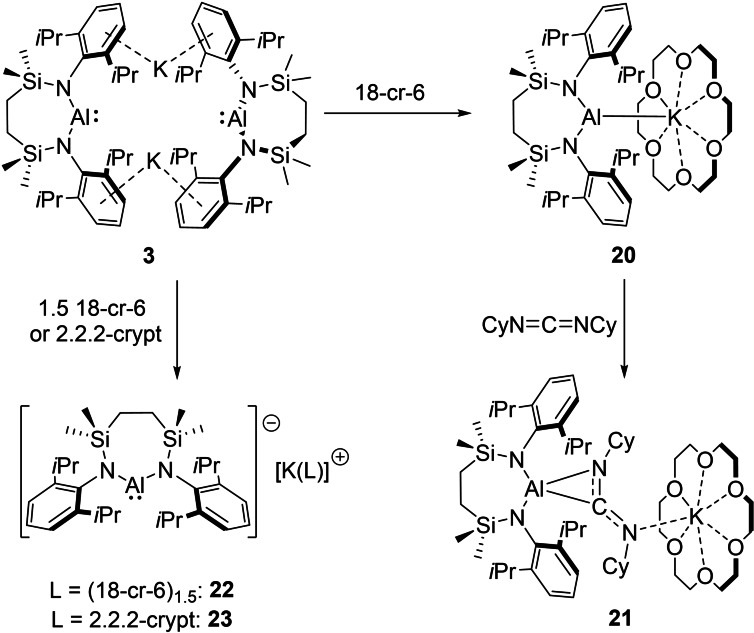
Synthesis of compounds **20**–**23** from **3**.

To exclude the possibility that the long Al−K distance is the result of an undetected bridging *μ*‐hydride moiety, the reaction of **20** with *N,N*′‐dicyclohexylcarbodiimide was performed resulting in the formation of [{SiN^Dipp^}Al−C(NCy)_2_K(18‐cr‐6)] (**21**, 57 %, Scheme 4) as a colourless crystalline solid. Single crystal X‐ray diffraction analysis of **21** confirmed its constitution as a further potassium alumina‐amidinate, where the carbodiimide fragment is bridging the Al and K centres (Figure 5b, Table 3). As observed in the structures of both compounds **18** and **19**, the aluminium centre of **21** coordinates through an η^2^‐interaction with the C31‐N3 bond of the CN_2_ fragment forming a constrained three‐membered Al−C−N metallacycle with Al1−C31, Al1−N3 and C31−N3 distances of 1.9471(1), 1.8406(9) and 1.3790(13) Å, respectively. The 18‐cr‐6 ligated potassium atom is coordinated by a single nitrogen atom of the CN_2_ fragment, with a K−N distance of 2.890(1) Å.

Complete sequestration of the potassium ions of compound **3** was achieved by reaction with either an excess of 18‐cr‐6 or 2.2.2‐crypt in benzene (Scheme 4). While orange crystals of the crown ether derivative [{SiN^Dipp^}Al][K(18‐cr‐6)_1.5_] (**22**, 71 %) deposited directly from the reaction solution, addition of the cryptand resulted in the immediate formation of two immiscible phases. Although this latter observation is characteristic of the generation of charge separated metalorganic species and consequent liquid clathrate formation in arene solvents,^[30]^ orange crystals of [{SiN^Dipp^}Al][K(2.2.2‐crypt)] (**23**, 86 %) were deposited after removal of volatiles and crystallization of the resultant orange oil from tetramethyltetrahydrofuran. The connectivity of the anions in both compounds was similarly unambiguous and the formation of two mononuclear and charge separated forms of the [{SiN^Dipp^}Al]^−^ anion was confirmed by single crystal X‐ray diffraction analysis (Figure 5c, Table 3 and Figure S35). The gross constitution of compounds **22** and **23** differs primarily in the identity of their potassium cations. While the [K(2.2.2‐crypt)]^+^ unit of **23** is effectively identical to that of the sole analogous monomeric alumanyl, [(NON)Al][K(2.2.2‐crypt)] (**7**),^[9]^ the asymmetric unit of **22** contains a K(18‐cr‐6)_1.5_ cation generated by inversion into a dicationic [(18‐cr‐6)K(μ‐18‐cr‐6)K(18‐cr‐6)]^2+^ unit.^[4b]^ The terminal crown ether ligands of this latter moiety are κ^6^‐coordinated to a potassium atom, whilst the bridging crown is κ^2^‐coordinated to each K centre. Despite the absence of any Al⋅⋅⋅Al or Al⋅⋅⋅K contacts, the metric parameters in **23** are closely comparable to the analogous contact ion paired derivative (**20**) [Al1−N1 1.9311(13) (**22**), 1.922(2) (**23**) Å; Al1−N2 1.9163(13) (**22**) 1.927(2) (**23**) Å; N1−Al1−N2 105.69(6)° (**22**) 105.95(11)° (**23**)].

**Figure 6:**
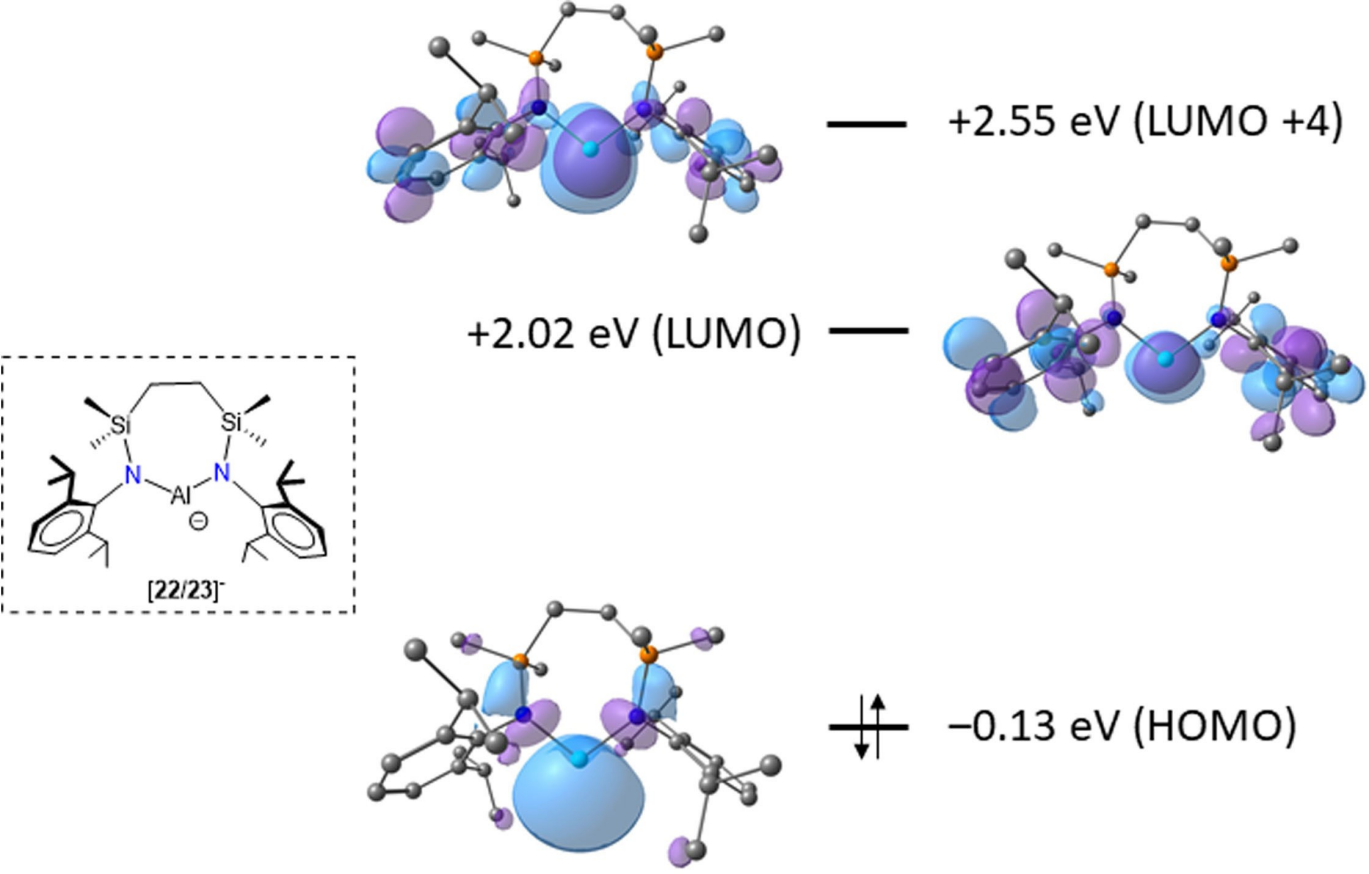
Calculated (BP86) HOMO, LUMO and LUMO+4 energies and associated Kohn‐Sham orbitals for the anionic component of compounds **22** and **23**.

The isolation of compounds **22** and **23** affords an opportunity to assess the electronic structure of the free anion derived from compound **3**. Density functional theory (DFT/BP86/6‐31G**&SDDALL) calculations performed on the mononuclear anionic component of **22**/**23** (i. e. [{SiN^Dipp^}Al]^−^) replicated the experimentally observed Al−N bond lengths and the N−Al−N angle [105.2°]. In common with previously reported calculations on di(amido)alumanyl species, the HOMO approximates to a lone pair on aluminium (Figure 6).^[11]^ The LUMO is primarily ligand based, whereas the empty Al 3*p*_z_‐orbital is mesomerically raised in energy by the π‐donor amide substituents and is represented by the LUMO+4 to provide respective HOMO‐LUMO and HOMO‐LUMO+4 energy gaps of 2.15 and 2.68 eV (207.4 and 258.6 kJ mol^−1^). While these values are significantly lower than a previously reported assessment of the same anion at a different level of theory (DFT‐D3/PBE0/Def‐TZVP: HOMO‐LUMO 3.55 eV; HOMO‐LUMO+4 3.90 eV),^[11]^ and we have not explored these effects any further, our current estimate would place any associated absorption in the blue region of the electromagnetic spectrum consistent with the observation of the complementary orange colour of both compounds **22** and **23**.

## Conclusion

In conclusion, we report the potassium alumanyl species **12** supported by diamide ligands with a reduced steric profile at the flanking nitrogen substituents. Although compound **12** may be synthesized directly from the iodide precursor (**10**), it may also be achieved in a stepwise manner and reduction of the intermediate dialumane (**11**). While the analogous intermediate dialumane was not observed in the reduction process for Dipp‐substituted analogues, partial oxidation of the potassium alumanyl **3** gave access to the extremely encumbered dialumane **13**. Analogous to previously reported diamidoalumanyl species, **12**, adopts a dimeric structure supported by flanking K‐aryl interactions. In common with previously reported alumanyl derivatives, the reactivity of **3** is characterized by its behaviour as a reducing agent and as an Al‐centered nucleophile. The importance of the dimeric structure of **3** has been explored by partial or complete sequestration ofits potassium cations. Use of one equivalent of 18‐crown‐6 results in the corresponding monomeric potassium alumanyl **20**, which provides a rare example of a direct Al−K contact. Complete encapsulation of the potassium cation of **3** either by an excess of 18‐cr‐6 or 2,2,2‐cryptand allows the respective isolation of **22** and **23**, comprising the ‘free’ alumanyl anion, [{SiN^Dipp^}Al]^−^.

## Experimental Section

**Crystal‐structure determination**: Deposition Numbers 2097944 (**10**), 2097945 (**11**), 2097946 (**12**), 2097947 (**13**), 2097948 (**14**), 2097949 (**15**), 2097950 (**17**), 2097951 (**18**), 2097952 (**19**), 2097953 (**20**), 2097954 (**21**), 2097955 (**22**), 2097956 (**23**) contain the supplementary crystallographic data for this paper. These data are provided free of charge by the joint Cambridge Crystallographic Data Centre and Fachinformationszentrum Karlsruhe Access Structures service.

## Conflict of interest

The authors declare no conflict of interest.

## Supporting information

As a service to our authors and readers, this journal provides supporting information supplied by the authors. Such materials are peer reviewed and may be re‐organized for online delivery, but are not copy‐edited or typeset. Technical support issues arising from supporting information (other than missing files) should be addressed to the authors.

Supporting Information
