# Supplementary material for: Seven‐Membered Cyclic Potassium Diamidoalumanyls
Source: Chemistry. 2021 Sep 21;27(60):14971–80. doi: 10.1002/chem.202102682 (PMC8596455; doi:10.1002/chem.202102682)
Supplement: Supplementary file 1 — Supporting Information [file CHEM-27-14971-s001.pdf]

Chemistry–A European Journal

Supporting Information

Seven-Membered Cyclic Potassium Diamidoalumanyls

Ryan J. Schwamm, Michael S. Hill,* Han-Ying Liu, Mary F. Mahon,* Claire L. McMullin,* and Nasir A. Rajabi

General Experimental Details

All manipulations were carried out using standard Schlenk line and glovebox techniques under an inert atmosphere of argon. NMR spectra were recorded on an Agilent ProPulse spectrometer operating at 500 MHz (^{1}H), 126 MHz (^{13}C) and 160 MHz (^{11}B). The spectra were referenced relative to residual protio solvent resonances. Elemental analyses were performed at Elemental Microanalysis Ltd., Okehampton, Devon, UK. Solvents (toluene, hexane) were dried by passage through a commercially available solvent purification system, under argon and stored in ampoules over 4 Å molecular sieves. C_6D_6 was purchased from Sigma-Aldrich, dried over a potassium mirror before vacuum distilling and storing under argon over molecular sieves. $CDCl_3$ was purchased from Sigma-Aldrich, dried by stirring with CaH_2 overnight before vacuum distilling and storing under argon over molecular sieves. $MesNH_2$ and $\{CH_2SiMe_2Cl\}_2$ were purchased from Sigma-Aldrich and distilled prior to use and compound **3** was prepared by a literature procedure.[1] All other reagents were purchased from Sigma-Aldrich and used without further purification.

Synthetic procedures

Synthesis of $\{SiN^{Mes}\}H_2$ (**8**)

A solution of *n*-BuLi in hexane (42.5 mL of a 2.5M solution, 0.107 mol) was added dropwise to a pre-cooled solution of $MesNH_2$ (14.4 g, 15 mL, 0.107 mol) in Et_2O (60 mL) at 0 °C. The resulting colorless suspension was stirred at room temperature for 1.5 hours followed by the dropwise addition of a solution of $\{CH_2SiMe_2Cl\}_2$ (11.5 g, 0.0535 mol) in Et_2O (30 mL) at 0 °C. The resultant suspension was stirred for 12 hours at room temperature, allowed to settle for 3 hours and filtered to give a clear colorless solution. Removal of the volatiles *in vacuo* followed by extraction into hexane (100 mL) gave a colorless suspension. Filtration of the solution followed by removal of the volatiles *in vacuo* provided **8** as a colorless waxy solid. Yield: 21.2 g, 96 %. ^{1}H NMR (500 MHz, C_6D_6): δ 6.83 (s, 4H, C_6H_2), 2.20 (s, 12H, *o-Me*), 2.19 (s, 6H, *p-Me*), 2.03 (s, 2H, N*H*), 0.54 (s, 4H, SiCH_2), 0.10 (s, 12H, SiMe_2). ^{13}C{^{1}H} NMR (125 MHz, C_6D_6): δ 141.0, 132.0, 131.0, 129.4 (C_6H_2), 20.8 (*p-Me*), 19.9 (*o-Me*), 9.9 (Si*C*H_2), –1.1 (SiMe_2).

Figure S1. ^{1}H NMR Spectrum (500 MHz, 298K, C_6D_6) of {SiN^{Mes}}H_2 (**8,** * = hexane).

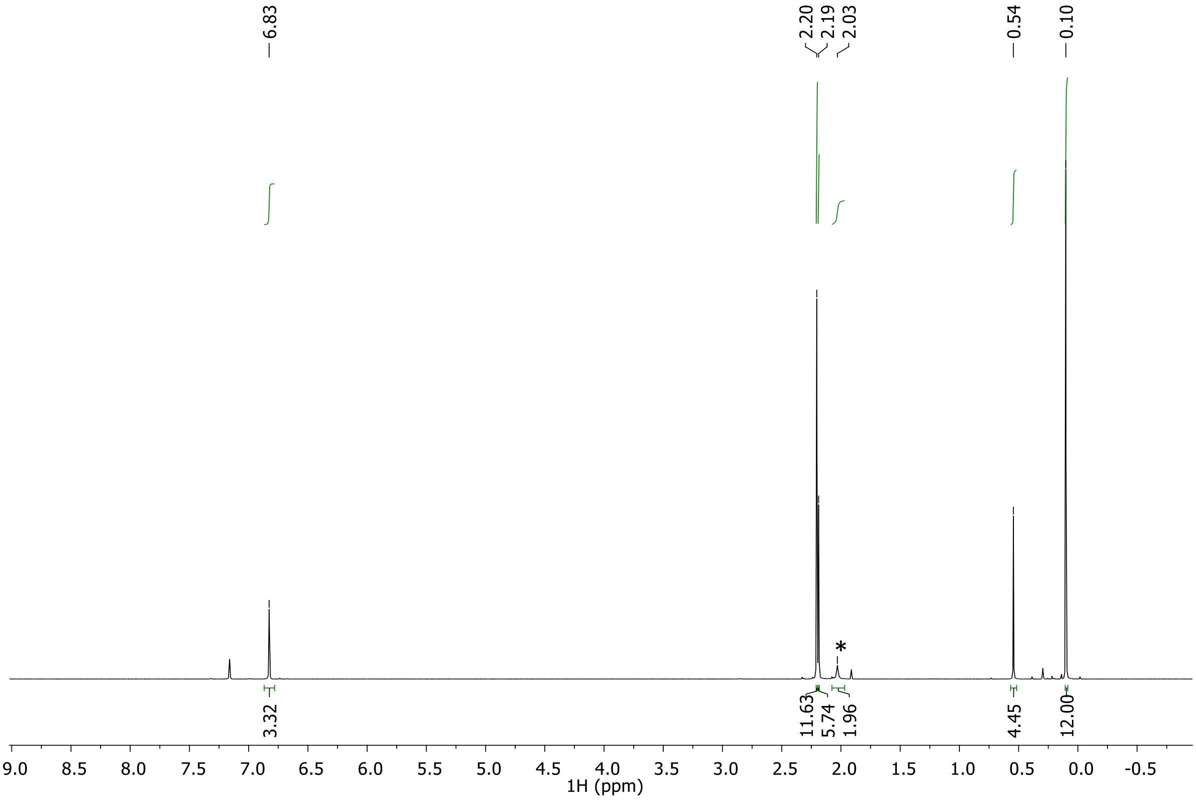

Figure S2. ^{13}C{^{1}H} NMR Spectrum (126MHz, 298K, C_6D_6) of {SiN^{Mes}}H_2 (**8**).

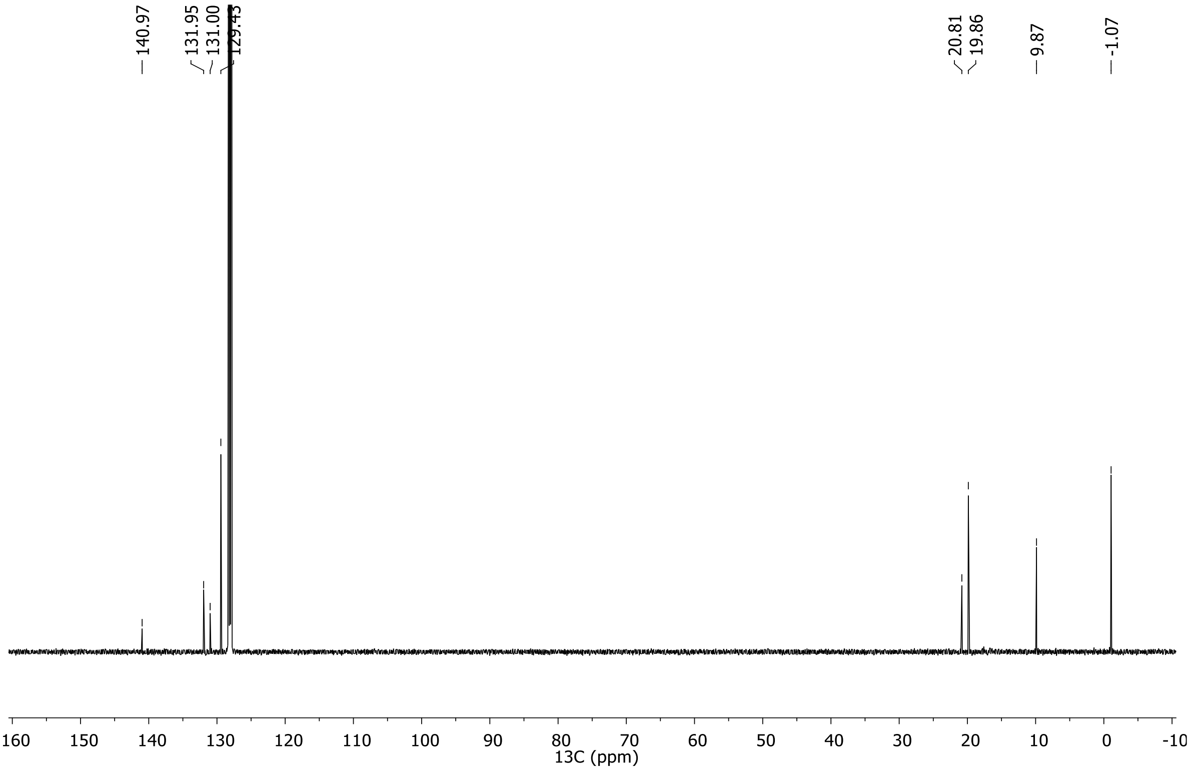

Synthesis of [SiN^{Mes}}AlMe] (**9**)

A solution of $AlMe_3$ in hexane (7.4 mL of a 2 M solution, 15.6 mmol) was added dropwise to a stirring solution of **8** (6.0 g, 14.8 mmol) in toluene (30 mL) at 0 °C. Upon addition, the solution bubbled and was stirred for 48 hours at room temperature under a slow flow of argon, followed by warming to 60 °C and stirring for 12 hours. The resulting colorless suspension was allowed to cool to room temperature and the volatile components were removed *in vacuo* to give **9** as a colorless waxy solid. Yield: 6.45 g, 97%. ^{1}H NMR (500 MHz, C_6D_6): δ 6.82 (s, 4H, C_6H_2), 2.28 (s, 12H, *o-Me*), 2.14 (s, 6H, *p-Me*), 1.05 (s, 4H, $SiCH_2$), 0.14 (s, 12H, $SiMe_2$), –1.11 (s, 3H, Al*Me*). ^{13}C{^{1}H} NMR (125 MHz, C_6D_6): δ 143.4, 135.1, 132.2, 129.7 (C_6H_2), 21.1 (*p-Me*), 20.8 (*o-Me*), 12.7 (Si*C*H_2), 0.7 (Si*Me*$_2$). *^{13}C NMR resonance not observed for Al*Me*.

Figure S3. ^{1}H NMR Spectrum (500 MHz, 298K, C_6D_6) of [{SiN^{Mes}}AlMe] (**9**).

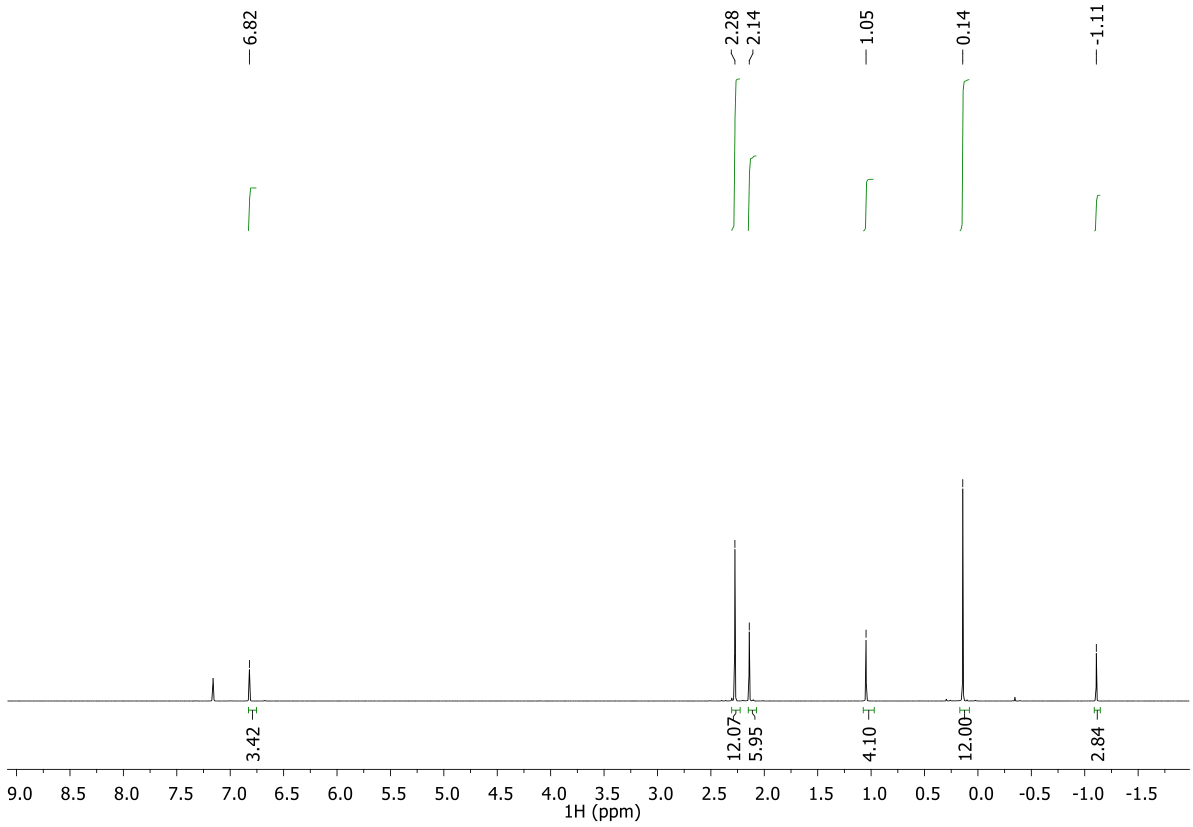

Figure S4. $^{13}C\{^1H\}$ NMR Spectrum (126 MHz, 298K, C_6D_6) of [{SiN^{Mes}}AlMe] (**9**)

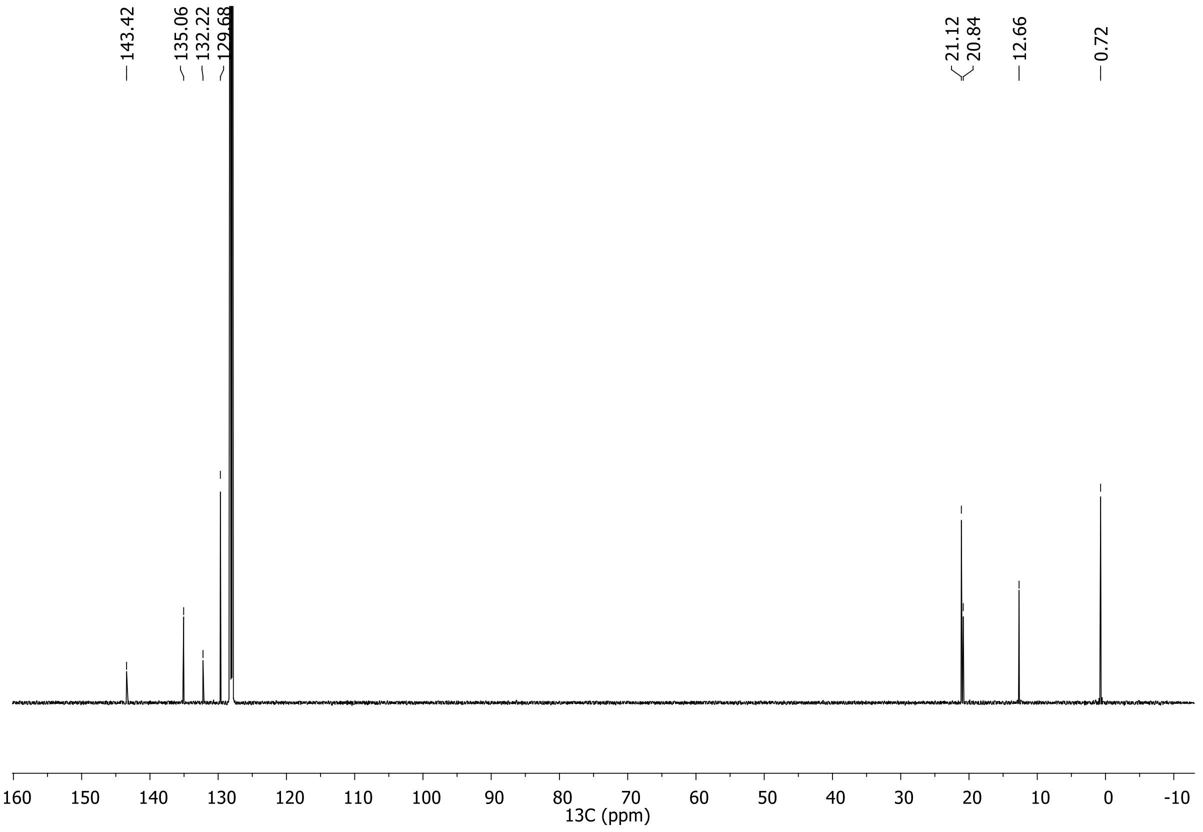

Synthesis of [{SiN^{Mes}}AlI] (**10**)

A solution of iodine (2.1 g, 8.27 mmol) in toluene (50 mL) was added dropwise to a stirring solution of **9** (3.5 g, 8.26 mmol) in toluene (40 mL) at 80 °C. The solution was refluxed for 4 days under a slow flow of argon, after which time the initial red color was lost and the solution became pale orange. The solution was allowed to cool to room temperature and the volatile components were removed *in vacuo* to give a waxy solid. Extraction into hexane and filtration gave a clear orange solution. Concentration of the orange solution followed by storage at –30 °C gave **10** as colorless crystals. Yield 2.85 g, 61%. ^1H NMR (500 MHz, C_6D_6): δ 6.83 (s, 4H, C_6H_2), 2.31 (s, 12H, *o-Me*), 2.12 (s, 6H, *p-Me*), 0.98 (s, 4H, $SiCH_2$), 0.11 (s, 12H, $SiMe_2$). $^{13}C\{^1H\}$ NMR (125 MHz, C_6D_6): δ 141.8, 135.3, 133.2, 129.9 (C_6H_2), 21.7 (*p-Me*), 20.9 (*o-Me*), 12.1 ($SiCH_2$), 0.7 ($SiMe_2$).

Figure S5. ^{1}H NMR Spectrum (500MHz, 298K, C_6D_6) of [{SiN^{Mes}}AlI] (**10**, * = hexane).

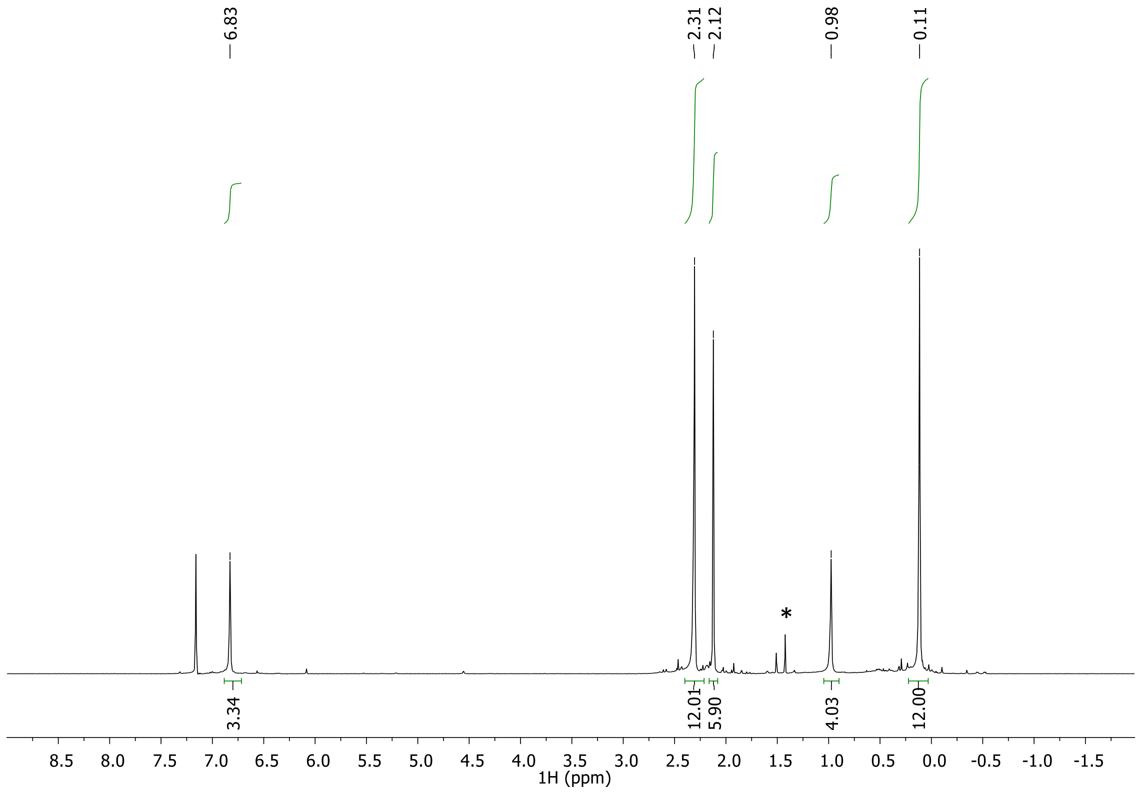

Figure S6. ^{13}C{^{1}H} NMR (126 MHz, 298K, C_6D_6) Spectrum of [{SiN^{Mes}}AlI] (**10**).

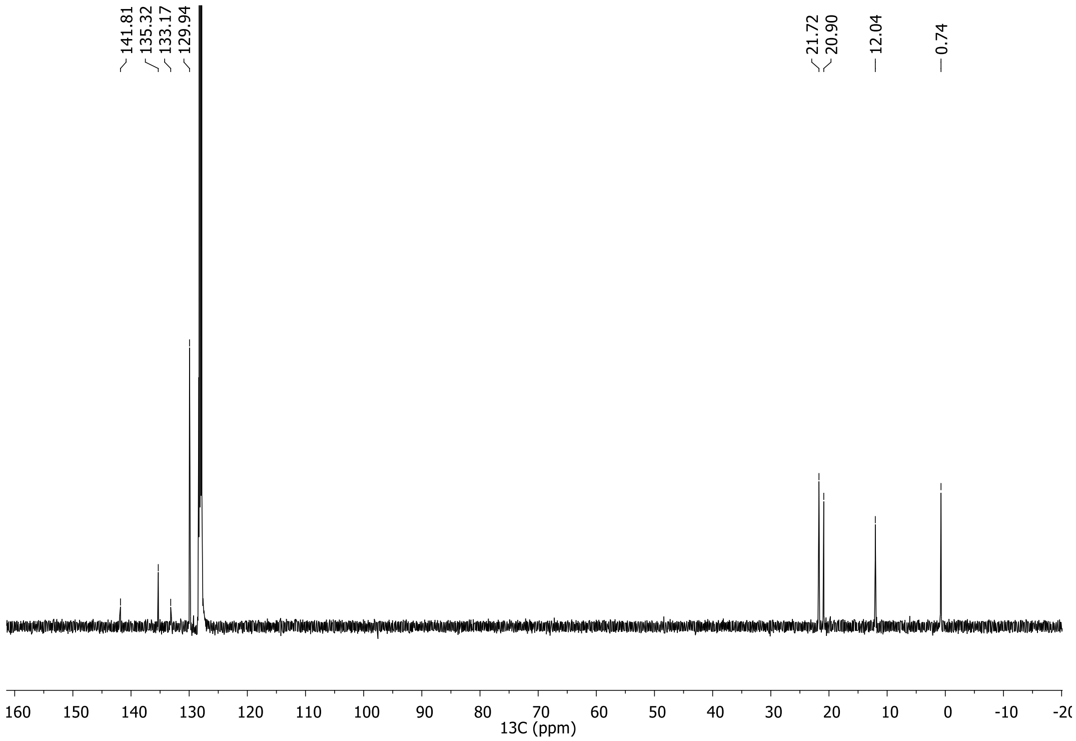

Synthesis of [{SiN^{Mes}}Al]$_2$ (**11**)

A solution of **10** (1.52 g, 2.70 mmol) in hexane (80 mL) was stirred on mirrored K (0.11 g, 2.9 mmol) for 3 days at room temperature resulting in the gradual color change from colorless to pale yellow and the formation of a grey precipitate. The solution was filtered through a cannula filter, concentrated to *ca.* 40 mL and stored at –30 °C for 24 hours to give **11** as colorless plates. Yield: 0.86 g, 73%. ^{1}H NMR (500 MHz, C_6D_6): δ 6.79 (s, 4H, C_6H_2), 2.26 (s, 6H, *p-Me*), 2.05 (s, 12H, *o-Me*), 0.86 (s, 4H, SiCH_2), 0.01 (s, 12H, SiMe_2). ^{13}C{^{1}H} NMR (125 MHz, C_6D_6): δ 143.6, 136.1, 131.9, 129.9 (C_6H$_2$), 22.6 (*p-Me*), 20.9 (*o-Me*), 14.2 (Si*C*H$_2$), 1.5 (Si*Me*$_2$).

Figure S7. ^{1}H NMR Spectrum (500MHz, 298K, C_6D_6) of [{SiN^{Mes}}Al]$_2$ (**11**). (**12**)

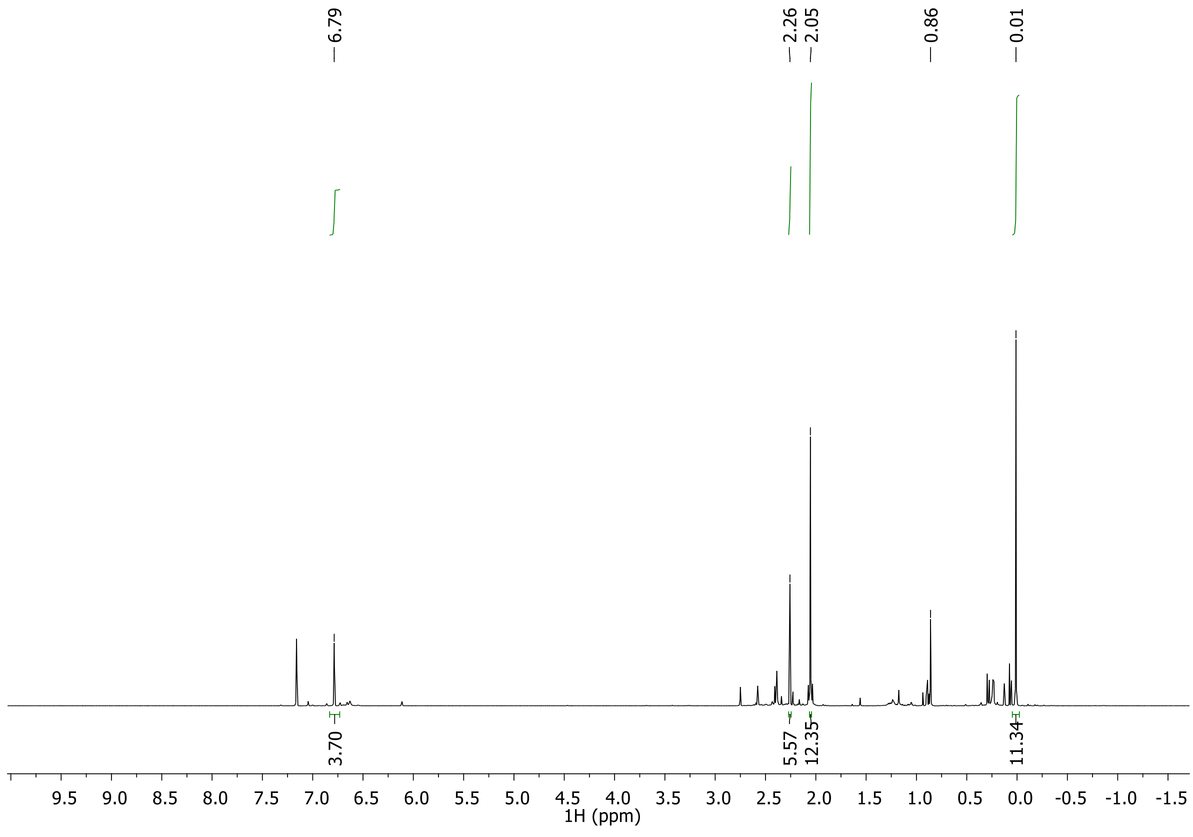

Figure S8. $^{13}C\{^{1}H\}$ NMR Spectrum (126MHz, 298K, C_6D_6) of $[\{SiN^{Mes}\}Al]_2$ (**11**)

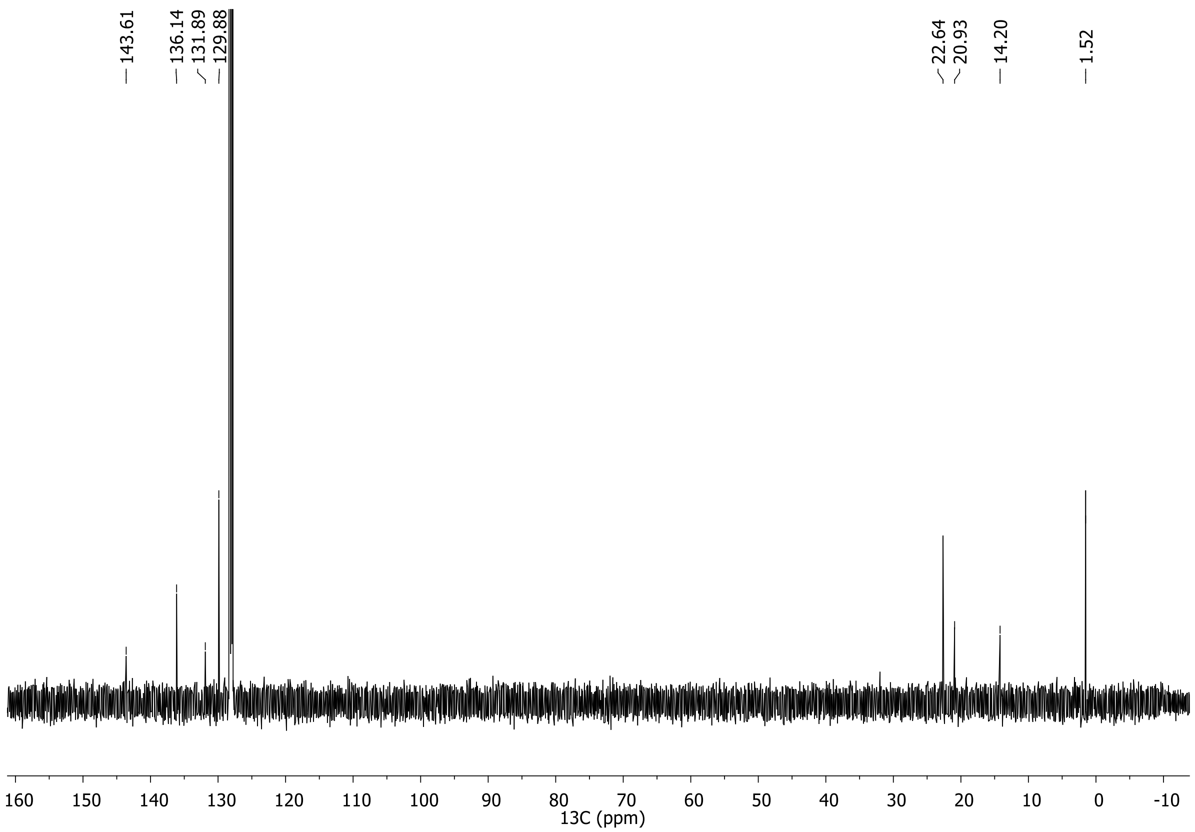

Synthesis of [{SiN^{Mes}Al}K]$_2$ (**12**)

Method 1:

A solution of **10** (4.68 g, 7.24 mmol) in hexane (30 mL) was stirred on mirrored K (0.85 g, 21.7 mmol) for 6 days at room temperature resulting in the gradual color change from colorless to yellow and the formation of a grey precipitate. The resulting dark yellow solution was filtered through a cannula filter, concentrated to *ca.* 15 mL and stored at −18 °C for 24 hours resulting in precipitation of a small amount of colorless solid identified as **11** by ^{1}H NMR spectroscopy. The suspension was filtered and the volatiles were removed in vacuo to give **12** as a yellow powder. Although bulk samples of **12** were invariably contaminated with variable quantities of **11**, X-ray quality crystals were grown from a concentrated Et_2O solution at –30 °C.

Method 2:

A solution of **11** (0.95 g, 1.08 mmol) in hexane (50 mL) on mirrored K (0.085g, 2.17 mmol) for 5 days at room temperature resulting in the gradual color change from colorless to yellow. Concentration of the solution and storage at −18 °C gave yellow crystals of **12** after 24 hours. Although bulk samples **12** prepared by this method were invariably contaminated with variable quantities of **11**, NMR data could be assigned with a reasonable level of confidence by discounting resonances assigned to compound **11**. ^{1}H NMR (500 MHz, C_6D_6): δ 6.63 (s, 4H, C_6*H_2*), 2.37 (s, 12H, *o-Me*), 2.32-2.29 (m, 6H, *p-Me*), 1.17 (s, 4H, SiC*H_2*), 0.23 (s, 12H, Si*Me_2*). ^{13}C{^{1}H} NMR (125 MHz, C_6D_6): δ 150.4, 131.5, 129.6, 128.7(*C*$_6$H$_2$), 20.9 (*p-Me*), 20.2 (*o-Me*), 11.7 (Si*C*H$_2$), 2.7 (Si*Me_2*).

Figure S9. ^{1}H NMR Spectrum (500 MHz, 298 K, C_6D_6) of [{SiN^{Mes}}AlK]$_2$ (**12**) *peaks correspond with [{SiN^{Mes}}Al]$_2$ (**11**).

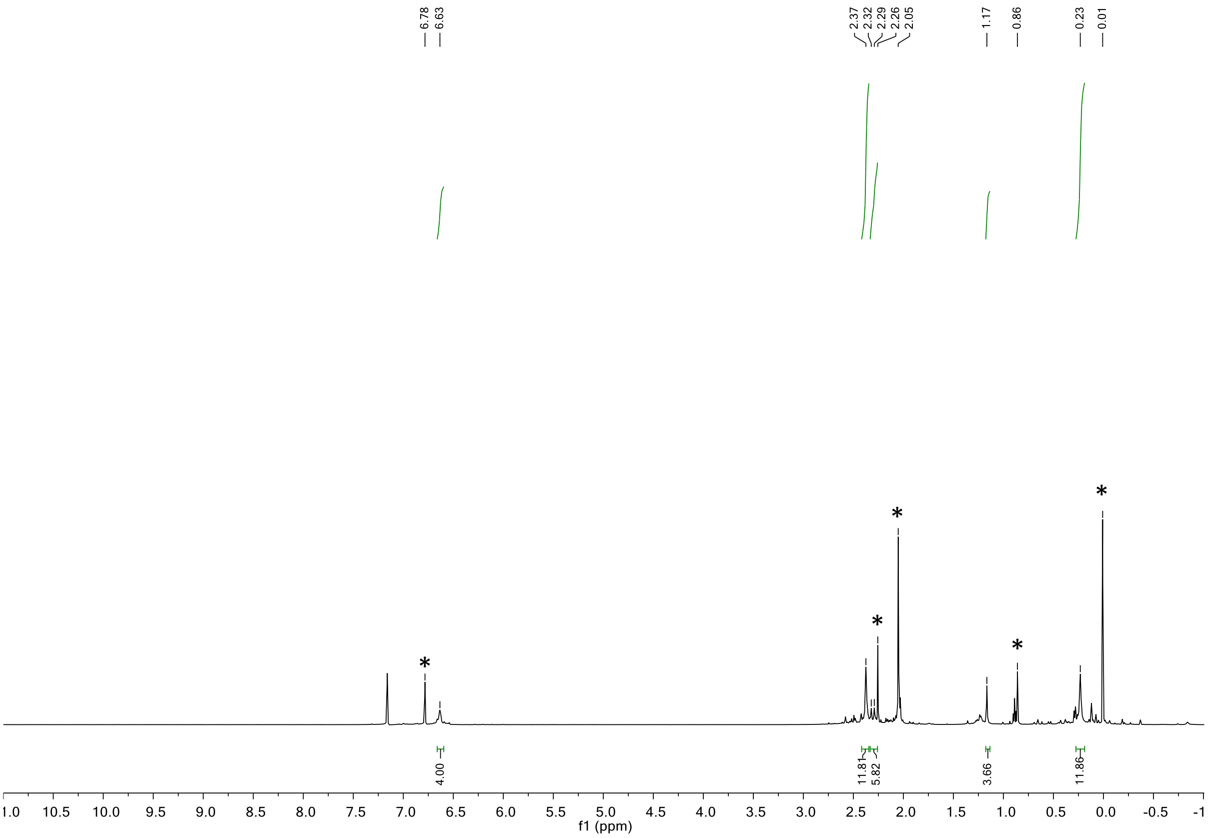

Figure S10. $^{13}C\{^{1}H\}$ NMR Spectrum (126 MHz, 298 K, C_6D_6) of [{SiN^{Mes}}AlK]$_2$ (**12**).

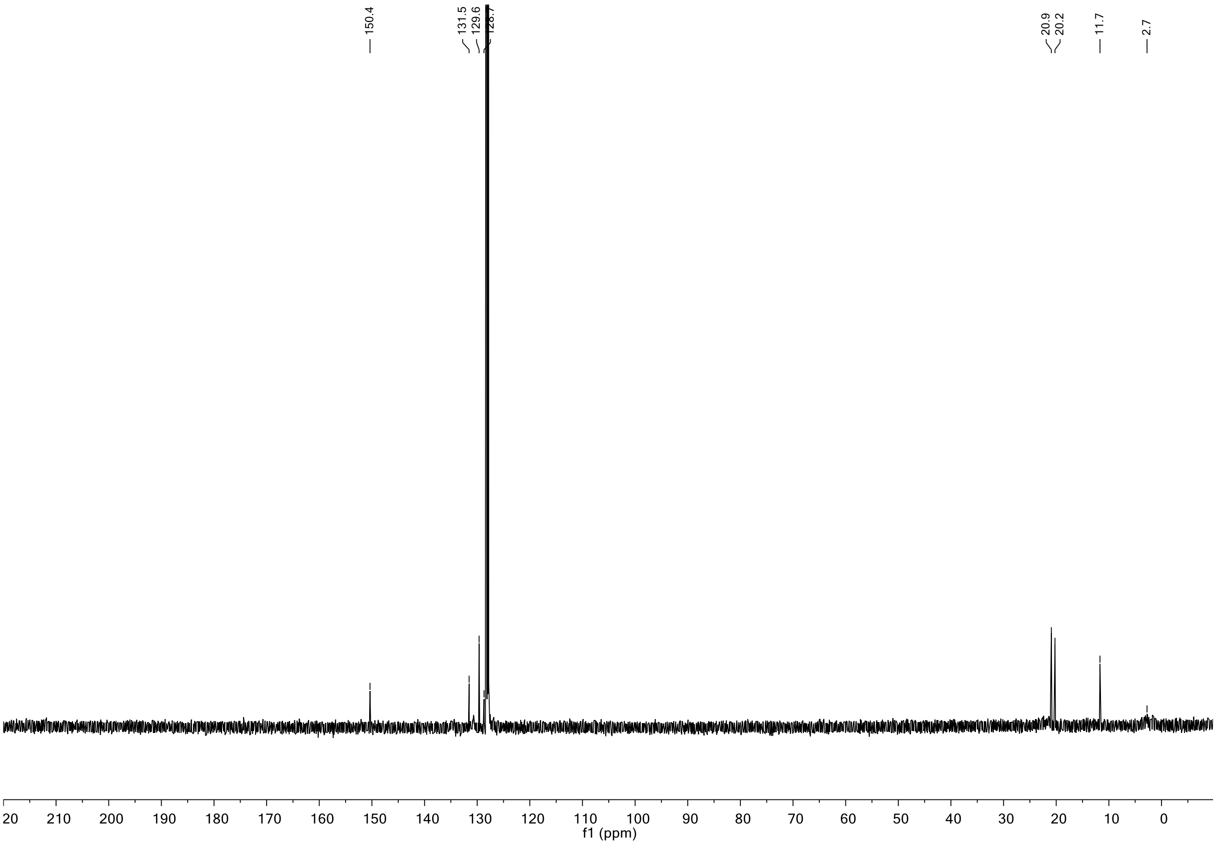

Isolation of [{SiN^{Dipp}}Al]$_2$ (**13**)

Compound **3** (28 mg, 0.025 mmol) was dissolved in 0.4 mL of C_6D_6 and 7,7,8,8-tetracyanoquinodimethane (TCNQ) (10.2mg, 0.05mmol) was added to the bright yellow solution. A blue precipitate was observed in the reaction mixture and the solution became pale yellow in color. The blue solid was removed by filtration and, while a bulk sample could not be isolated, compound **13** was identified by X-ray diffraction analysis performed on a colorless crystal selected from the mix of crystals obtained by slow evaporation of the benzene solution of the crude reaction mixture.

Synthesis of [{SiN^{Dipp}}AlH(CH_2Ph)]K (**14**)

Compound **3** (56 mg, 0.05 mmol) was dissolved in 0.4 mL of d_8-toluene. The solution was kept at 40 ^{o}C or 60 ^{o}C overnight and no significant change was observed in its ^{1}H NMR spectrum. The solution was then kept at 110 ^{o}C, whereupon the bright yellow solution slowly changed to pale-yellow. The reaction mixture was then kept under vacuum to remove all volatiles and re-dissolved in d_8-THF to provide **14** for NMR characterization in effectively stoichiometric yield. Single crystals suitable for X-ray diffraction analysis were obtained by slow evaporation of a toluene solution at room temperature. No meaningful result could be obtained for elemental analysis after multiple attempts. ^{1}H NMR (500 MHz, 298K, d_8-THF) δ 6.99 - 6.92 (m, 2H, *p*-C_6H_3), 6.84 – 6.72 (m, 4H, *m*-C_6H_3), 4.17 (sept, *J* = 6.8 Hz, 2H, C*H*Me$_2$), 4.04 (sept, J = 6.8 Hz, 2H, C*H*Me$_2$), 1.30 (d, J = 6.8 Hz, 6H, CH*Me$_2$*), 1.19 (d, *J* = 6.8 Hz, 6H, CH*Me$_2$*), 1.07 (d, J = 6.8 Hz, 6H, CH*Me$_2$*), 0.95 – 0.87 (s, 4H, SiC*H$_2$*), 0.78 (d, *J* = 6.8 Hz, 6H, CH*Me$_2$*), –0.04 (s, 6H, Si*Me$_2$*), –0.05 (s, 6H, Si*Me$_2$*). ^{2}H NMR (77 MHz, d_8-THF) δ 7.25 ($H_{aromatic}$), 5.39 ($H_{benzylic}$); Al-*H* not observed. ^{13}C{^{1}H} NMR (126 MHz, 298K, d_8-THF) δ 151.6 (*i*-C_6H$_3$), 148.4 (*o*-C_6H$_3$), 147.5 (*o*-C_6H$_3$), 124.1 (*m*-C_6H$_3$), 123.4 (*m*-C_6H$_3$), 121.2 (*p*-C_6H$_3$), 27.9 (*C*HMe$_2$), 27.8 (*C*HMe$_2$), 26.9 (CH*Me$_2$*), 26.3(CH*Me$_2$*), 26.1 (CH*Me$_2$*), 26.0 (CH*Me$_2$*), 16.2 (Si*C*H$_2$), 1.9 (Si*Me*$_2$), 1.8 (Si*Me*$_2$). ^{13}C resonance correlated to benzylic moiety not observed.

Figure S11. ^{1}H NMR spectrum (500 MHz, 298K, d_8-THF) of [{SiN^{Dipp}}AlH(CH_2Ph)]K (**14**).

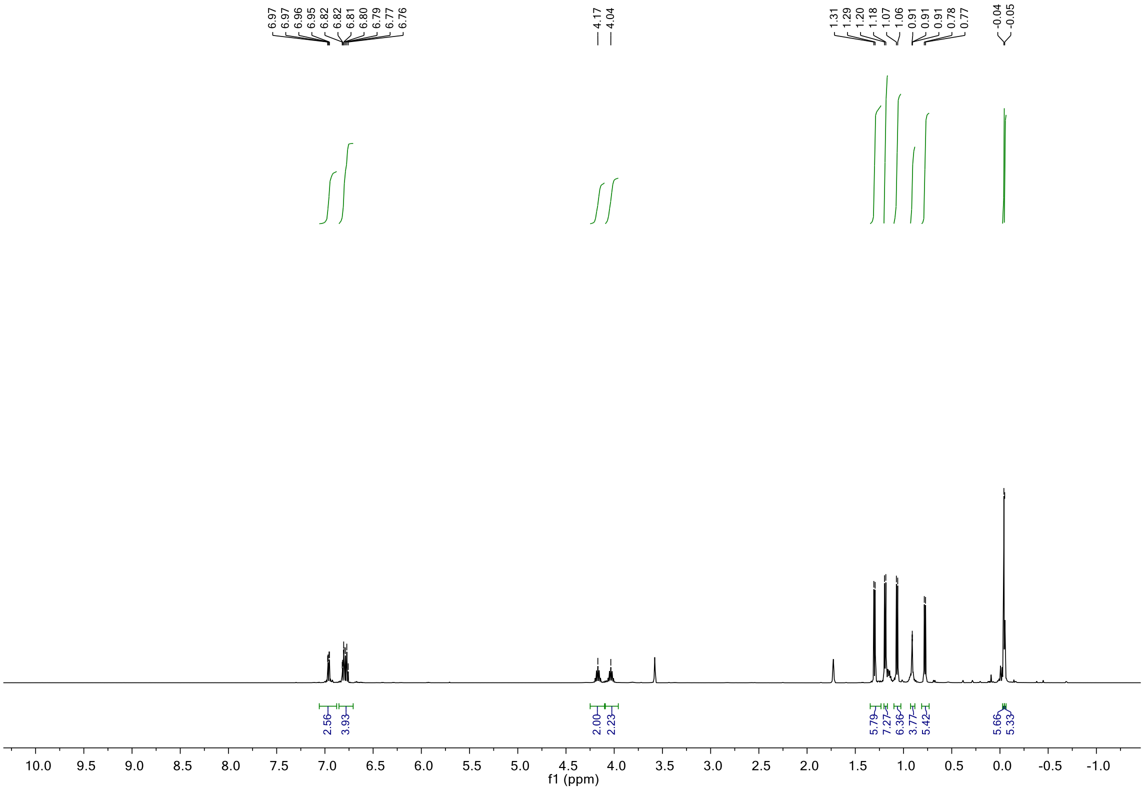

Figure S12. ^{2}H NMR spectrum (77 MHz, 298K, d_8-THF) of [{SiN^{Dipp}}AlH(CH_2Ph)]K (**14**).

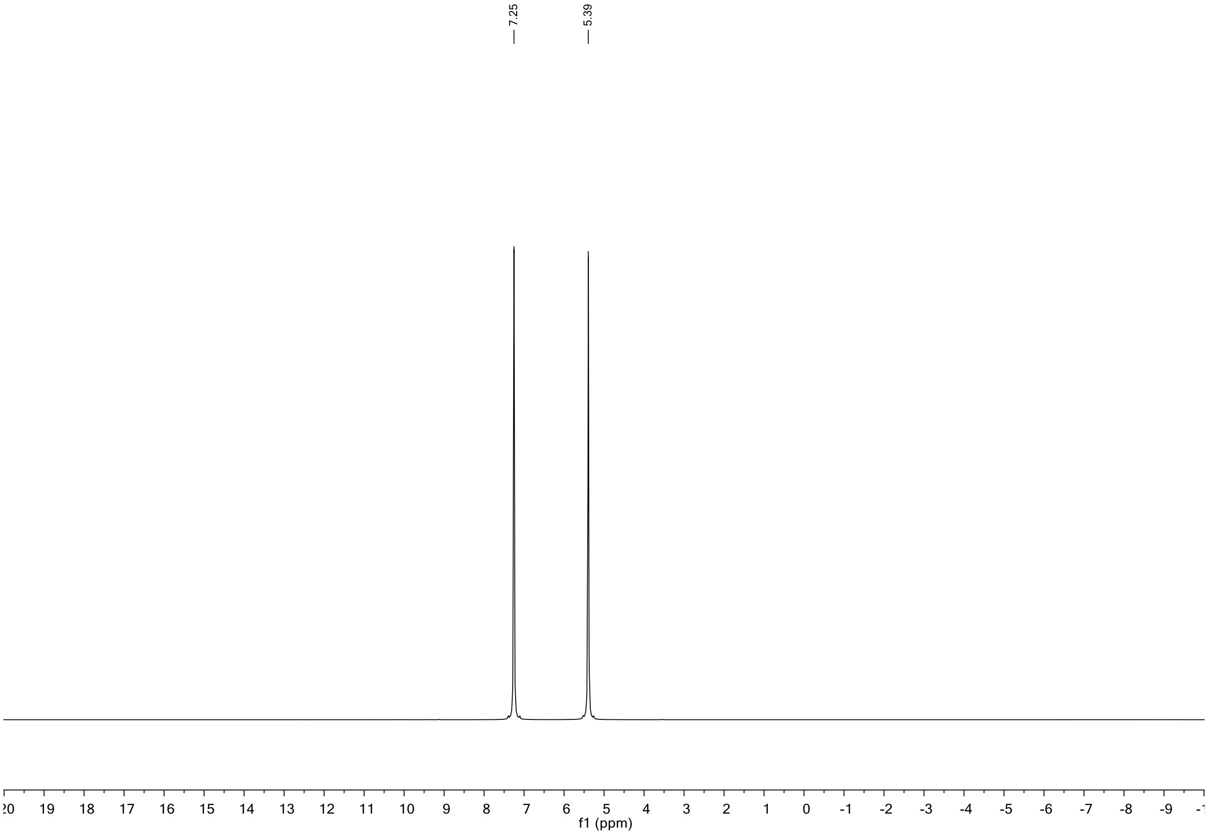

Figure S13. $^{13}C\{^{1}H\}$ NMR spectrum (126 MHz, 298K, d_8-THF) of $[\{SiN^{Dipp}\}AlH(CH_2Ph)]K$ (**14**).

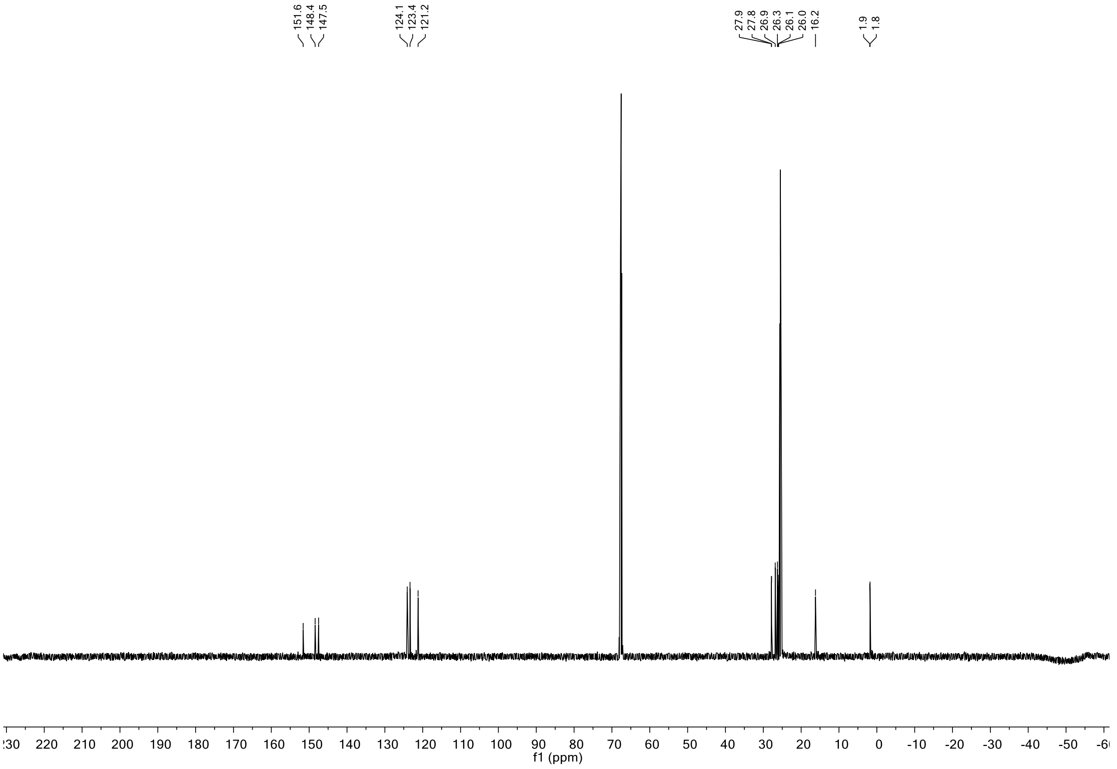

Synthesis of $K[Ph_3BAl\{N^{Dipp}Si\}]$ (**15**)

Compound **3** (23 mg, 0.0205 mmol) was dissolved in 0.4 mL of C_6D_6 and BPh_3 (10mg, 0.041 mmol) was added to the clear bright yellow solution. Compound **15** crystallized as a colorless solid from the reaction mixture. Yield 22 mg, 67%. The sample used to record NMR spectra was prepared by collecting the crystals from the reaction mixture, which were washed with hexane before being dissolved in deuterated chloroform. A single crystal suitable for X-ray diffraction analysis was obtained by slow evaporation of a saturated toluene solution at ambient temperature. Anal. Calcd. for $C_{55}H_{73}AlBN_2Si_2K$ [**15**.C_7H_8] (895.26); C,73.79; H,8.22; N, 3.13; Found, C, 73.83; H, 8.28; N, 2.78. ^{1}H NMR (500 MHz, $CDCl_3$) (With BPh_3 and solvent impurities): δ 7.66 – 7.62 (m, 6H, B*Ph*$_3$), 7.59 – 7.54 (m, 3H, *p*-B*Ph*$_3$), 7.48-7.45 (m, 6H, B*Ph*$_3$), 7.07 – 7.00 (m, 6H, C_6*H*$_3$), 3.46 – 3.29 (sept, *J* = 6.9 Hz, 4H, C*H*Me$_2$), 1.18 (d, *J* = 6.9 Hz, 24H, CH*Me*$_2$), 0.58 (s, 4H, SiC*H*$_2$), 0.09 (s, 12H, Si*Me*$_2$). $^{13}C\{^{1}H\}$ NMR (125 MHz, $CDCl_3$) δ 143.8, 143.6, 139.8 (quaternary carbons for NC_6H_3 and B*Ph*$_3$), 138.6, 131.2, 127.4, (B*Ph*$_3$) 123.0, 122.8 (N*C*$_6H_3$), 28.1 (*C*HMe$_2$), 23.6 (CH*Me*$_2$), 9.5 (Si*C*H$_2$), −1.75 (Si*Me*$_2$). No ^{11}B resonance correlated to the LAl-*B*Ph$_3$ could be observed.

Figure S14. ^{1}H NMR (500 MHz, 298K, $CDCl_3$) spectrum of K[$Ph_3BAl\{N^{Dipp}Si\}$] (**15**, * = hexane, † = toluene).

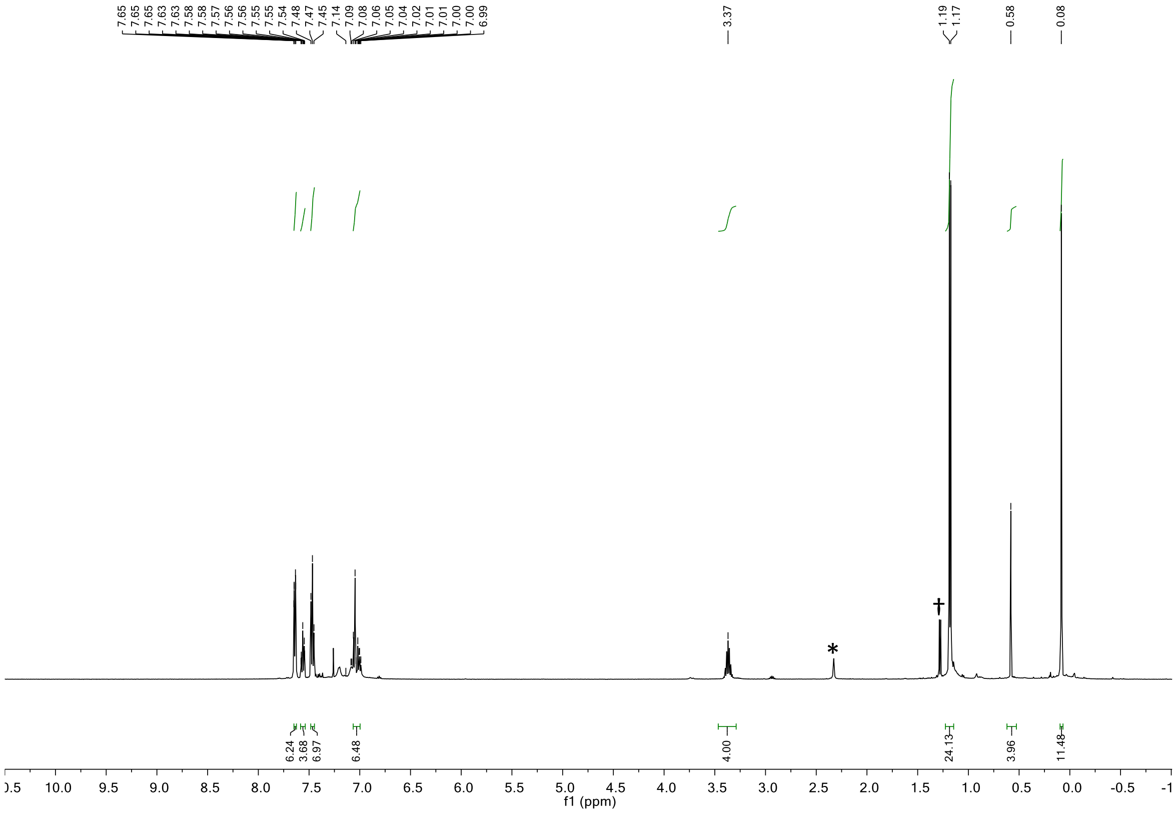

Figure S15. $^{13}C\{^{1}H\}$ NMR (126 MHz, 298K, $CDCl_3$) spectrum of K[$Ph_3BAl\{N^{Dipp}Si\}$] (**15**).

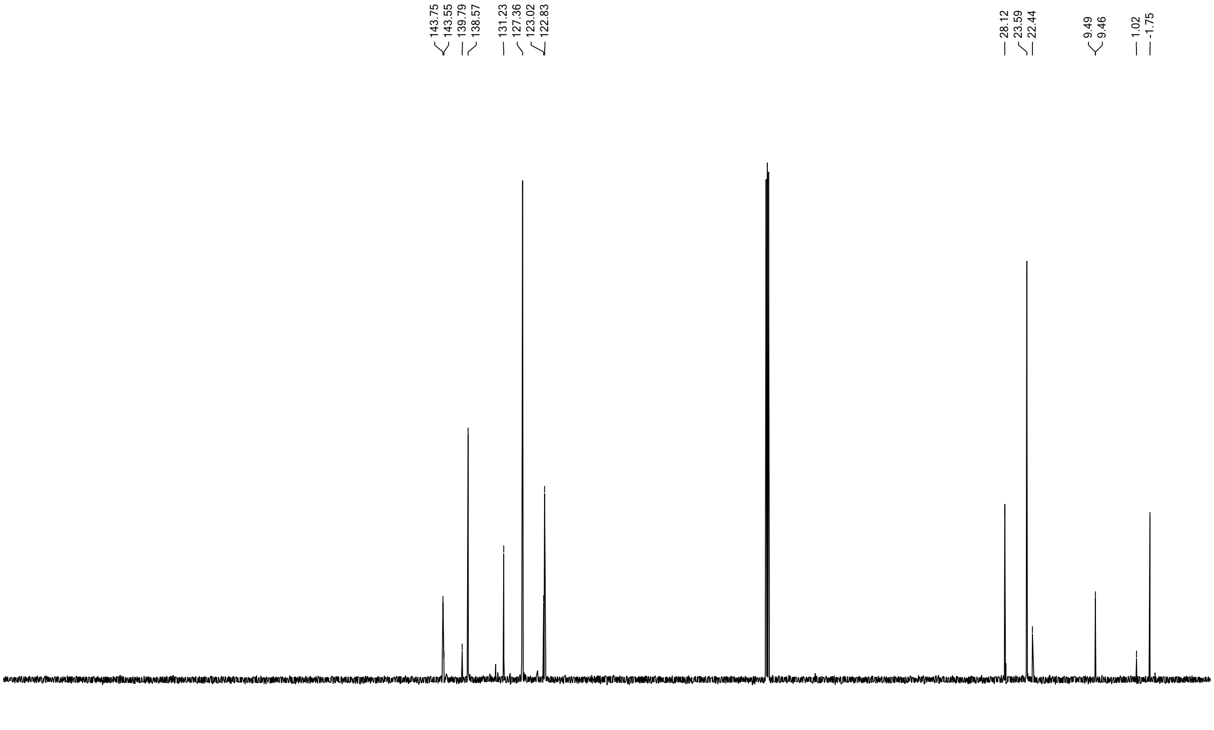

Figure S16. ^{1}H-^{1}H COSY NMR spectrum of K[Ph$_3$BAl{N^{Dipp}Si}] (**15**).

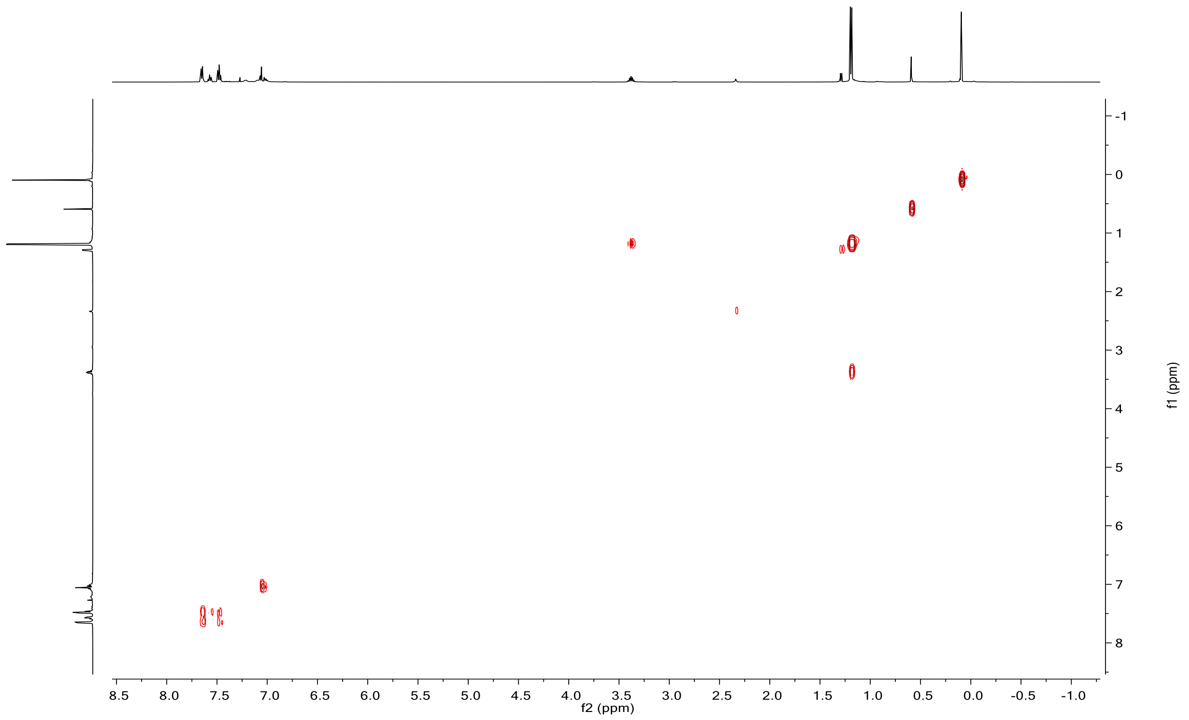

Figure S17. ^{1}H-^{13}C HSQC NMR spectrum of K[Ph$_3$BAl{N^{Dipp}Si}] (**15**).

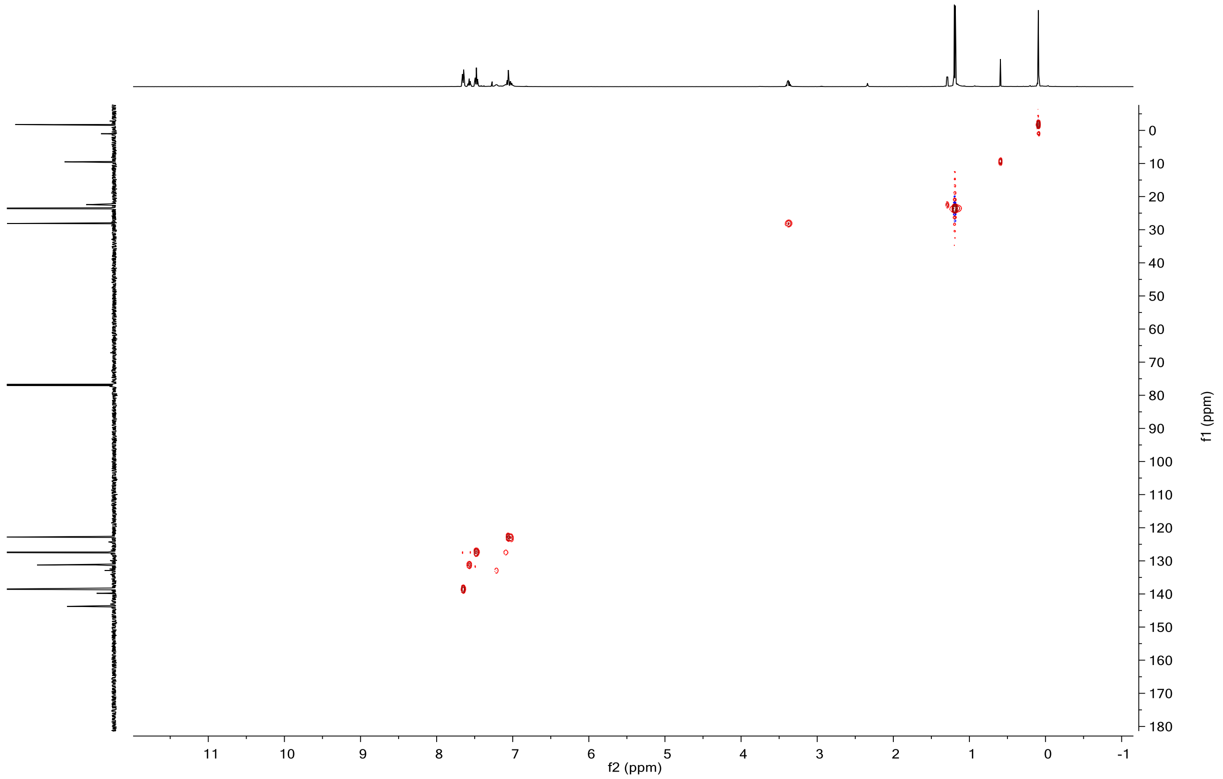

Figure S18. ^{11}B NMR spectrum (160MHz, 298K, $CDCl_3$) of $K[Ph_3BAl\{N^{Dipp}Si\}]$ (**15**).

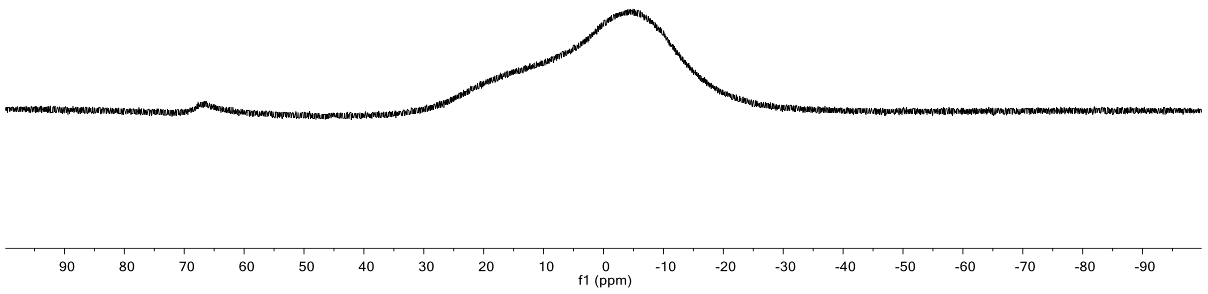

Reaction between **3** and MeOTf: Synthesis of [{SiN^{Dipp}}AlMe] (**16**)

In a Youngs NMR tube, potassium alumanyl **3** (26 mg, 0.012mmol) was dissolved in 0.4 mL of d_6-benzene before the addition of MeOTf (5 μL, 7.5 mg, 0.046 mmol). The reaction mixture became colorless with the instantaneous formation of a white precipitate. NMR spectra were recorded without further purification of the crude product, and the resonances were in good agreement with the previously reported data for [{SiN^{Dipp}}AlMe].[1] ^{1}H NMR (500 MHz, 298K, Benzene-d_6) δ 7.04 (m, 6H, C_6H_3), 3.75 (sept, J = 6.9 Hz, 4H, C*H*Me_2), 1.28 (d, J = 6.9 Hz, 12H, CHMe_2), 1.19 (d, J = 6.9 Hz, 12H, CHMe_2), 1.05 (s, 4H, SiCH_2), 0.17 (s, 12H, SiMe_2), -1.08 (s, 3H, Al*Me*). $^{13}C\{^{1}H\}$ NMR (125 MHz, 298K, benzene-d_6): δ 145.7, 143.3, 124.5, 123.9 (C_6H_3), 28.4, 24.9, 24.4 (CHMe_2 and *C*HMe_2), 13.4 (SiCH_2), 0.03 (SiMe_2). $^{13}C\{^{1}H\}$ NMR resonance for AlMe not observed.

Figure S19. ^{1}H NMR (500 MHz, 298K, C_6D_6) spectrum of [{SiN^{Dipp}}AlMe]. (**16**)

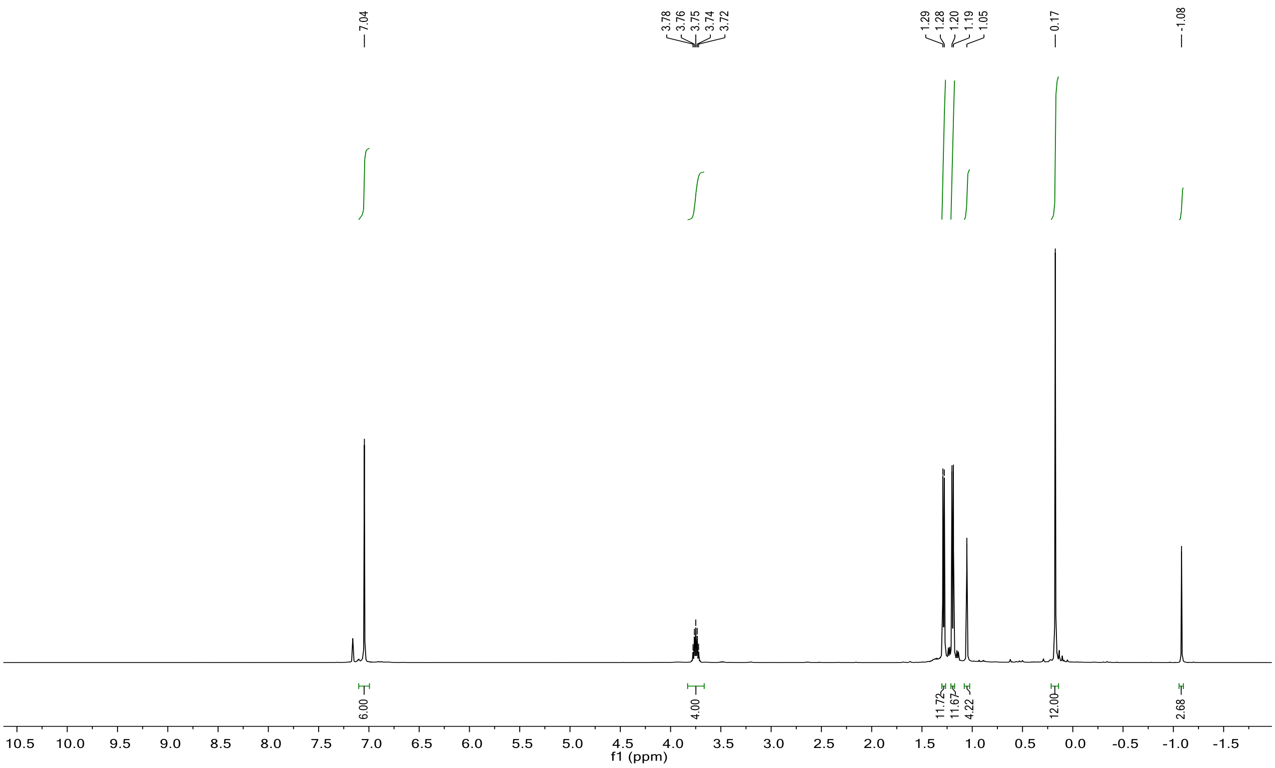

Figure S20. $^{13}C\{^{1}H\}$ NMR (126 MHz, 298K, C_6D_6) spectrum of [{SiN^{Dipp}}AlMe]. (**16**)

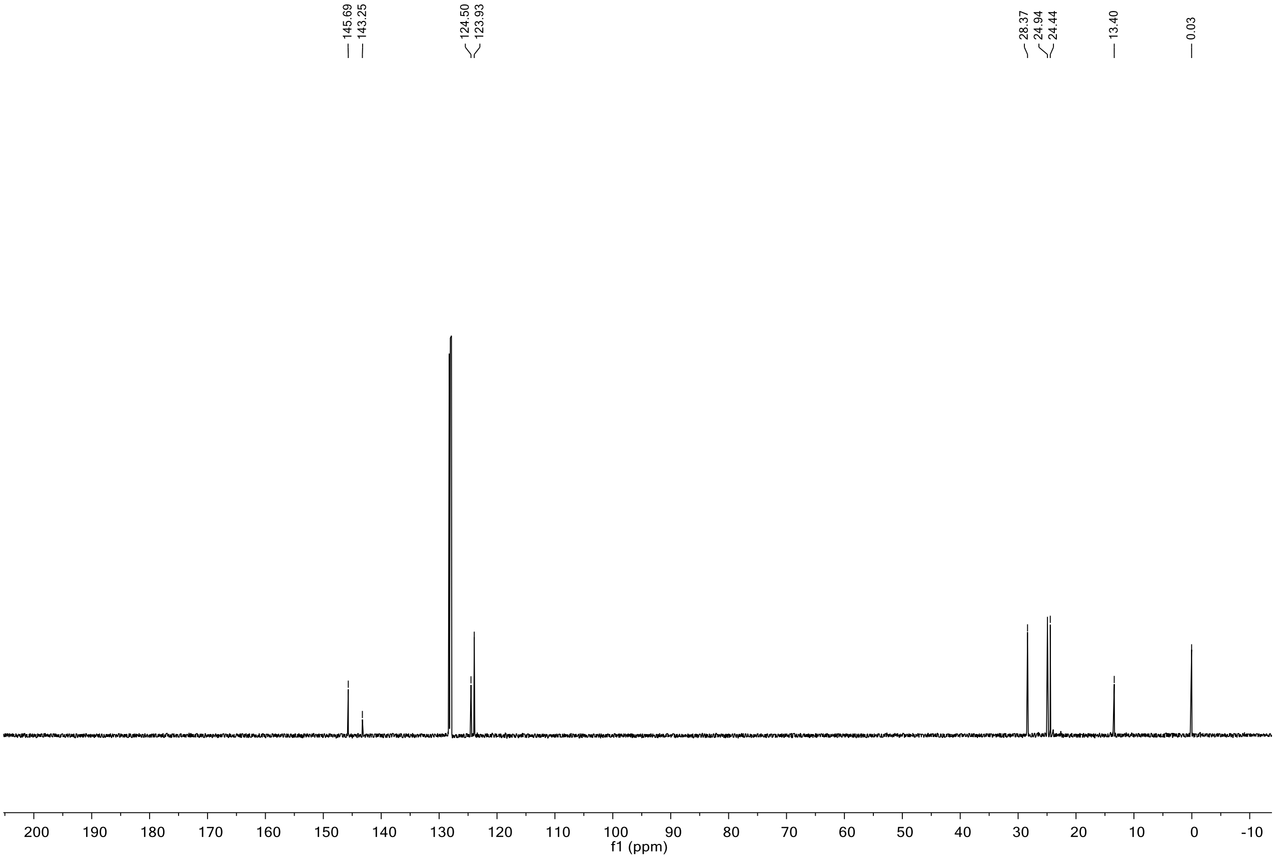

Synthesis of {SiN^{Dipp}}$AlCH(CH{=}CH)_2C{=}CPh_2$ (**17**)

Compound **3** (15mg, 0.0135mmol) was dissolved in 0.4 mL of d_8-toluene and $[CPh_3][B(C_6F_5)_4]$ (24.7mg, 0.027mmol) was then added to the bright yellow solution**.** The reaction mixture demonstrated a series of rapid color changes from bright yellow to green to bright red then to light yellow within 10 minutes. Some colorless crystals were then observed to form, the identity of which was confirmed by unit cell screening to be $K[B(C_6F_5)_4]$. The solution was then filtered, whereupon slow evaporation of the toluene solution at room temperature afforded colorless crystals of **17** suitable for X-ray diffraction analysis. Yield 11 mg, 53% No meaningful result was obtained for elemental analysis after multiple attempts. ^{1}H NMR (300 MHz, 298K, toluene-d_8) δ 7.18-7.15 (m, 1H, Ar*H*), 7.15-7.12 (m, 2H, Ar*H*), 7.09-7.06 (m, 2H, C=C-Ar*H*), 7.06-7.04 (m, 1H, Ar*H*), 6.99-6.98 (m, 4H, Ar*H*), 6.95-6.90 (m, 6H, Ar*H*), 5.99 (dd, J = 10.4, 2.2 Hz, 2H, Al-CH-C*H*=CH), 4.39 (dd, J = 10.4, 4.3 Hz, 2H, Al-CH-CH=C*H*), 3.70 (sept, J = 6.9 Hz, 2H, C*H*Me2), 2.85-2.82 (m, 1H, Al-C*H*-CH=CH), 1.28 (d, J = 6.9 Hz, 12H, CH*Me*$_2$), 1.25 (d, J = 6.9 Hz, 12H, CH*Me*$_2$), 0.99 (s, 4H, SiC*H*2), 0.13 (s, 12H, Si*Me*2). ^{13}C{^{1}H} NMR (75 MHz, 298K, toluene-d_8) δ 145.5 (C_{sp2}), 143.5 (C_{sp2}), 142.9 (C_{sp2}), 137.5 (C_{sp2}), 130.9 (C_{sp2}), 130.7 (C_{sp2}), 130.6 (C_{sp2}), 129.4 (C_{sp2}), 128.1 (C_{sp2}), 126.2 (C_{sp2}), 124.3 (C_{sp2}), 124.1 (C_{sp2}), 28.5 (*C*HMe$_2$), 4.9 (CH*Me*$_2$), 24.4 (CH*Me*$_2$), 13.5 (SiC*H*$_2$), –0.06 (Si*Me*$_2$). *Al*C*H not observed.

Figure S21. ^{1}H NMR spectrum (300MHz, 298K, d_8-toluene) of compound **17,** * = grease.

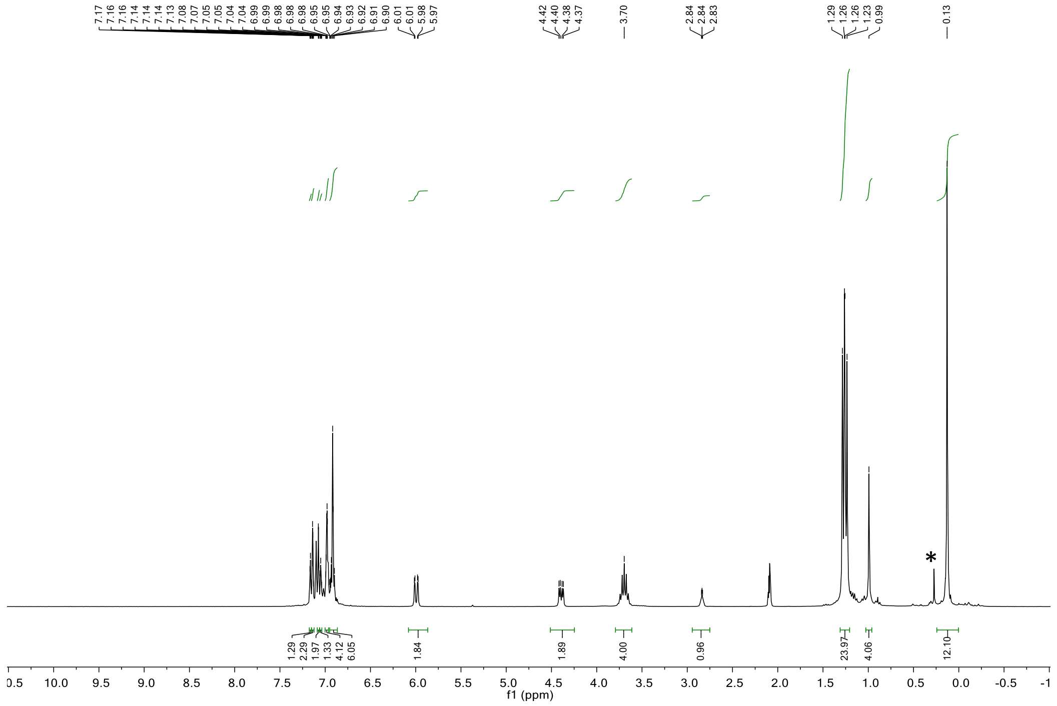

Figure S22. $^{13}C\{^{1}H\}$ NMR spectrum (75MHz, 298K, d_8-toluene) of compound **17**.

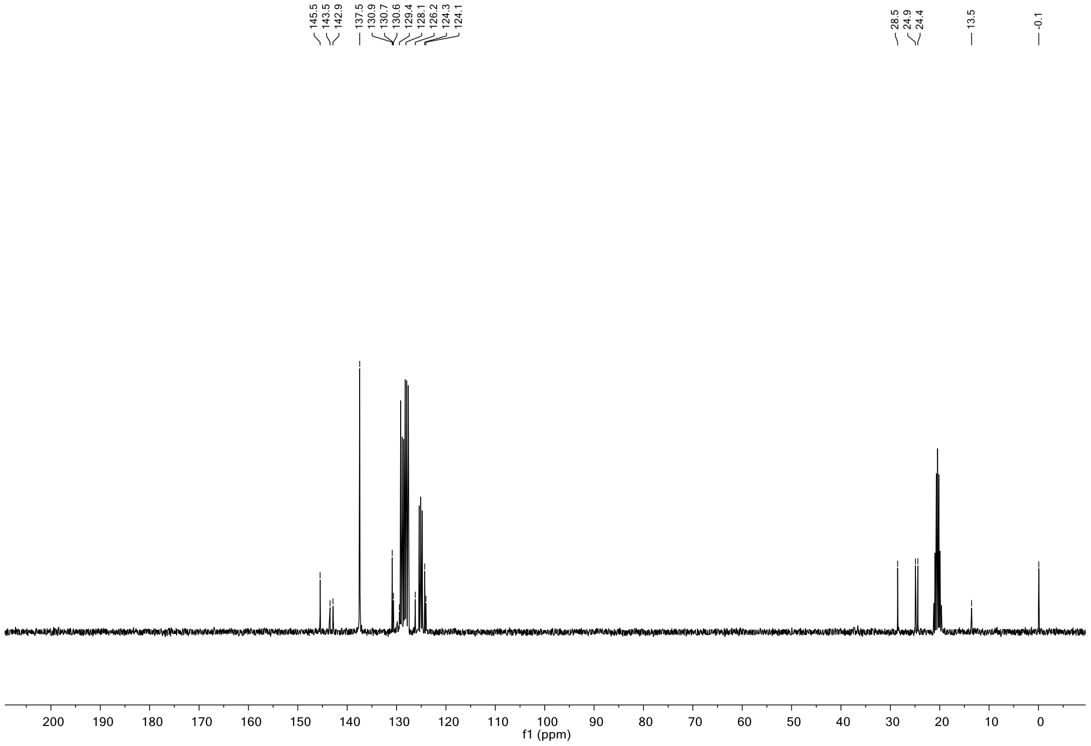

Synthesis of $[\{SiN^{Dipp}\}AlN^{iPr}C\text{-}N^{iPr}]K$ (**18**).

Compound **3** (28mg, 0.025mmol) was dissolved in 0.4 mL of C_6D_6 and $^{i}PrNCN^{i}Pr$ (6.3mg,7.8mL, 0.05 mmol) was added to the bright solution via pipette. The color of the solution slowly went from bright yellow to colorless, whilst some colorless crystals dropped out from the reaction mixture overnight. An X-ray quality single crystal was selected from the crystalline solids and the remaining crystalline material was collected and dried under vacuum to afford compound **18**. Yield 25 mg, 74%. The solid was then dissolved in d_8-THF to prepare a suitable NMR sample. No meaningful result was obtained for elemental analysis after several attempts. ^{1}H NMR (500 MHz, 298K, d_8-THF) δ 7.03 - 6.93 (m, 4H, *m*-C_6H_3), 6.86 - 6.75 (m, 2H, *p*-C_6H_3), 4.15 - 4.00 (m, 4H, C*H*Me_2), 3.18 - 3.08 (m, 1H, NC*H*Me_2), 2.98 - 2.90 (m, 1H, NC*H*Me_2), 1.28 - 1.18 (m, 24H, CH*Me_2*) 1.13 - 1.03 (m, 6H, NCH*Me_2*) 0.60 - 0.46 (m, 6H, NCH*Me_2*), 0.40 - 0.28 (br, 4H, SiC*H_2*) 0.15 - –0.05 (br, 12H, Si*Me_2*). $^{13}C\{^{1}H\}$ NMR (126 MHz, 298K, d_8-THF) δ 149.9 (*i-C_6*H_3), 147.4 (*o*-C_6H_3), 147.1 (*o*-*C*$_6H_3$), 123.5 (*m*-*C*$_6H_3$), 123.2 (*m*-C_6H_3), 122.2 (*p*-C_6H_3), 56.6 (N*C*HMe_2), 43.3(N*C*HMe_2), 28.4 (*C*HMe_2), 27.9 (NCH*Me_2*), 27.1 (CH*Me_2*), 26.6 (CH*Me_2*), 26.1 (CH*Me_2*), 26.0 (CH*Me_2*), 23.1 (NCH*Me_2*), 15.8 (Si*C*H_2), 2.1 (Si*Me_2*).

Figure S23. ^{1}H NMR spectrum (500 MHz, 298K, d_8-THF) of [{SiN^{Dipp}}AlN^{iPr}C-N^{iPr}]K (**18**).

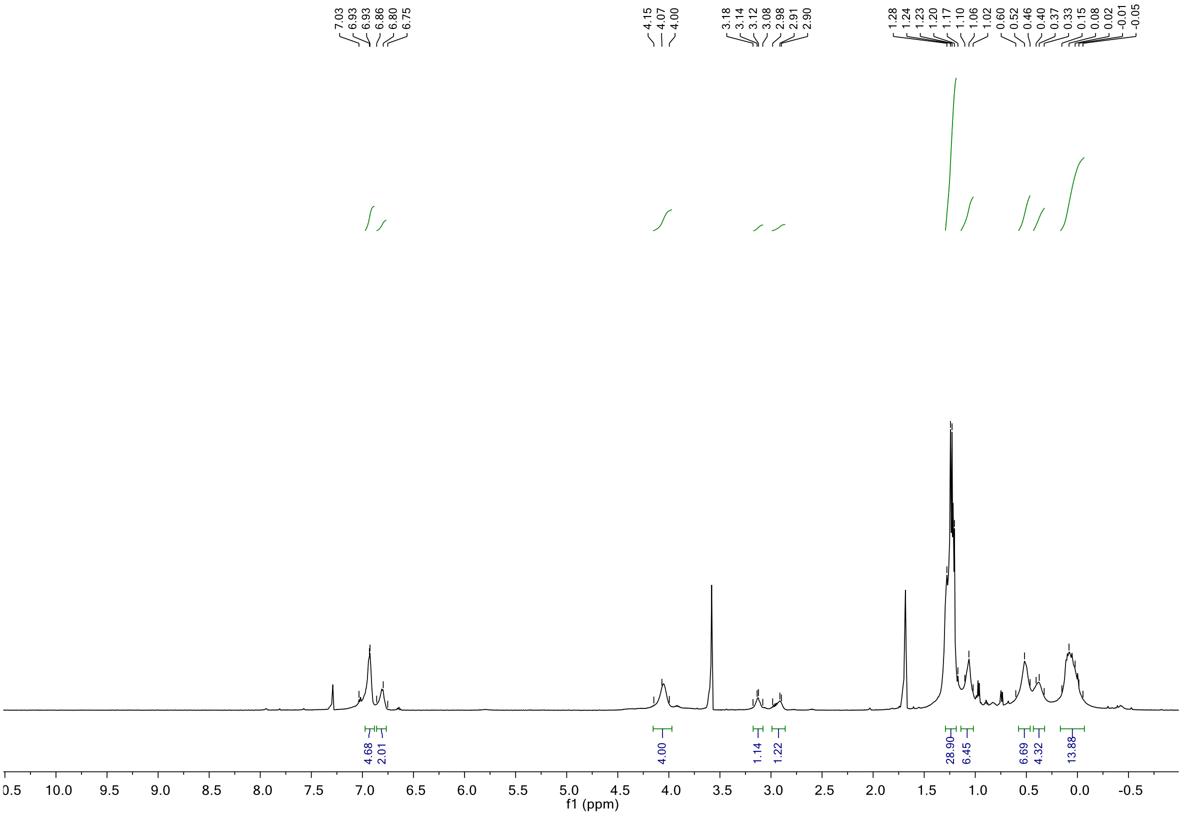

Figure S24. ^{13}C{^{1}H} NMR spectrum (126 MHz, 298K, d_8-THF) of [{SiN^{Dipp}}AlN^{iPr}C-N^{iPr}]K (**18**).

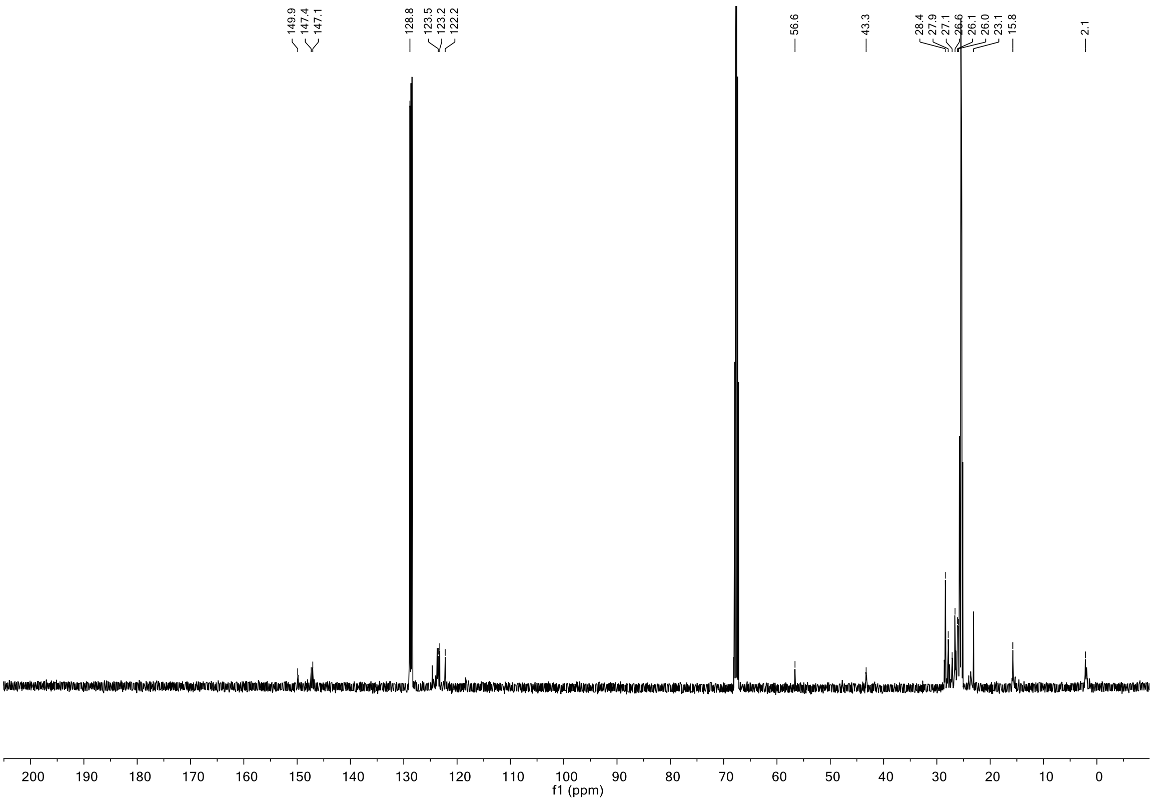

Synthesis of [{SiN^{Dipp}}AlN^{Cy}C-N^{Cy}]K (**19**).

Compound **3** (9.2mg, 0.008mmol) was dissolved in 0.4 mL of C_6D_6 and CyNCNCy (3.3mg, 0.016 mmol) was added to the bright solution. The color of the solution slowly went from bright yellow to colorless when the reaction mixture was kept at 40 ^{o}C for 2 hours. An X-ray quality single crystal was selected and the remaining crystalline solid was collected, washed with hexane and dried under vacuum to afford compound **19**. 1Yield 8.7 mg, 70%. No meaningful result could be obtained for elemental analysis after several attempts. ^{1}H NMR (500 MHz, 298K, d$_8$-THF) δ 6.88-6.85 (m, 4H, *m*-C_6H_3), 6.75-6.72 (m, 2H, *p*-C_6H_3), 4.07-3.91 (m, 4H, C*H*Me$_2$), 2.69-2.63 (m, 1H, N-C*H* on cyclohexyl), 2.37-2.31 (m, 1H, N-C*H* on cyclohexyl), 1.37-1.28 (m, 10H, *H*-cyclohexyl), 1.25-1.19 (m, 12H, CH*Me$_2$*), 1.17-1.13 (m, 12H, CH*Me$_2$*, 1.07- 0.94 (m, 10H, *H*-cyclohexyl), 0.53- 0.25 (br, 4H, SiC*H$_2$*) , 0.04 - –0.15 (br, 12H, Si*Me$_2$*). ^{13}C{^{1}H} NMR (126 MHz, d$_8$-THF) δ 149.9 (*i*-*C*$_6$H$_3$), 147.0 (*o*-*C*$_6$H$_3$), 123.6 (*m*-*C*$_6$H$_3$), 122.1 (*p*-*C*$_6$H$_3$), 65.9 (N*C*$_{cyclohexyl}$), 52.7 (N*C*$_{cyclohexyl}$), 38.4 (*C*$_{cyclohexyl}$), 32.7 (*C*$_{cyclohexyl}$), 28.5 (*C*HMe$_2$), 27.8 (*C*$_{cyclohexyl}$), 27.4 (*C*$_{cyclohexyl}$), 26.8 (CH*Me$_2$*) , 26.5 (*C*$_{cyclohexyl}$), 26.3 (CH*Me$_2$*), 23.7(*C*$_{cyclohexyl}$), 15.7 (Si*C*H$_2$), 2.2 (Si*Me$_2$*). Al*C*N$_2$ not observed.

Figure S25. ^{1}H NMR spectrum (500 MHz, 298K, d$_8$-THF) of [{SiN^{Dipp}}AlN^{Cy}C-N^{Cy}]K (**19**)

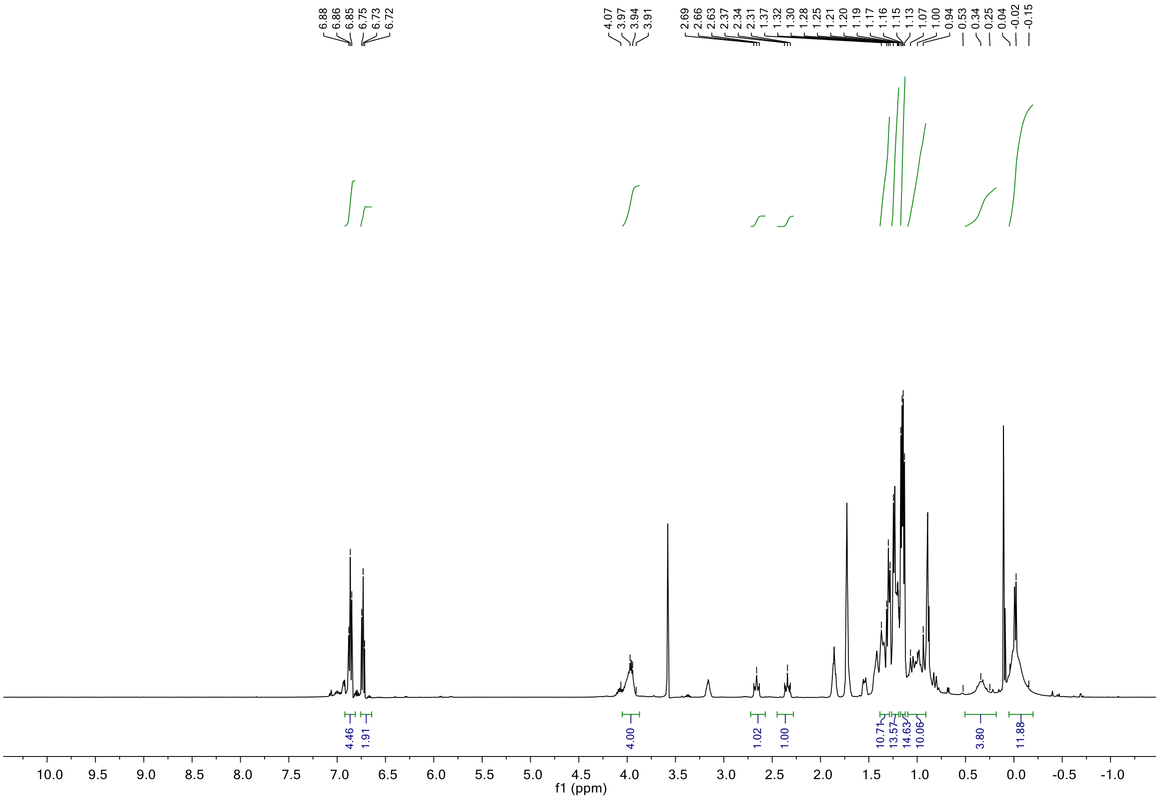

Figure S26. $^{13}C\{^{1}H\}$ NMR spectrum (126 MHz, 298K, d_8-THF) of $[\{SiN^{Dipp}\}AlN^{Cy}C\text{-}N^{Cy}]K$ (**19**).

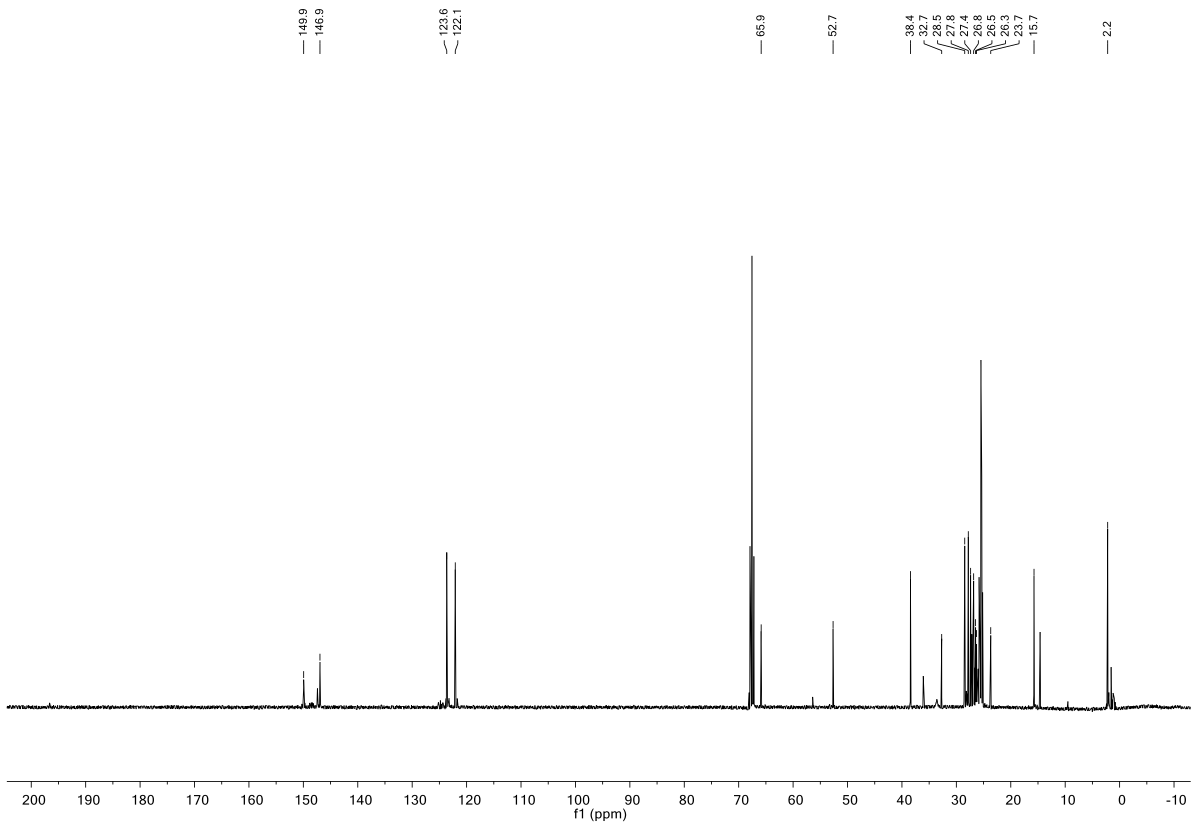

Synthesis of $\{SiN^{Dipp}\}$Al-K(18-cr-6) (**20**)

A solution of 18-crown-6 (18-cr-6, 0.089 g, 0.335 mmol) in toluene (10 mL) was added to a solution of compound **3** (0.180 g, 0.168 mmol) in toluene (5 mL) resulting in the formation of an orange solution. Removal of the volatile components gave an orange oil which was extracted into methylcyclohexane (5 mL). Orange crystals of **20** formed after 30 minutes at room temperature. No meaningful result could be obtained for elemental analysis after multiple attempts. Yield 0.198 g, 74%. ^{1}H NMR (500 MHz, C_6D_6): δ 7.15 (d, J = 7.2 Hz, 4H, *m*-C_6H_3), 6.92 (t, J = 7.2 Hz, 2H, *p*-C_6H_3), 4.43 (sept, J = 7.5 Hz, 4H, C*H*Me_2), 3.00 (s, 24H, 18c6-CH_2), 1.55, 1.47 (d, J = 7.5 Hz, 12H, CH*Me*$_2$), 1.37 (s, 4H, SiCH_2), 0.49 (s, 12H, Si*Me*$_2$). $^{13}C\{^{1}H\}$ NMR (125 MHz, C_6D_6): δ 151.7, 147.8, 122.5, 120.6 (*C*$_6H_3$), 70.3 (18c6-*C*H_2), 28.3, 25.8, 24.9 (*C*HMe_2 and CH*Me*$_2$), 15.7 (Si*C*H_2), 2.6 (Si*Me*$_2$).

Figure S27. ^{1}H NMR (500 MHz, 298K,C_6D_6). spectrum of **20**.

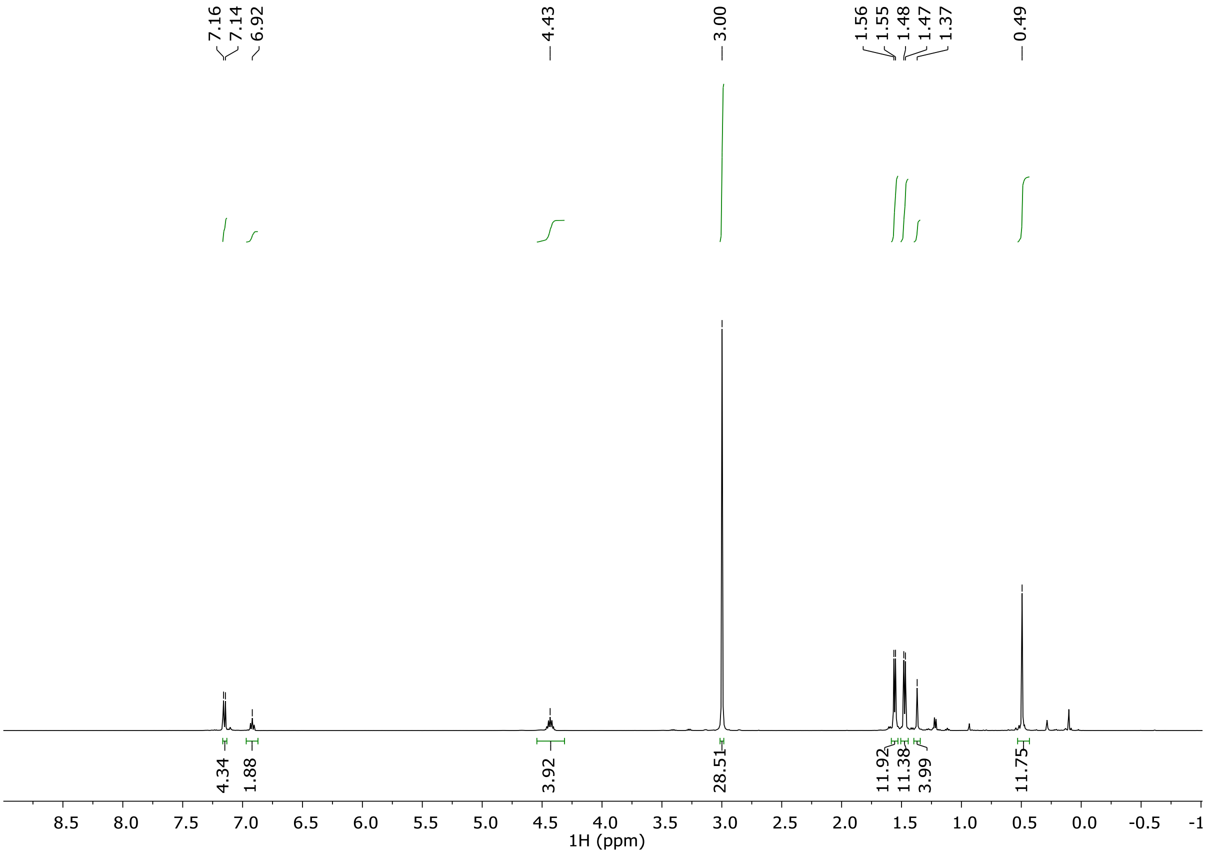

Figure S28. ^{13}C{^{1}H} NMR spectrum (126 MHz, 298K, C_6D_6) of **20**.

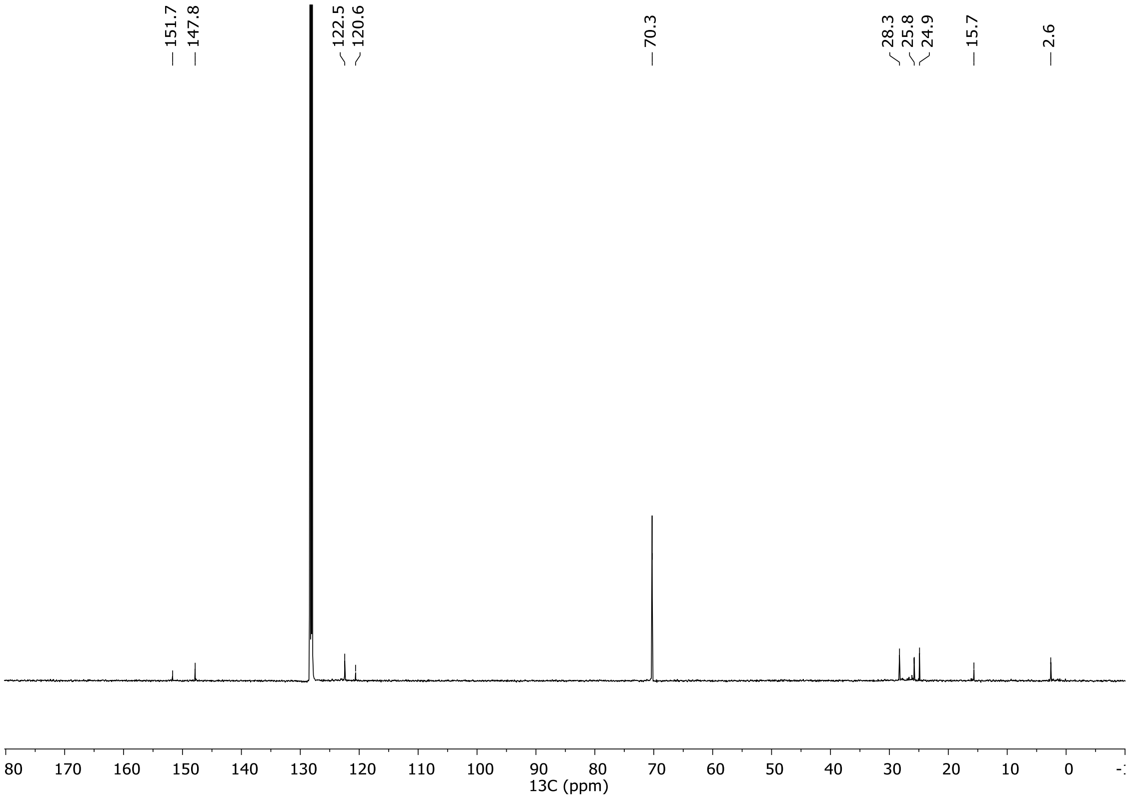

Synthesis of $\{SiN^{Dipp}\}Al-C\{NCy_2\}K(18-cr-6)$ (**21**).

18-crown-6 (18-cr-6, 25 mg, 0.095 mmol) was added to a J. Youngs NMR tube charged with a solution of **3** (53 mg, 0.048 mmol) in C_6D_6 (0.5 mL), resulting in the immediate formation of an orange solution. Dicyclohexylcarbodiimide (20 mg) was then added to the solution. Colorless crystals suitable for X-ray diffraction analysis were observed to crystallize from the reaction mixture. The crystals were then collected and washed with hexane before removal of all volatiles to give compound **21** as an off-white powder. Yield 56 mg, 57%. No meaningful result was obtained for elemental analysis after several attempts. ^{1}H NMR (500 MHz, d_8-THF): δ 6.86-6.81 (m, 4H, *m*-C_6H_3), 6.72-6.68 (m, 2H, *p*-C_6H_3), 4.08 – 3.86 (m, 4H, C*H*Me$_2$), 3.62 (s, 24H, OC_2H_4O), 2.92 (m, 1H, N-C*H* of cyclohexyl), 2.28 (m, 1H, N-C*H* of cyclohexyl), 1.48-1.27 (m, 10H, *H*-cyclohexyl), 1.24-1.23 (m, 6H, CH*Me*$_2$), 1.21-1.20 (m, 6H, CH*Me*$_2$), 1.16-1.14 (m, 6H, CH*Me*$_2$), 1.13-1.11 (m, 6H, CH*Me*$_2$), 1.06-0.87 (m, 10H, *H*-cyclohexyl), 0.87 – 0.70 (br, 2H, SiC*H*$_2$), 0.50-0.32 (br, 2H, SiC*H*$_2$), 0.15 - –0.23 (br, 12H, Si*Me*$_2$). ^{13}C{^{1}H} NMR (125 MHz, d_8-THF): δ 150.1 (*i*-C_6H_3), 147.0 (*o*-C_6H_3), 123.5 (*m*-C_6H_3), 121.8 (*p*-C_6H_3), 71.3 (OC_2H_4O), 51.9 ($NC_{cyclohexyl}$), 38.3 ($NC_{cyclohexyl}$), 28.4 (*C*HMe$_2$), 28.1($C_{cyclohexyl}$), 27.8 ($C_{cyclohexyl}$), 27.6($C_{cyclohexyl}$), 27.4 ($C_{cyclohexyl}$), 27.2 ($C_{cyclohexyl}$), 26.8 ($C_{cyclohexyl}$), 26.6 (CH*Me*$_2$), 26.3 (CH*Me*$_2$), 15.8 (Si*C*H$_2$), 2.3 (Si*Me*$_2$). Al*C*N_2 not observed.

Figure S29. ^{1}H NMR spectrum (500 MHz, 298K, d_8-THF) of (**21**).

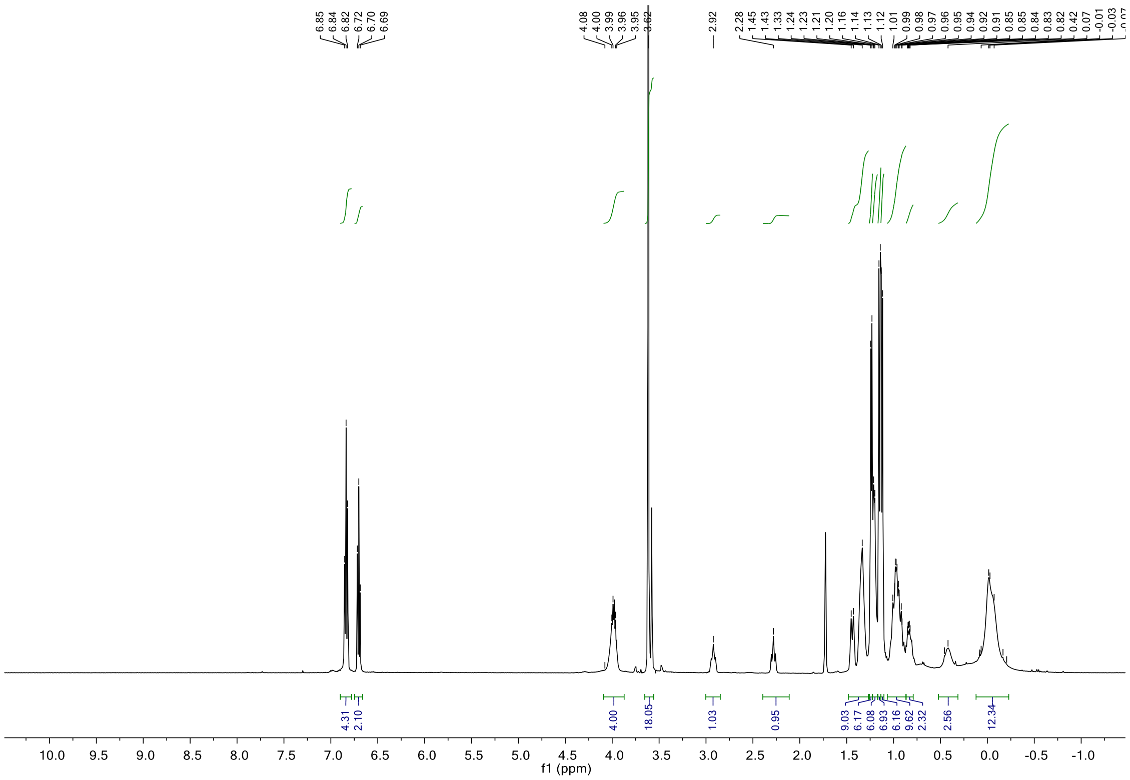

Figure S30. $^{13}C\{^1H\}$ NMR spectrum (126 MHz, 298K, d_8-THF) of $\{SiN^{Dipp}\}$Al-C$\{NCy_2\}$K(18-cr-6) (**21**).

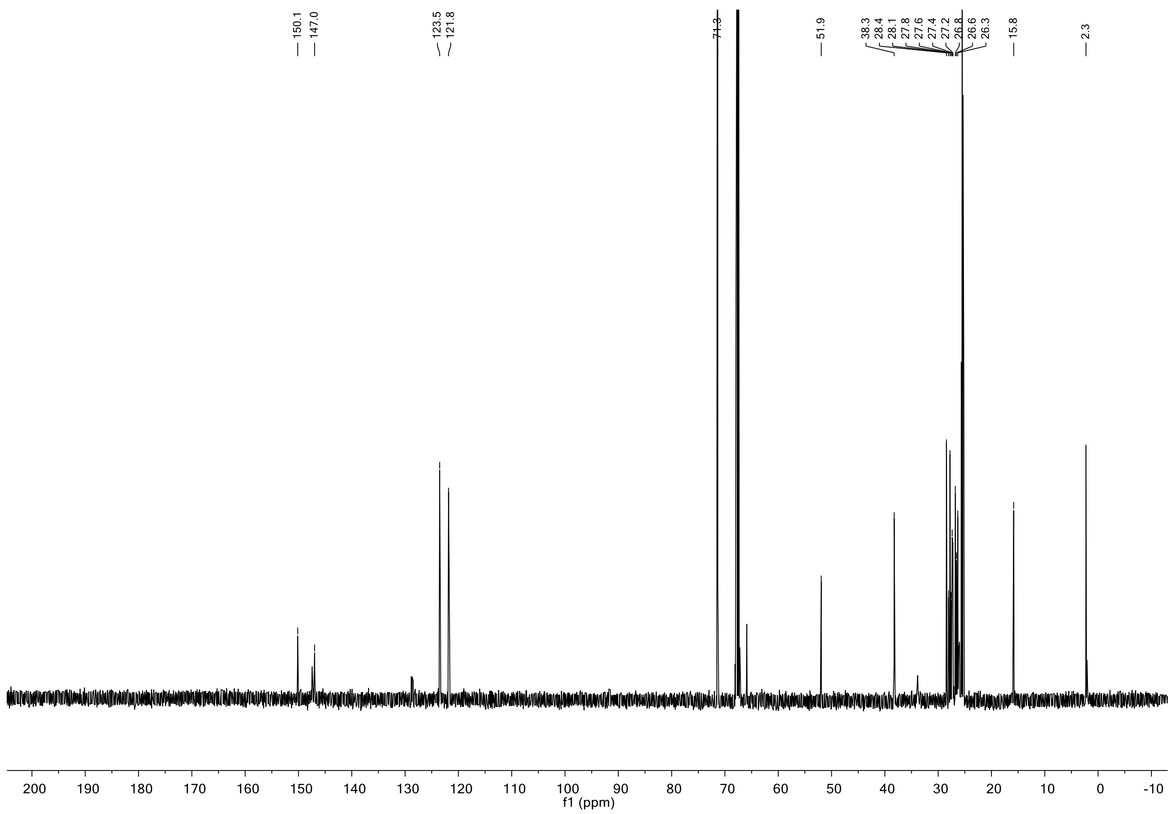

Synthesis of [$\{SiN^{Dipp}\}$Al][K(18-cr-6)$_{1.5}$] (**22**)

18-crown-6 (18-cr-6, 0.027 g, 0.101 mmol) was added to a J. Youngs NMR tube charged with a solution of **3** (0.036 g, 0.033 mmol) in C_6D_6 (0.5 mL) resulting in the immediate formation of an orange solution. Storage of the solution at room temperature resulted in the gradual formation of orange crystals of **21**. No meaningful result could be obtained for elemental analysis after multiple attempts. Yield 0.045 g, 71 %. ^{1}H NMR (500 MHz, C_6D_6): δ 7.14 (d, *J* = 7.2Hz, 4H, *m*-C_6H_3), 6.91 (t, *J* = 7.2 Hz, 2H, *p*-C_6H_3), 4.43 (sept, *J* = 7.5 Hz, 4H, C*H*Me$_2$), 3.17 (s, 36H, 18-cr-6-OCH_2), 1.55, 1.47 (d, *J* = 7.5, 12H, CH*Me*$_2$), 1.37 (s, 4H, SiCH_2), 0.50 (s, 12H, Si*Me*$_2$). $^{13}C\{^1H\}$ NMR (125 MHz, C_6D_6): δ 151.6, 147.7, 122.3, 120.5 (C_6H$_3$), 70.2 (18c6-*C*H$_2$), 28.2, 25.7, 24.8 (*C*HMe$_2$ and CH*Me*$_2$), 15.5 (Si*C*H$_2$), 2.5 (Si*Me*$_2$).

Figure S31. ^{1}H NMR spectrum (500 MHz, 298K, C_6D_6) of (**22**, * = hexane).

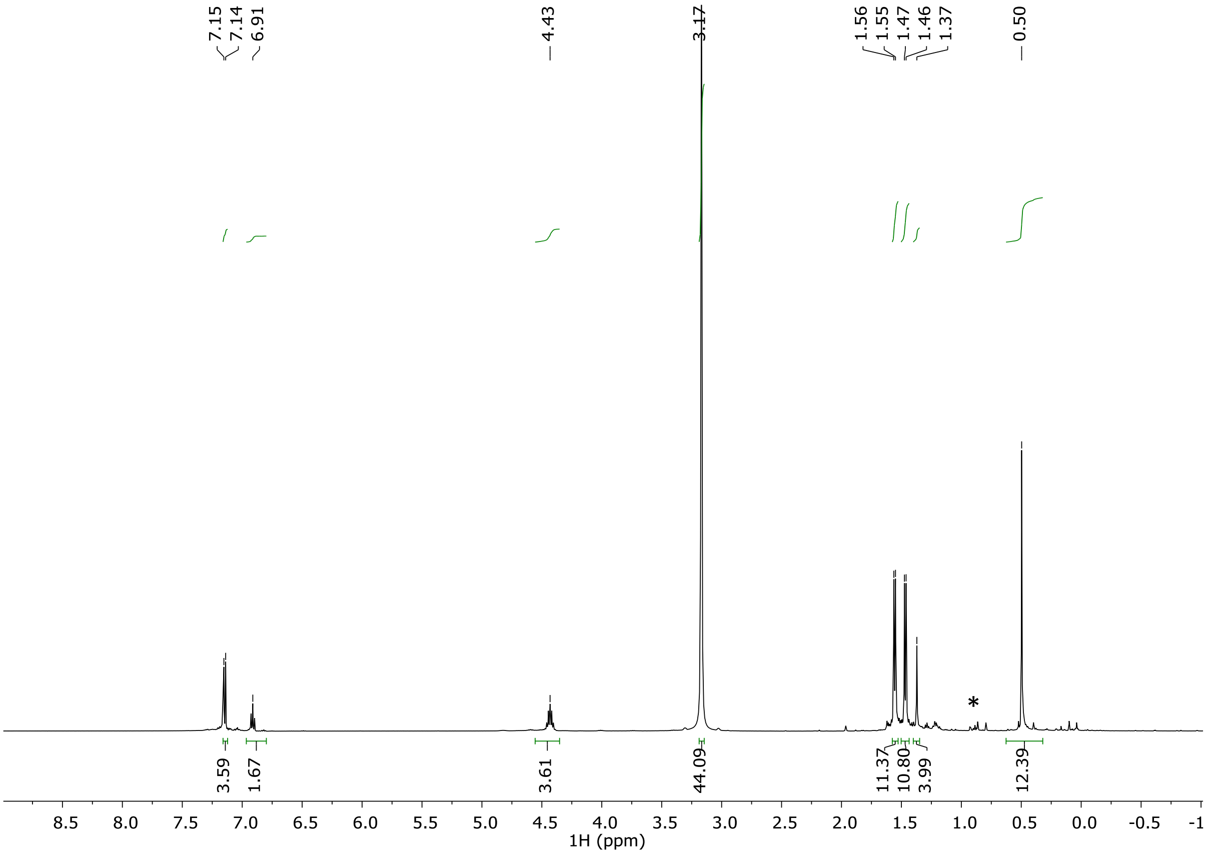

Figure S32. $^{13}C\{^{1}H\}$ NMR spectrum (126 MHz, 298K, C_6D_6) of (**22**).

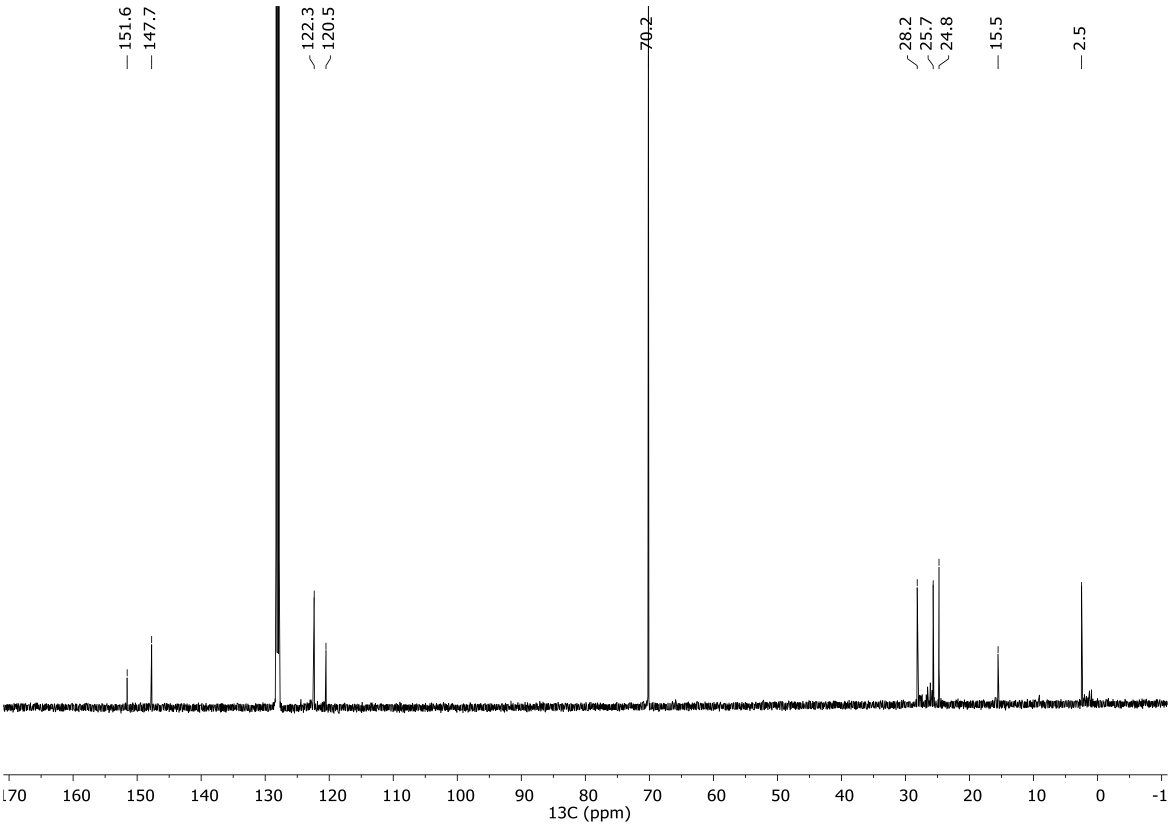

Synthesis of [{SiN^{Dipp}}Al][K(2,2,2-crypt)] (**23**)

2,2,2-cryptand (0.020 g, 0.054 mmol) was added to a J. Youngs NMR tube charged with a solution of **3** (0.030 g, 0.027 mmol) in C_6D_6 (0.5 mL) resulting in the immediate formation of two immiscible layers, a colorless top layer and an orange bottom layer. Removal of the volatiles *in vacuo* gave a viscous orange oil which was extracted into 2,2,5,5-tetramethyltetrahydrofuran (TMTHF). Storage of the orange solution at room temperature resulted in the gradual formation of orange crystals of (**23**). No meaningful result could be obtained for elemental analysis after multiple attempts. Yield 0.043 g, 86 %. ^{1}H NMR (500 MHz, C_6D_6): δ 6.84 (d, *J* = 6.8 Hz, 4H, *m*-C_6H_3), 6.63 (t, *J* = 6.8 Hz, 2H, *p*-C_6H_3), 4.15 (sept, *J* = 7.2 Hz, 4H, C*H*Me$_2$), 3.51 – 3.45 (m, 24H, crypt-OCH_2), 2.49 (m, 12H, crypt-NCH_2), 1.17 (m, 24H, CHMe_2), 0.86 (s, 4H, SiCH_2), –0.11 (s, 12H, SiMe_2). ^{13}C{^{1}H} NMR (125 MHz, C_6D_6): δ 152.1, 148.1, 122.5, 120.5 (C_6H$_3$), 68.6, 54.9, 39.7 (crypt-O*C*H$_2$ and crypt-N*C*H$_2$), 30.3, 28.4, 26.7 (*C*HMe$_2$ and CH*Me$_2$*), 14.3 (Si*C*H$_2$), 2.5 (Si*Me$_2$*).

Figure S33. ^{1}H NMR spectrum (500 MHz, 298K, d_8-THF) of (**23**) .*TMTHF.

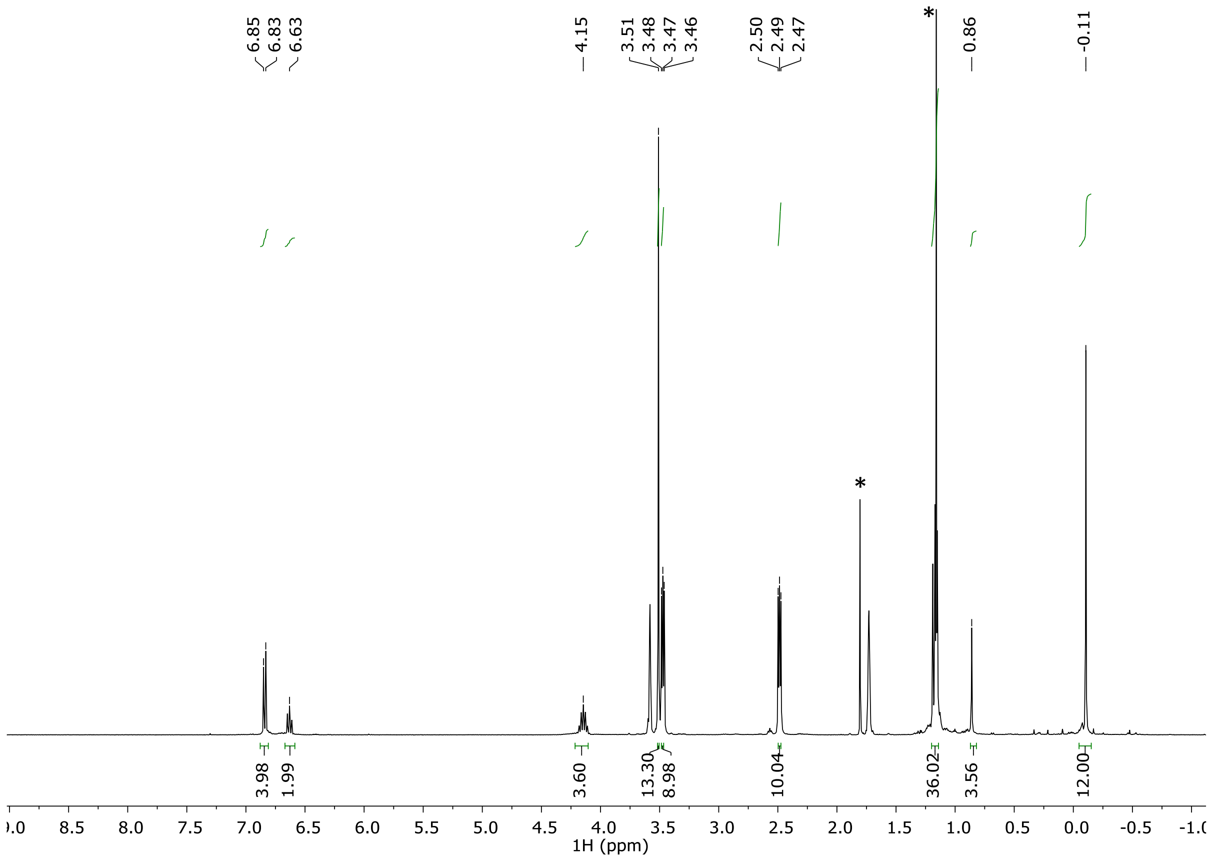

Figure S34. $^{13}C\{^{1}H\}$ NMR spectrum (126 MHz, 298K, d_8-THF) of (**23**).

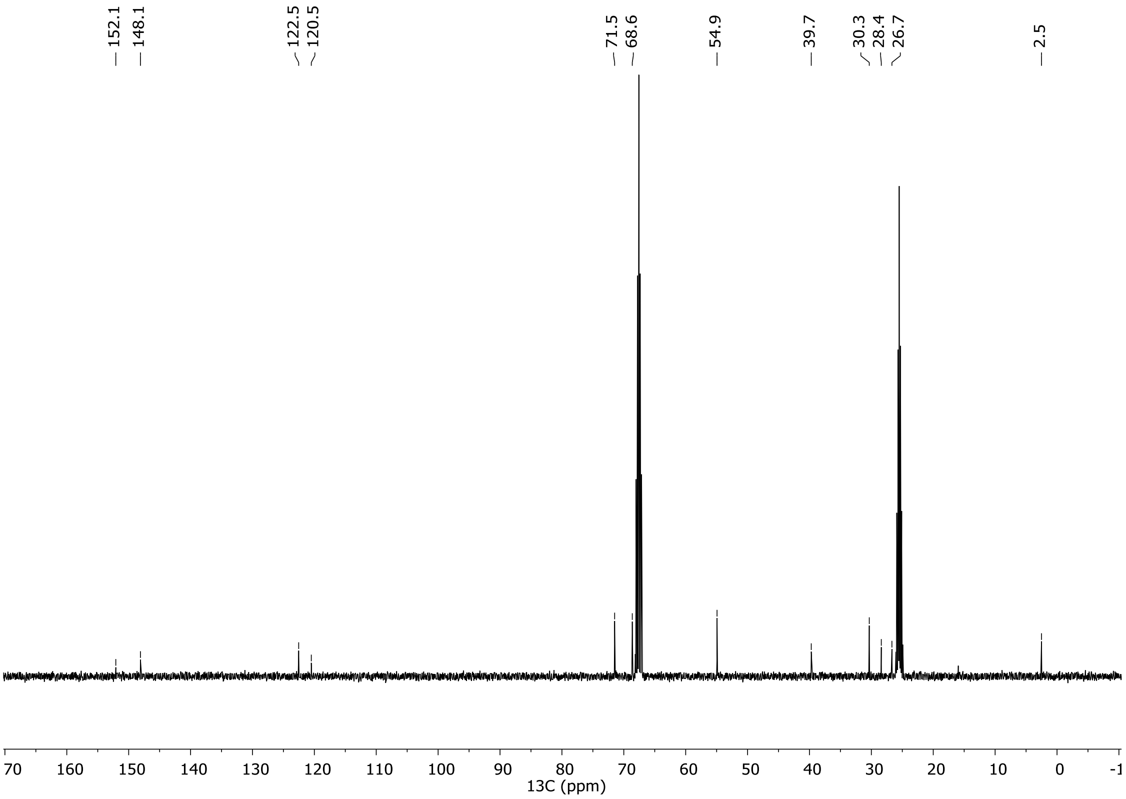

X-ray Crystallography

Single Crystal X–ray diffraction data for compounds **10** – **13**, **17** – **23** were collected on a SuperNova, EosS2 diffractometer using CuKα (λ = 1.54184 Å) radiation throughout. Data for compound **14** were collected on a New Xcalibur, EosS2 diffractometer using MoKα (λ = 0.71073 Å) radiation. The crystals were maintained at 150 K during data collection. Using Olex2,[2] the structures were solved with the olex2.solve[3] structure solution program or ShelXT and refined with the ShelXL[4] refinement package using Least Squares minimization.

The asymmetric unit in **12** contains half of a molecule plus a region of solvent. The aluminium centres are both coincident with a two-fold rotation axis implicit in the space group symmetry. The potassium cation was modelled over two positions in a 55:45 ratio, while C23, C24, and C25 were each modelled to take account of 80:20 disorder. Distance and ADP restraints were used in disordered regions to assist convergence. The solvent was heavily disordered, which is unsurprising, as it located in structural channels which are parallel to the *c*-axis. Ultimately, this was treated using the solvent mask algorithm available in Olex-2 and an allowance of 12 molecules of hexane per unit cell has been made in the formula as presented.

The asymmetric unit in structure of **13** comprises half of a dimer molecule plus one benzene molecule. The remainder of the dimer can be generated by virtue of a twofold rotation axis implicit in the space group symmetry.

The motif in **15** contains one aluminium centre and represents part of a polymeric structure. The hydrogens attached to C27 were located and refined at a fixed distance of 0.96Å from the parent atom.

The hydrogen atoms attached to C31, C32 and C36 in **17** were located and refined without restraints.

The asymmetric unit in **18** comprises half of a dimer molecule and 2 guest benzenes. The hydrogens attached to C32, C33 and C36 were readily located and each refined at a distance of 0.98Å from the relevant carbon atom.

C29 and C30 were treated for 50:50 disorder in the structure of **19**, where the asymmetric unit comprises just half of a molecule. Distance and ADP restraints were used in the disordered region, to assist convergence.

The largest residual electron density peak (close to the order of one electron per $Å^{-3}$) in the structure of **20** lies 1.56 Å from the aluminium centre, and 2.68 Å from K1. In the absence of any spectroscopic or chemical evidence for the presence of a minor product, this was treated as a spurious artefact.

In the structure of **21**, all atoms in the cyclohexyl moiety based on C38 (with the exception of C38 itself) were modelled to take 55:45 disorder into account. Distance restraints were employed in the disordered components to assist convergence. Two molecules of benzene were also found to be present in the asymmetric unit.

The asymmetric unit in **22** contains one aluminium based anion and half of a dication. The latter contains one potassium centre and 1.5 crown-ethers. A crystallographic inversion centre proximate to the half-crown moiety serves to generate the remainder of the cation by bridging between the potassium centres. The full crown present in the asymmetric unit was disordered but, with the inclusion of distance and ADP restraints pertaining to fractional occupancy atoms, this resolved into 2 components with an occupancy ratio of 63:37. The residual maximum in the difference-Fourier, electron-density map, at 2.56Å from C32A, appears to be spurious and is most likely an artefact.

The asymmetric unit in **23** contains one anion, one cryptand-based cation and one molecule of tetramethyl-THF. Four carbons in the solvent were treated for 50:50 disorder and, with the inclusion of some distance and ADP restraints, the model converged sensibly in this region. The highest residual electron density peak lies approximately 1.38 Å from the aluminium centre. In the absence of any chemical evidence for a minor second species being present, this appears to be an artefact. When the gross structure is viewed along the *c*-axis, there are small channel-type pathways evident, which coincide with this residual electron density maximum.

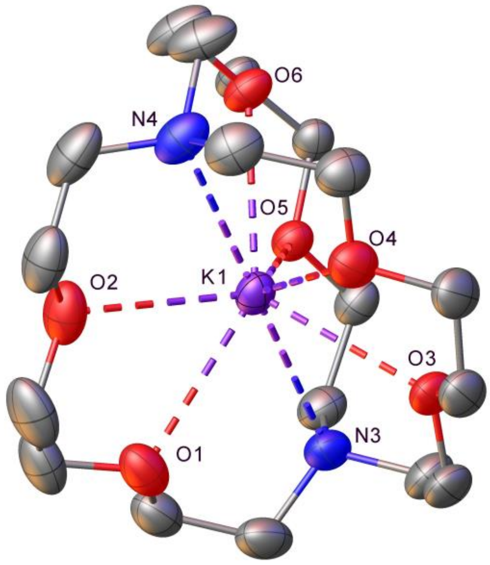

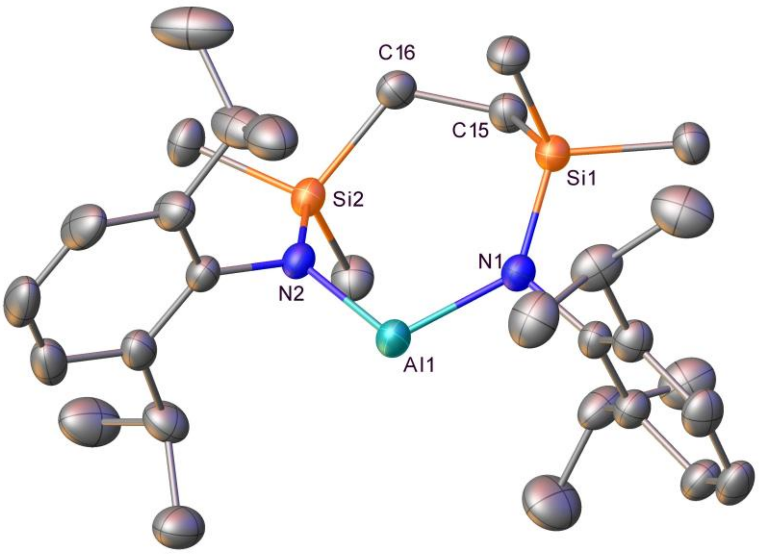

Figure S35. Displacement ellipsoid plot (50% probability) of compound **23**. Hydrogen atoms and an occluded molecule of tetramethyl-THF are omitted for clarity.

Table S1: Crystal data and structure refinement for compounds **10 - 13**.

Compound	**10**	**11**	**12**	**13**
Empirical formula	$C_{24}H_{38}AlIN_2Si_2$	$C_{48}H_{76}Al_2N_4Si_4$	$C_{52}H_{85.33}Al_2K_2N_4Si_4$	$C_{66}H_{106}Al_2N_4Si_4$
Formula weight	564.62	875.44	1011.09	1121.86
Crystal system	triclinic	triclinic	trigonal	monoclinic
Space group	*P*-1	*P*-1	*R*-3*c*	*I*2/*a*
a/Å	8.3928(2)	11.6498(2)	22.74280(10)	23.6172(2)
b/Å	8.4372(3)	12.1660(3)	22.74280(10)	13.6850(1)
c/Å	22.3918(6)	18.5161(4)	62.4273(4)	22.0608(2)
α/°	90.019(2)	87.461(2)	90	90
β/°	97.901(2)	85.591(1)	90	113.771(1)
γ/°	117.431(3)	76.997(2)	120	90
Volume/Å^3	1390.31(8)	2548.47(10)	27963.6(3)	6525.19(10)
Z	2	2	18	4
ρ_{calc} g/cm^3	1.349	1.141	1.081	1.142
μ/mm^{-1}	10.269	1.678	2.610	1.411
F(000)	580.0	948.0	9816.0	2448.0
Crystal size/mm^3	0.283 × 0.173 × 0.14	0.155 × 0.135 × 0.088	0.463 × 0.247 × 0.222	0.287 × 0.179 × 0.123
2θ range /°	7.994 to 146.104	7.46 to 146.314	5.306 to 146.164	7.646 to 146.282
Index ranges	$-10 \leq h \leq 10$, $-10 \leq k \leq 10$, $-27 \leq l \leq 25$	$-10 \leq h \leq 14$, $-15 \leq k \leq 15$, $-22 \leq l \leq 22$	$-27 \leq h \leq 27$, $-28 \leq k \leq 28$, $-77 \leq l \leq 49$	$-28 \leq h \leq 29$, $-16 \leq k \leq 16$, $-26 \leq l \leq 27$
Reflections collected	14599	31567	85335	32466
Independent reflections	5540 [R_{int} = 0.0411, R_{sigma} = 0.0443]	10147 [R_{int} = 0.0365, R_{sigma} = 0.0395]	6240 [R_{int} = 0.0337, R_{sigma} = 0.0117]	6472 [R_{int} = 0.0315, R_{sigma} = 0.0226]
Data/restraints/parameters	5540/0/281	10147/0/543	6240/27/320	6472/0/355
Goodness-of-fit on F^2	1.135	1.043	1.029	1.044
Final R indexes [I>=2σ (I)]	R_1 = 0.0525, wR_2 = 0.1368	R_1 = 0.0356, wR_2 = 0.0914	R_1 = 0.0503, wR_2 = 0.1525	R_1 = 0.0348, wR_2 = 0.0948
Final R indexes [all data]	R_1 = 0.0544, wR_2 = 0.1379	R_1 = 0.0415, wR_2 = 0.0954	R_1 = 0.0526, wR_2 = 0.1554	R_1 = 0.0364, wR_2 = 0.0963
Largest diff. peak/hole / e Å^{-3}	1.20/-0.94	0.38/-0.23	0.41/-0.27	0.30/-0.37

Table S2: Crystal data and structure refinement for compounds **14**, **15**, **17** and **18**.

Compound	**14**	**15**	**17**	**18**
Empirical formula	$C_{66}H_{106}Al_2N_4Si_4$	$C_{48}H_{65}AlBKN_2Si_2$	$C_{49}H_{65}AlN_2Si_2$	$C_{49}H_{76}AlKN_4Si_2$
Formula weight	1121.86	803.09	765.19	843.39
Crystal system	monoclinic	monoclinic	monoclinic	triclinic
Space group	*I*2/*a*	$P2_1/n$	$P2_1/c$	*P*-1
a/Å	23.6172(2)	14.7608(4)	10.3005(1)	9.81740(10)
b/Å	13.6850(1)	16.1650(5)	34.6564(3)	16.2583(3)
c/Å	22.0608(2)	19.7217(5)	12.8827(1)	16.4160(3)
α/°	90	90	90	97.097(2)
β/°	113.771(1)	100.406(3)	98.702(1)	103.6310(10)
γ/°	90	90	90	101.7010(10)
Volume/$Å^3$	6525.19(10)	4628.4(2)	4545.90(7)	2452.76(7)
Z	4	4	4	2
ρ_{calc} g/cm^3	1.142	1.153	1.118	1.142
μ/mm^{-1}	1.411	1.928	1.140	1.852
F(000)	2448.0	1728.0	1656.0	916.0
Crystal size/mm^3	0.287 × 0.179 × 0.123	0.089 × 0.048 × 0.013	0.402 × 0.315 × 0.205	0.153 × 0.126 × 0.095
2θ range /°	7.646 to 146.282	6.914 to 136.486	5.1 to 146.2	5.632 to 146.18
Index ranges	-28 ≤ h ≤ 29, -16 ≤ k ≤ 16, -26 ≤ l ≤ 27	-17 ≤ h ≤ 17, -19 ≤ k ≤ 16, -23 ≤ l ≤ 22	-11 ≤ h ≤ 12, -36 ≤ k ≤ 43, -15 ≤ l ≤ 14	-12 ≤ h ≤ 9, -20 ≤ k ≤ 20, -20 ≤ l ≤ 20
Reflections collected	32466	33245	61867	28317
Independent reflections	6472 [R_{int} = 0.0315, R_{sigma} = 0.0226]	8370 [R_{int} = 0.0686, R_{sigma} = 0.0635]	9058 [R_{int} = 0.0492, R_{sigma} = 0.0258]	9699 [R_{int} = 0.0310, R_{sigma} = 0.0353]
Data/restraints/parameters	6472/0/355	8370/3/519	9058/0/520	9699/7/556
Goodness-of-fit on F^2	1.044	1.017	1.022	1.019
Final *R* indexes [*I*>=2σ (I)]	R_1 = 0.0348, wR_2 = 0.0948	R_1 = 0.0520, wR_2 = 0.1246	R_1 = 0.0441, wR_2 = 0.1190	R_1 = 0.0338, wR_2 = 0.0836
Final *R* indexes [all data]	R_1 = 0.0364, wR_2 = 0.0963	R_1 = 0.0804, wR_2 = 0.1398	R_1 = 0.0480, wR_2 = 0.1232	R_1 = 0.0395, wR_2 = 0.0872
Largest diff. peak/hole / e $Å^{-3}$	0.30/-0.37	0.37/-0.26	0.38/-0.33	0.36/-0.22

Table S3: Crystal data and structure refinement for compounds **19 - 22**.

Compound	**19**	**20**	**21**	**22**
Empirical formula	$C_{43}H_{72}AlKN_4Si_2$	$C_{42}H_{74}AlKN_2O_6Si_2$	$C_{67}H_{108}AlKN_4O_6Si_2$	$C_{48}H_{86}AlKN_2O_9Si_2$
Formula weight	767.30	825.29	1187.83	957.44
Crystal system	monoclinic	monoclinic	monoclinic	monoclinic
Space group	$P2_1/n$	$P2_1/n$	$P2_1/c$	$P2_1/n$
a/Å	15.5250(2)	12.0795(1)	19.8752(2)	10.5444(1)
b/Å	14.1393(1)	16.8143(2)	15.16620(10)	22.3042(2)
c/Å	20.1563(2)	23.9867(2)	25.2290(2)	23.7719(2)
α/°	90	90	90	90
β/°	93.8290(10)	98.495(1)	113.2150(10)	92.063(1)
γ/°	90	90	90	90
Volume/Å^3	4414.69(8)	4818.45(8)	6989.06(11)	5587.16(9)
Z	4	4	4	4
ρ_{calc} g/cm^3	1.154	1.138	1.129	1.138
μ/mm^{-1}	2.008	1.955	1.498	1.791
F(000)	1672.0	1792.0	2584.0	2080.0
Crystal size/mm^3	0.141 × 0.117 × 0.067	0.194 × 0.149 × 0.128	0.367 × 0.243 × 0.215	0.337 × 0.256 × 0.105
2θ range /°	6.966 to 146.296	6.444 to 146.064	6.964 to 146.874	5.434 to 146.234
Index ranges	-19 ≤ h ≤ 18, -17 ≤ k ≤ 17, -24 ≤ l ≤ 23	-14 ≤ h ≤ 14, -17 ≤ k ≤ 20, -27 ≤ l ≤ 29	-18 ≤ h ≤ 24, -18 ≤ k ≤ 18, -31 ≤ l ≤ 31	-13 ≤ h ≤ 12, -27 ≤ k ≤ 27, -29 ≤ l ≤ 29
Reflections collected	51438	35422	75580	39541
Independent reflections	8778 [R_{int} = 0.0420, R_{sigma} = 0.0277]	9564 [R_{int} = 0.0353, R_{sigma} = 0.0352]	13914 [R_{int} = 0.0277, R_{sigma} = 0.0204]	11061 [R_{int} = 0.0418, R_{sigma} = 0.0391]
Data/restraints/parameters	8778/14/508	9564/0/499	13914/11/787	11061/54/742
Goodness-of-fit on F^2	1.033	1.041	1.033	1.019
Final R indexes [I>=2σ (I)]	R_1 = 0.0412, wR_2 = 0.1094	R_1 = 0.0420, wR_2 = 0.1067	R_1 = 0.0324, wR_2 = 0.0842	R_1 = 0.0442, wR_2 = 0.1195
Final R indexes [all data]	R_1 = 0.0474, wR_2 = 0.1145	R_1 = 0.0489, wR_2 = 0.1120	R_1 = 0.0346, wR_2 = 0.0862	R_1 = 0.0493, wR_2 = 0.1249
Largest diff. peak/hole / e Å^{-3}	0.39/-0.27	0.65/-0.31	0.28/-0.23	1.42/-0.23

Table S4: Crystal data and structure refinement for compound **23**.

Compound	**23**
Empirical formula	$C_{56}H_{102}AlKN_4O_7Si_2$
Formula weight	1065.67
Crystal system	monoclinic
Space group	$P2_1/c$
a/Å	18.0696(4)
b/Å	16.3737(3)
c/Å	23.0706(6)
α/°	90
β/°	108.756(3)
γ/°	90
Volume/Å^3	6463.3(3)
Z	4
ρ_{calc} g/cm^3	1.095
μ/mm^{-1}	1.577
F(000)	2328.0
Crystal size/mm^3	0.261 × 0.127 × 0.088
2θ range /°	7.474 to 146.126
Index ranges	$-17 \leq h \leq 22$, $-20 \leq k \leq 19$, $-28 \leq l \leq 28$
Reflections collected	46280
Independent reflections	12820 [R_{int} = 0.0447, R_{sigma} = 0.0381]
Data/restraints/parameters	12820/19/694
Goodness-of-fit on F^2	1.053
Final R indexes [I>=2σ (I)]	R_1 = 0.0698, wR_2 = 0.1930
Final R indexes [all data]	R_1 = 0.0882, wR_2 = 0.2118
Largest diff. peak/hole / e Å^{-3}	1.09/-0.22

Computational Details / Methodology

DFT calculations were run with Gaussian 09 (Revision D.01).[5] The K, Al, Si, S and I centres were described with the Stuttgart RECPs and associated basis sets,[6] and 6-31G** basis sets were used for all other atoms (BS1).[7] A polarization function was also added to Al (ζ_d = 0.190), Si (ζ_d = 0.284), S (ζ_d = 0.503) and l (ζ_d = 0.289). All energies were recomputed with cc-pVTZ-PP for I and 6-311++G** basis sets for other atoms (BS2). Initial BP86[8] optimizations were performed using the 'grid = ultrafine' option, with all stationary points being fully characterized via analytical frequency calculations as minima (all positive eigenvalues). Kohn-Sham frontier orbital analysis was performed on the BP86-optimized geometries of the Al species.

Table S5. HOMO, LUMO and LUMO+4 energies (in eV) of the BP86-optimised alumanyl anion form compounds **22** and **23**. The calculated ∠N–Al–N bite angles are given in degrees.

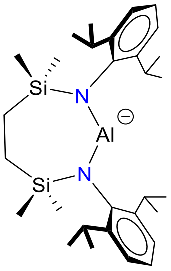

	**[22]$^-$ / [23]$^-$**
HOMO	-0.13
LUMO	+2.05
LUMO+4	+2.55
∠N–Al–N [°]	107.6

Table S6. DFT calculated frontier orbitals of the BP86-optimized Al anion species in compounds **22** and **23**. LUMO+4 is shown to depict the Al empty p_z orbital.

**Species**	**HOMO**	**LUMO**	**LUMO+4**
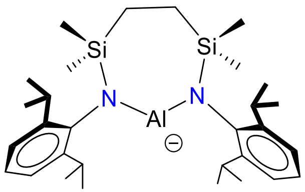	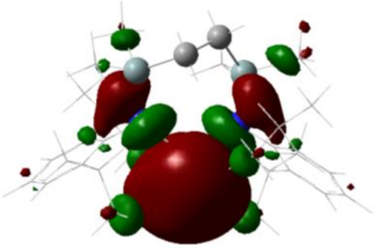	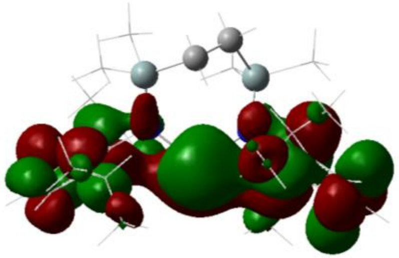	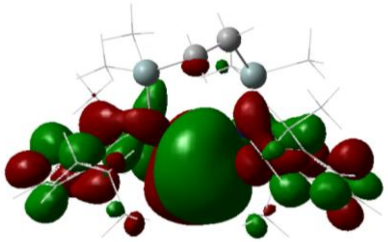

Cartesian Coordinates and Computed Energies (in Hartrees)

[{SiN^{Dipp}}Al]$^{-}$

Anion of **22** or **23**

```
SCF (BP86) Energy = -1292.82960022
Enthalpy 0K = -1292.117654
Enthalpy 298K = -1292.071277
Free Energy 298K = -1292.194808
Lowest Frequency = 20.8367 cm-1
Second Frequency = 23.7373 cm-1

Si -1.74095  0.87457  1.81623
Si  1.74100 -0.87370  1.81642
Al -0.00001  0.00075 -1.02394
N  -1.59061  0.16360  0.19928
N   1.59053 -0.16322  0.19926
C  -2.81330 -0.11368 -0.48996
C  -3.46031  0.87106 -1.31140
C  -4.65303  0.54528 -1.98721
H  -5.13476  1.31102 -2.60916
C  -5.23146 -0.72503 -1.89388
H  -6.15945 -0.95730 -2.42975
C  -4.59467 -1.69866 -1.11429
H  -5.02973 -2.70395 -1.04708
C  -3.40539 -1.41978 -0.41640
C  -2.88932  2.27871 -1.49071
H  -1.96362  2.32264 -0.89122
C  -2.49479  2.55011 -2.95908
H  -1.74151  1.81526 -3.29066
H  -2.06234  3.56256 -3.06562
H  -3.36867  2.48422 -3.63385
C  -3.85443  3.36805 -0.97091
H  -4.80230  3.37477 -1.54052
H  -3.40129  4.37186 -1.07004
H  -4.10853  3.21205  0.09190
C  -2.72936 -2.53663  0.37699
H  -1.96708 -2.04476  1.00650
C  -1.98798 -3.50147 -0.57575
H  -2.69456 -3.98681 -1.27418
H  -1.46173 -4.29492 -0.01267
H  -1.24393 -2.95087 -1.17993
C  -3.69443 -3.30587  1.30241
H  -4.22873 -2.62362  1.98637
H  -3.13902 -4.03920  1.91521
H  -4.45616 -3.86898  0.73205
C  -3.56596  0.86772  2.40386
H  -4.23130  1.35333  1.67097
H  -3.66183  1.40343  3.36582
H  -3.93660 -0.16165  2.54861
C  -1.13896  2.69368  1.88015
H  -0.13129  2.79047  1.44318
H  -1.09597  3.06290  2.92149
H  -1.81349  3.35675  1.31235
C  -0.76969 -0.06291  3.18050
H  -1.18131  0.32875  4.13588
H  -1.07013 -1.12826  3.14985
C   0.76943  0.06371  3.18053
H   1.18101 -0.32810  4.13588
H   1.06985  1.12907  3.15007
C   1.13977 -2.69306  1.88119
H   0.13229 -2.79059  1.44395
H   1.09656 -3.06152  2.92280
H   1.81478 -3.35627  1.31414
C   3.56600 -0.86604  2.40411
H   4.23144 -1.35209  1.67159
H   3.66196 -1.40103  3.36646
H   3.93646  0.16350  2.54809
C   2.81325  0.11345 -0.49022
C   3.45977 -0.87174 -1.31149
C   4.65243 -0.54654 -1.98768
H   5.13377 -1.31261 -2.60953
C   5.23127  0.72362 -1.89486
H   6.15919  0.95545 -2.43104
C   4.59500  1.69766 -1.11537
H   5.03042  2.70282 -1.04852
C   3.40581  1.41936 -0.41710
C   2.88826 -2.27925 -1.49021
H   1.96275 -2.32269 -0.89039
C   2.49315 -2.55092 -2.95837
H   1.74001 -1.81590 -3.28992
H   2.06034 -3.56326 -3.06452
H   3.36683 -2.48547 -3.63345
C   3.85319 -3.36873 -0.97039
H   4.80084 -3.37599 -1.54037
H   3.39967 -4.37243 -1.06897
H   4.10775 -3.21242  0.09226
C   2.73042  2.53663  0.37627
H   1.96823  2.04515  1.00620
C   1.98901  3.50151 -0.57637
H   2.69549  3.98648 -1.27516
H   1.46323  4.29524 -0.01326
H   1.24456  2.95103 -1.18018
C   3.69610  3.30576  1.30116
H   4.23046  2.62349  1.98506
H   3.14115  4.03939  1.91402
H   4.45779  3.86850  0.73038
```

References

[1] R. J. Schwamm, M. P. Coles, M. S. Hill, M. F. Mahon, C. L. McMullin, N. A. Rajabi, A. S. S. Wilson, *Angew. Chem. Int. Ed.* **2020**, *59*, 3928-3932.

[2] O. V. Dolomanov, L. J. Bourhis, R. J. Gildea, J. A. K. Howard, H. Puschmann, *J. Appl. Cryst.* **2009**, *42*, 339-341.

[3] L. J. Bourhis, O. V. Dolomanov, R. J. Gildea, J. A. K. Howard, H. Puschmann, *Acta Cryst. a-Foundation and Advances*, **2015**, *71*, 59-75.

[4] G. M. Sheldrick, *Acta Cryst. Sect. C-Structural Chemistry*, **2015**, *71*, 3-8.

[5] Frisch, M. J.; Trucks, G. W.; Schlegel, H. B.; Scuseria, G. E.; Robb, M. A.; Cheeseman, J. R.; Scalmani, G.; Barone, V.; Mennucci, B.; Petersson, G. A.; Nakatsuji, H.; Caricato, M.; Li, X.; Hratchian, H. P.; Izmaylov, A. F.; Bloino, J.; Zheng, G.; Sonnenberg, J. L.; Hada, M.; Ehara, M.; Toyota, K.; Fukuda, R.; Hasegawa, J.; Ishida, M.; Nakajima, T.; Honda, Y.; Kitao, O.; Nakai, H.; Vreven, T.; Montgomery, J. A., Jr.; Peralta, J. E.; Ogliaro, F.; Bearpark, M.; Heyd, J. J.; Brothers, E.; Kudin, K. N.; Staroverov, V. N.; Kobayashi, R.; Normand, J.; Raghavachari, K.; Rendell, A.; Burant, J. C.; Iyengar, S. S.; Tomasi, J.; Cossi, M.; Rega, N.; Millam, J. M.; Klene, M.; Knox, J. E.; Cross, J. B.; Bakken, V.; Adamo, C.; Jaramillo, J.; Gomperts, R.; Stratmann, R. E.; Yazyev, O.; Austin, A. J.; Cammi, R.; Pomelli, C.; Ochterski, J. W.; Martin, R. L.; Morokuma, K.; Zakrzewski, V. G.; Voth, G. A.; Salvador, P.; Dannenberg, J. J.; Dapprich, S.; Daniels, A. D.; Farkas, O.; Foresman, J. B.; Ortiz, J. V.; Cioslowski, J.; Fox, D. J. Gaussian 09 (Revision D.01); Gaussian Inc.: Wallingford, CT, 2009.

[6] Andrae, D., Häußermann, U., Dolg, M., Stoll, H., Preuß, H., *Theor. Chim. Acta*, **77**, 123−141 (1990).

[7] (a) Hariharan, P. C., Pople, J. A. *Theor. Chim. Acta*, **28**, 213−222 (1973). (b) Hehre, W. J., Ditchfield, R., Pople, J. A. *J. Chem. Phys*. **56**, 2257 (1972).

[8] (a) Becke, A. D. *Phys. Rev. A: At., Mol., Opt. Phys.* **38**, 3098 (1988). (b) Perdew, J. P. *Phys. Rev. B: Condens. Matter Mater. Phys.* **33**, 8822−8824 (1986).
